# Covalency *versus* magnetic axiality in Nd molecular magnets: Nd-photoluminescence, strong ligand-field, and unprecedented nephelauxetic effect in fullerenes NdM_2_N@C_80_ (M = Sc, Lu, Y)†

**DOI:** 10.1039/d3sc05146c

**Published:** 2023-12-21

**Authors:** Wei Yang, Marco Rosenkranz, Georgios Velkos, Frank Ziegs, Vasilii Dubrovin, Sandra Schiemenz, Lukas Spree, Matheus Felipe de Souza Barbosa, Charles Guillemard, Manuel Valvidares, Bernd Büchner, Fupin Liu, Stanislav M. Avdoshenko, Alexey A. Popov

**Affiliations:** a Leibniz Institute for Solid State and Materials Research (IFW Dresden) 01069 Dresden Germany; b Center for Quantum Nanoscience, Institute for Basic Science (IBS) Seoul Republic of Korea; c ALBA Synchrotron Light Source E-08290 Barcelona Spain f.liu@ifw-dresden.de s.avdoshenko@ifw-dresden.de a.popov@ifw-dresden.de

## Abstract

Nd-based nitride clusterfullerenes NdM_2_N@C_80_ with rare-earth metals of different sizes (M = Sc, Y, Lu) were synthesized to elucidate the influence of the cluster composition, shape and internal strain on the structural and magnetic properties. Single crystal X-ray diffraction revealed a very short Nd–N bond length in NdSc_2_N@C_80_. For Lu and Y analogs, the further shortening of the Nd–N bond and pyramidalization of the NdM_2_N cluster are predicted by DFT calculations as a result of the increased cluster size and a strain caused by the limited size of the fullerene cage. The short distance between Nd and nitride ions leads to a very large ligand-field splitting of Nd^3+^ of 1100–1200 cm^−1^, while the variation of the NdM_2_N cluster composition and concomitant internal strain results in the noticeable modulation of the splitting, which could be directly assessed from the well-resolved fine structure in the Nd-based photoluminescence spectra of NdM_2_N@C_80_ clusterfullerenes. Photoluminescence measurements also revealed an unprecedentedly strong nephelauxetic effect, pointing to a high degree of covalency. The latter appears detrimental to the magnetic axiality despite the strong ligand field. As a result, the ground magnetic state has considerable transversal components of the pseudospin g-tensor, and the slow magnetic relaxation of NdSc_2_N@C_80_ could be observed by AC magnetometry only in the presence of a magnetic field. A combination of the well-resolved magneto-optical states and slow relaxation of magnetization suggests that Nd clusterfullerenes can be useful building blocks for magneto-photonic quantum technologies.

## Abstract 2

Nd-luminescence in NdM_2_N@C_80_ (M = Sc, Lu, Y) reveals the relationship between strain, ligand-field splitting, nephelauxetic effect, and magnetic axiality.

## Graphical abstract

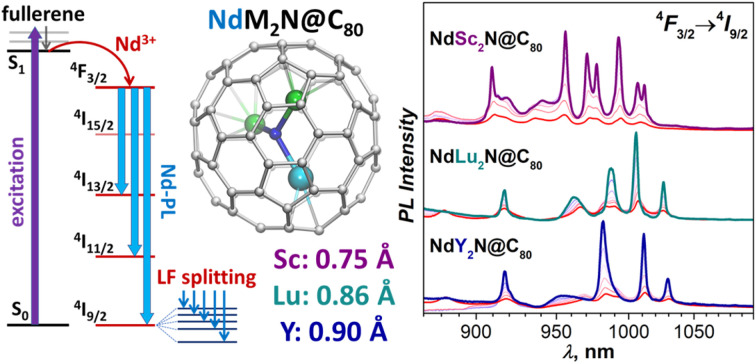

## Introduction

The partially filled 4f-shell in lanthanide compounds is an inexhaustible source of magnetic and optical phenomena with a plethora of already practical and equally numerous prospective applications. The splitting and wavefunction composition of the ligand-field (LF) levels of the lanthanide ground-state multiplet determine the magnetic anisotropy and magnetization relaxation pathways and therefore are of paramount importance for lanthanide-based molecular magnets, such as single-molecule magnets (SMMs). Optical spectroscopy can contribute to the understanding of the SMM properties by the analysis of the fine structure of f–f transitions, in particular in low-temperature photoluminescence (PL) spectra, and by the correlation of the determined level splitting with magnetic behaviour.^1–5^ Since the first report on the Dy(DOTA) complex by Sessoli *et al.* in 2012,^6^ the vast majority of such studies were devoted to Dy^6–17^ and Yb^8,18–25^ complexes, with rare instances of Tb,^8,26–28^ Ho,^29^ Nd,^30–33^ and Er SMMs.^34,35^ Recent trends also include a development of luminescence thermometry based on the temperature-dependent PL of Ln-SMMs,^2,21,29,36,37^ and the use of lanthanide complexes with a narrow optical linewidth for photonic quantum technologies.^38,39^

Lanthanide-based endohedral metallofullerenes (Ln-EMFs) represent an important class of SMMs due to their air and thermal stability, ability to encapsulate Ln ions in rather unusual yet simple chemical environments with strong ligand fields, and stabilization of unconventional bonding interactions between lanthanide ions.^40–44^*Ab initio* computational methods are very instrumental in predicting single-ion magnetic anisotropy in Ln-EMFs,^42,45–52^ but verification of such predictions by direct experimental access to the LF splitting in fullerene-based Ln-SMMs is still missing. A natural prerequisite for the realization of the Ln-based PL in Ln-EMFs is that the lowest-energy fullerene excited state should have higher energy than the lanthanide emitting state. However, the optical gaps of EMFs rarely exceed 1.5 eV. For instance, recent PL studies of the Y_*x*_Sc_3−*x*_N@*I*_h_-C_80_ (*x* = 0–3) family revealed that the energy of the triplet emitting state (T_1_) in this series depends on the Sc content in the endohedral cluster and varies from 1.51 eV in YSc_2_N@C_80_ to 1.68 eV in Y_3_N@C_80_.^53,54^ As M_3_N@*I*_h_-C_80_ clusterfullerenes have the highest optical gaps among EMFs, these energies set the upper limit for Ln-emitting states in Ln-EMFs. The possibility of Ln-based PL in Ln-EMFs is thus essentially restricted to the near-infrared (NIR) range. To date, only different types of Er-EMFs exhibited well-established Er-based NIR luminescence,^55–63^ including the fine structure at low temperatures in some of them, and one study reported on Tm-based NIR-PL in Tm-EMFs.^64^ Unfortunately, Er and Tm tend to have easy-plane type magnetic anisotropy in EMFs^42,65^ and are thus not well suited for the design of EMF-SMMs. The vast majority of EMF-SMMs are based on Tb,^41,42,66–70^ Dy,^43,49,51,71–73^ and Ho,^42,74^ but none of them fulfills the criterion of low Ln-emitting state energy, and thus their Ln-based luminescence is not to be expected unless the fullerene excited states are driven to much higher energies.

Among different lanthanides, Nd seems to be a suitable candidate for Ln-based luminescence in EMF-SMMs. The free-ion energy of the Nd^3+^ NIR-emitting state, ^4^F_3/2_ at 1.45 eV,^75^ is just below the fullerene excited triplet state in M_3_N@*I*_h_-C_80_. Nd_3_N@*I*_h_-C_80_ cannot be produced in a reasonable yield because Nd^3+^ ions are too large,^76^ but this limitation can be circumvented by using mixed-metal analogs with smaller rare-earth ions, such as NdSc_2_N@*I*_h_-C_80_.^77^ The high magnetic anisotropy in nitride clusterfullerenes is caused by the close proximity of the nitride N^3−^ to lanthanide ions, and paramagnetic NMR studies of the LnSc_2_N@C_80_ series accompanied by point-charge ligand field calculations demonstrated that Nd^3+^ in NdSc_2_N@C_80_ has the easy-axis type anisotropy favorable for SMMs.^65^ Nd-EMFs are among the least studied Ln-EMFs, and the first example of the SMM behavior of EMFs with light lanthanides, Nd_2_@C_80_(CF_3_), has only recently been reported.^78^ This lack of studies echoes the situation with light-lanthanide SMMs in general, which are substantially less explored than their heavy-lanthanide congeners.^79,80^ Thus, despite the ubiquitous NIR luminescence known for Nd^III^ complexes and solids, the reports on the combination of slow relaxation of magnetization and Nd-based luminescence remain scarce.^30–33,36,81–85^

In this work, we synthesized a series of NdM_2_N@C_80_ (M = Sc, Lu, Y) molecules to study if the ligand-field experienced by the Nd^3+^ ion and SMM performance can be increased by deliberately shortening the Nd–N bond length and increasing the internal strain induced by complementary M^3+^ metal ions of a different size. By detecting the finely structured Nd-based near-infrared photoluminescence of the NdM_2_N@C_80_ series, we demonstrate the largest ligand-field splitting ever reported for Nd^3+^ in molecular compounds. Furthermore, we show that the ligand field splitting in NdM_2_N@C_80_ is indeed modulated by the increase of the size of M^3+^ ions. However, we also found that shortening of the Nd–N bond not only increases the LF splitting, but also enhances the covalency, which manifests itself in the strongest nephelauxetic effect ever observed in the PL spectra of Nd compounds. The influence of the enhanced LF and covalency on the magnetic and SMM properties of NdSc_2_N@C_80_ is evaluated by SQUID magnetometry and X-ray magnetic circular dichroism.

## Synthesis and molecular structure

### Synthesis and isolation

Metallofullerenes NdM_2_N@C_80_ (M = Sc, Y, Lu) were synthesized by the arc-discharge method using guanidinium thiocyanate as a solid source of nitrogen (see the ESI for further details of synthesis and HPLC separation, Fig. S1–S3†).^86^ The increase of the metal size decreases the propensity of the nitride clusterfullerene formation and thus strongly affects the yield of the arc-discharge synthesis. Thus, in accordance with the ionic radii of M^3+^, the yield of NdM_2_N@C_80_ species follows Sc ≫ Lu > Y. The main efforts were therefore focused on NdSc_2_N@C_80_, which could be accumulated in sufficient amounts for its structural, spectroscopic, and AC/DC magnetic studies. For NdLu_2_N@C_80_ and NdY_2_N@C_80_, as their yield is considerably lower, we only accumulated amounts required for spectroscopic studies. All isolated compounds were characterized by LDI-TOF mass-spectrometry to ensure their compositional purity (Fig. S1–S3†). The UV-Vis-NIR absorption spectra of NdM_2_N@C_80_ showed a typical absorption pattern of M_3_N@C_80_ nitride clusterfullerenes with the *I*_h_ fullerene cage isomer (Fig. S4†); no absorption features caused by f–f transitions of Nd^3+^ could be identified. The vibrational spectra of NdSc_2_N@C_80_ (Fig. S5†) are also in line with the data on other M_3_N@C_80_ compounds.

### Single-crystal X-ray diffraction

The molecular structure of NdSc_2_N@*I*_h_-C_80_ was established by single-crystal X-ray diffraction (SC-XRD) using a co-crystal with nickel(ii) octaethylporphyrin (NiOEP) grown by layering benzene solutions of the fullerene and NiOEP and allowing a slow diffusion thereof for one month. Measurements were performed at 100 K with synchrotron irradiation at the BESSY storage ring (BL14.2, Berlin-Adlershof, Germany).^87^ The XDSAPP2.0 suite was employed for data processing.^88,89^ The structure was solved by direct methods and refined with SHELXL-2018.^90^

**Fig. 1:**
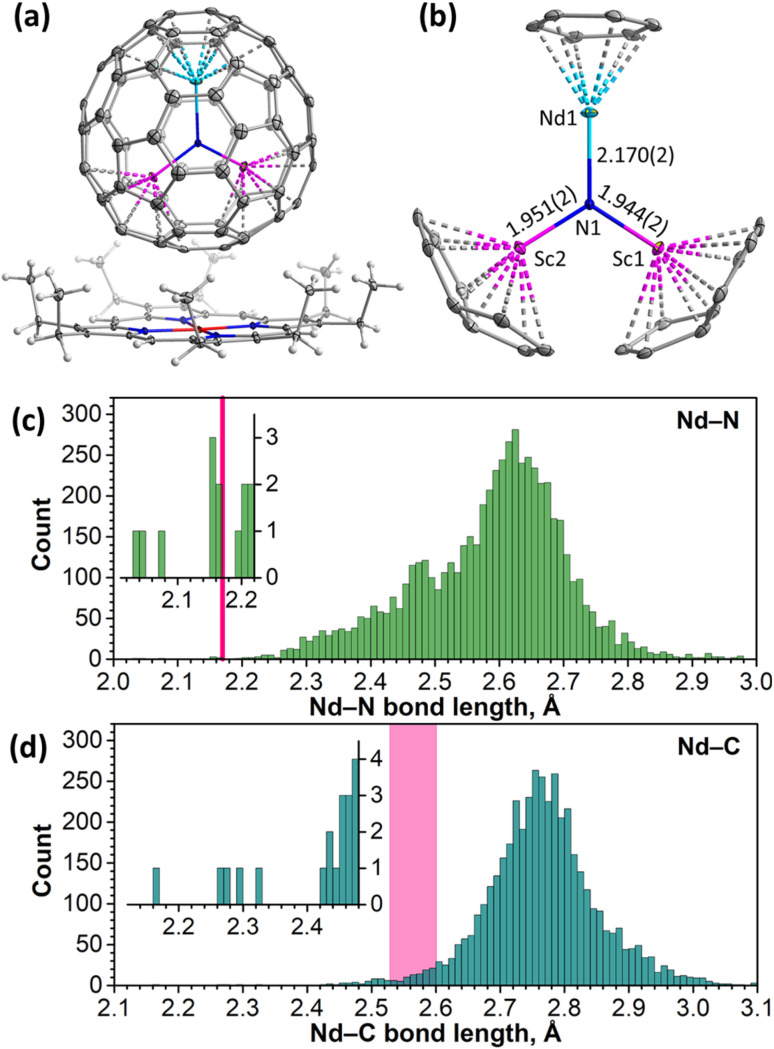
(a) NdSc_2_N@C_80_·NiOEP fragment from the NdSc_2_N@C_80_·NiOEP·2C_6_H_6_ single crystal. (b) NdSc_2_N cluster and fullerene carbons nearest to endohedral metals; Nd–C distances in the Nd-(η^6^-C_6_) fragment are 2.527(2)–2.604(2) Å. Color code: Nd – cyan, Sc – magenta, N – blue, C – gray, and Ni – red. (c) and (d) Histograms of Nd–N (c) and Nd–C (d) bond lengths in molecular compounds deposited in the CCDC database. Insets show expansion of the ranges with the shortest bonds, and the pink line in (c) and the rectangle in (d) denote Nd–N and Nd–C bond lengths in NdSc_2_N@C_80_.

The NdSc_2_N@C_80_·NiOEP·2C_6_H_6_ co-crystal gives a rare example of a fully ordered EMF molecule in the crystal, allowing detailed analysis of the molecular structure (Fig. 1a, S6 and Table S1†). The NdSc_2_N cluster is planar and oriented perpendicular to the porphyrin backbone. Two Sc atoms are located above NiOEP nitrogens, while Nd is facing the opposite side of the fullerene. This orientation of the cluster is typical for MSc_2_N@C_80_ molecules in co-crystals with NiOEP.^91–94^ Nd is coordinated to the fullerene hexagon in an η^6^-fashion with Nd–C distances of 2.527(2)–2.604(2) Å, while the shortest Sc–C bonds of 2.228(3)–2.245(3) Å are to carbons on pentagon/hexagon edges (Fig. 1b).

The large difference of ionic radii^95^ of Nd^3+^ (0.983 Å) and Sc^3+^ (0.745 Å) results in a considerable distortion of the NdSc_2_N cluster, with a 0.320(2) Å displacement of nitrogen from the centroid of the C_80_ cage towards Sc ions. Sc–N bond lengths are 1.944(2) and 1.951(2) Å, while the Nd–N bond length is 2.170(2) Å. Similar structural parameters were found in LnSc_2_N@C_80_ molecules with other light lanthanides, La^91^ (La–N 2.196(4) Å, Sc–N 1.943(6)/1.921(7) Å) and Ce^93^ (Ce–N 2.184(2) Å, and Sc–N 1.942(2)/1.933(2) Å). For heavier lanthanides, the decrease of the Ln^3+^ ionic radius results in a systematic shortening of the Ln–N bond and increase of Sc–N bond lengths. For instance, the Dy–N bond length in DySc_2_N@C_80_ is 2.096(6) Å, while Sc–N bonds are 1.965(6) and 1.978(6) Å.^94^

To consider the structural parameters of NdSc_2_N@C_80_ in a broader context, we analyzed Nd–N and Nd–C bonds for all compounds deposited in the Cambridge Crystallographic Data Centre (CCDC) database. The Nd–N bond length distribution with more than 5800 entries peaks near 2.62 Å (Fig. 1c). The Nd–N bond in NdSc_2_N@C_80_ appears to have one of the shortest lengths in the whole dataset. Shorter Nd–N distances are found only in terminal imides with Nd=N double bonds (2.04–2.08 Å)^96^ and some μ_2_-imide^97,98^ and amide^99^ complexes (2.15–2.17 Å), all reported by Anwander *et al.*

Nd–C bond lengths fall closer to a normal range although being still rather short for their type. For comparison, the peak in the Nd–C bond length distribution (4300 entries) is near 2.75 Å (Fig. 1d). Nd–C distances in complexes with η^*n*^-coordinated arenes span the range of 2.65–2.85 Å for variously substituted η^5^-cyclopentadienyls,^100–102^ 2.60–2.72 Å for η^8^-C_8_H_8_,^103–105^ and 2.845(6)–2.915(7) for η^9^-C_9_H_9_.^104^ In another Nd-EMF characterized by SC-XRD, Nd@*C*_2v_(9)-C_82_, Nd is coordinated to two carbons in the η^2^-fashion, and the corresponding Nd–C bonds are rather short, 2.25–2.30 Å.^106^

### DFT calculations

An increase of the metal size in M_3_N@C_80_ molecules leads to the accumulation of the internal strain, which at first manifests itself in the unusually short M–N bonds, and then changes the M_3_N cluster shape from planar to pyramidal when the M–N bonds reach their limits of shortening.^107,108^ The influence of the metal size on the molecular structures in the NdM_2_N@C_80_ series was analyzed with the help of DFT calculations. As the M_3_N cluster can have somewhat different orientations (conformers) inside the fullerene cage, we first performed a full search of conformers for isolated NdSc_2_N@C_80_ molecules. 120 uniformly distributed NdSc_2_N cluster orientations inside the C_80_ cage were generated using Fibonacci sampling^109^ and used as starting geometries in DFT optimization. The procedure resulted in 6 unique conformers, of which the most stable one (conformer Sc-1) is identical to the SC-XRD structure (see Fig. S7 in the ESI†). Four others are only 2–3 kJ mol^−1^ less stable, while the least stable one (Sc-6) has a relative energy of 9 kJ mol^−1^. For all conformers, DFT calculations predict a planar NdSc_2_N cluster, showing that a comparably small Sc^3+^ ion compensates for the large radius of Nd^3+^. DFT-optimized Nd–N bond lengths in all conformers are in the range of 2.22–2.23 Å and overestimated compared to the experimental value by 0.05 Å.

When Sc^3+^ is substituted by larger Lu^3+^ (0.861 Å) and Y^3+^ (0.900 Å), the Nd–N bond lengths in the most stable DFT conformers of NdLu_2_N@C_80_ (Lu-1) and NdY_2_N@C_80_ (Y-1) are shortened to 2.165 Å and 2.170 Å, respectively (Fig. S8 and S9†). These similar values for metals of different sizes indicate that the Nd–N bond has reached its shortening limit. As a result, the NdM_2_N cluster cannot sustain a planar shape in NdM_2_N@C_80_ and becomes pyramidal. In different conformers of NdLu_2_N@C_80_ and NdY_2_N@C_80_, the nitrogen is elevated above the NdM_2_ plane by 0.37–0.51 Å and 0.52–0.62 Å, respectively (Fig. S8 and S9†).

### Photoluminescence and ligand-field splitting

**Fig. 2:**
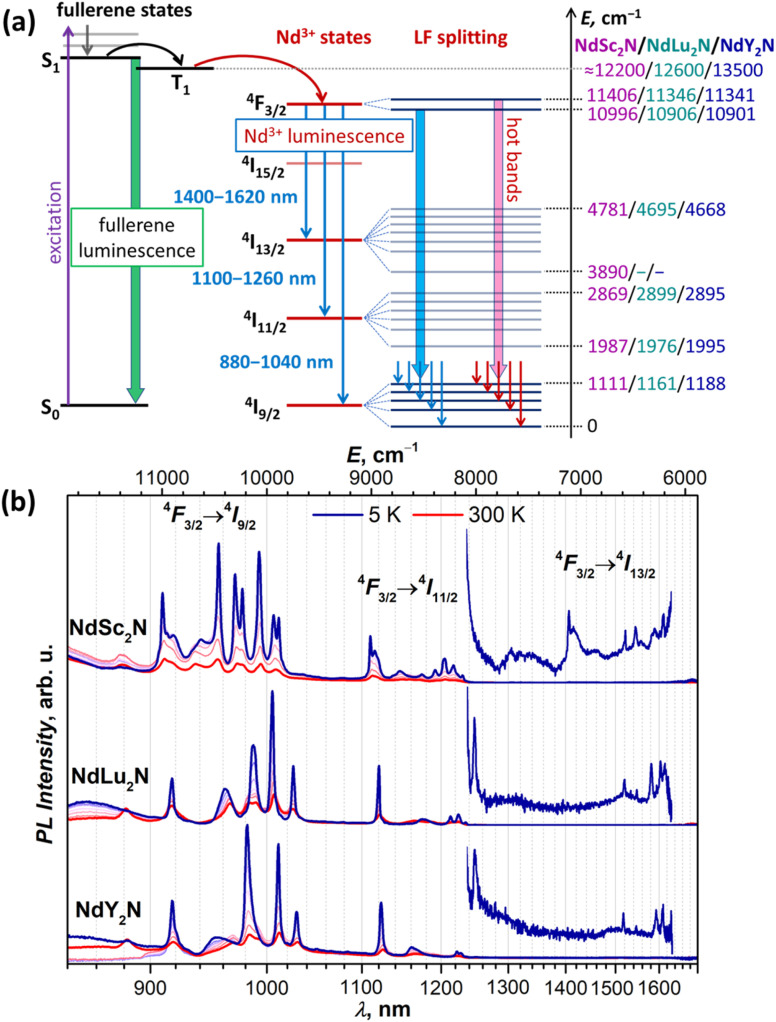
(a) Schematic description of the Nd-based luminescence in NdM_2_N@C_80_. In non-4f M_3_N@C_80_, a fullerene-based excitation is followed by an internal conversion to the singlet state S_1_, which then undergoes intersystem crossing to the triplet state T_1_, and both S_1_ and T_1_ participate in the radiational decay in the form of thermally activated delayed fluorescence and phosphorescence.^53^ When one M in the endohedral cluster is replaced with Nd, the energy is further transferred from the T_1_ state to the 4f-shell of Nd, which then emits from the ^4^*F*_3/2_ state giving three near-infrared ^4^F_3/2_ → ^4^I_*J*_ PL bands (*J* = 9/2, 11/2, 13/2). Their fine structure is caused by the ligand-field splitting in each ^4^I_*J*_ multiplet, while hot bands appear when the LF-excited state in the ^4^F_3/2_ doublet has significant thermal population. (b) Luminescence spectra of solid NdM_2_N@C_80_ (M = Sc, Lu, Y) measured at 5–300 K with 50 K steps using laser excitation at 488 nm. The energy values listed on the right scale in (a) are obtained from PL measurements at 5 K, and the LF splitting of ^4^F_3/2_ is estimated from hot bands at 300 K (see Fig. 3a and S12–S17†), while conservative estimations of fullerene T_1_ energies are based on the VT-PL data on YSc_2_N@C_80_ and Y_3_N@C_80_ from ref. 53 and preliminary VT-PL measurements of Lu_3_N@C_80_.

Short Ln–N bonds along with the large negative charge of the nitride ion lead to a strong axial ligand field imposed on lanthanide ions in nitride clusterfullerenes. To address the size of LF splitting in Nd-EMFs experimentally, we turned to photoluminescence spectroscopy. The photophysical behavior of MSc_2_N@C_80_ molecules without a partially filled 4f-shell in the metal can be illustrated by YSc_2_N@C_80_.^53^ After photoexcitation and internal conversion to S_1_, it undergoes a fast intersystem crossing to the T_1_ state located at 1.5–1.6 eV above the ground state. However, the energy difference between S_1_ and T_1_ is only 0.03 eV, facilitating inversed intersystem crossing. As a result, YSc_2_N@C_80_ exhibits thermally activated delayed fluorescence at room temperature and down to 30 K. At lower temperature, the thermal repopulation of the S_1_ state from T_1_ is frozen, leaving weak phosphorescence as the only radiational decay process.^53^ However, when the endohedral cluster contains Nd instead of Y, the energy can be further transferred from the fullerene-based T_1_ state to the 4f-shell of the lanthanide and produce Nd-based NIR luminescence with the origin at the ^4^F_3/2_ state (Fig. 2a). Indeed, our PL measurements of NdSc_2_N@C_80_ showed very characteristic Nd-centered NIR luminescence with three bands caused by ^4^F_3/2_ → ^4^I_9/2_, ^4^F_3/2_ → ^4^I_11/2_, and ^4^F_3/2_ → ^4^I_13/2_ transitions (Fig. 2b). Similar luminescence spectra were also observed for NdLu_2_N@C_80_ and NdY_2_N@C_80_ (Fig. 2b). As far as we know, this is the first observation of Nd-based luminescence in endohedral metallofullerenes.

The PL spectra of NdM_2_N@C_80_ are remarkable in several aspects. First, the ^4^F_3/2_ → ^4^I_*J*_ PL bands are shifted from 900 nm, 1060 nm, and 1350 nm, at which they are usually centered in Nd^3+^ complexes,^110^ to longer wavelengths. Particularly, the characteristic Nd^3+^ emission at 1060 nm used in solid-state lasers is “missing” as the ^4^F_3/2_ → ^4^I_11/2_ PL band of NdM_2_N@C_80_ occurs at 1100–1250 nm (Fig. 2b). As we discuss below, this shift of PL bands is the manifestation of a strong nephelauxetic effect. Second, the bands have atypical intensity distribution. Normally, the ^4^F_3/2_ → ^4^I_11/2_ band is the strongest one, followed by ^4^F_3/2_ → ^4^I_9/2_ with 2–4 times lower intensity, while the ^4^F_3/2_ → ^4^I_13/2_ band is the weakest. In NdM_2_N@C_80_, the latter is also very weak, but the ^4^F_3/2_ → ^4^I_9/2_ band is considerably stronger than ^4^F_3/2_ → ^4^I_11/2_. Third, the luminescence lifetimes of NdM_2_N@C_80_ are very short. PL lifetimes in Nd complexes are usually found in the 0.2–2 μs range. For the polycrystalline film of NdSc_2_N@C_80_, the PL decay is biexponential with the lifetimes of 4.5 ns/13 ns at room temperature, and 4 ns/19 ns between 200 and 5 K, with the longer component having the main contribution with a weight of 80% (Fig. S10†). To check if this fast decay can be caused by intermolecular Nd⋯Nd quenching, the fullerene was diluted in a polystyrene matrix. In the latter, the decay was mono-exponential with the lifetime near 20 ns. Polycrystalline NdLu_2_N@C_80_ and NdY_2_N@C_80_ showed monoexponential PL decay with very weak temperature dependence and lifetimes of 10.5–11.0 ns and 9.0–9.5 ns, respectively (Fig. S11†). These short lifetimes suggest a low quantum yield and explain the weak PL intensity of NdM_2_N@C_80_. Fourth, each PL band of NdM_2_N@C_80_ exhibits unusually broad and well-resolved fine structure already at room temperature. Upon cooling, the narrowing of the lines and increase of their intensity took place down to 50–100 K, while further cooling did not bring considerable changes in the spectra (Fig. 2b and S12–S17†).

#### LF splitting in PL spectra

**Fig. 3:**
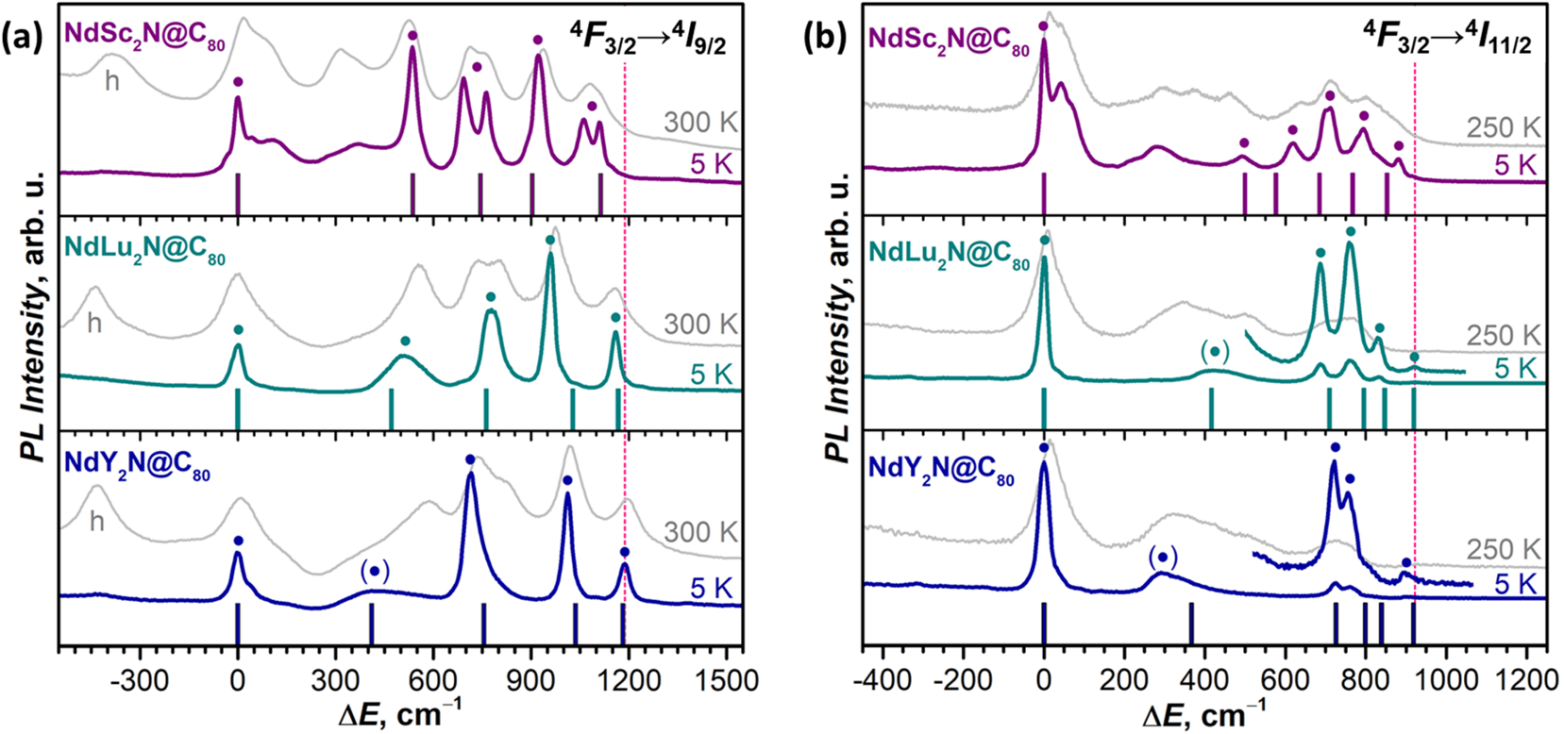
The fine structure of (a) ^4^F_3/2_ → ^4^I_9/2_ and (b) ^4^F_3/2_ → ^4^I_11/2_ photoluminescence bands of NdM_2_N@C_80_ (M = Sc, Lu, Y) measured at 5 K and 300 K (250 K). Vertical bars are the CASSCF-computed energies of LF states in ^4^I_9/2_ (a) and ^4^I_11/2_ (b) multiplets in Sc-3, Lu-3, and Y-3 conformers scaled by 1.15 to account for the systematic underestimation. The peaks assigned to pure electronic ^4^F_3/2_(KD1) → ^4^I_*J*_(KD*n*) transitions are labeled with ● and broad peaks with uncertain assignment to KD2 are labelled with (●). The Δ*E* scale for each NdM_2_N@C_80_ is given *versus* the peak of the lowest-energy Kramers doublet in the ^4^I_9/2_ or ^4^I_11/2_ multiplet. In (a), the peaks at negative Δ*E* in 300 K spectra labelled with “h” are hot bands caused by the ^4^F_3/2_(KD2) → ^4^I_9/2_(KD1) transition of each NdM_2_N@C_80_, which allow estimation of *Δ*_1,2_ in the ^4^F_3/2_ multiplet. The intensity of 5 K and higher-temperature spectra is given off-scale (see Fig. 2b and S12–S17† for the real relative intensity at different temperatures). To guide the eye, the vertical dashed line marks the highest energy KD in the ^4^F_3/2_ → ^4^I_9/2_ band (NdY_2_N@C_80_) and in the ^4^F_3/2_ → ^4^I_11/2_ band (NdLu_2_N@C_80_).

The fine structure of PL bands is of primary interest for this work since it represents the LF splitting of ^4^I_*J*_ multiplets. In the ^4^F_3/2_ → ^4^I_*J*_ band, one can expect (2*J* + 1)/2 lines corresponding to the number of Kramers doublets (KDs) in the ^4^I_*J*_ multiplet. However, when not only KD1, but also KD2 of the emitting ^4^F_3/2_ state has a significant population, transitions originating from ^4^F_3/2_ (KD2) and known as hot bands can increase the number of detectable PL features (Fig. 2a). Further complication may be caused by vibronic transitions, involving vibrational excitations in emitting or final states. Finally, different sites, by which we understand molecules with different surroundings or with different orientations of the endohedral cluster (conformers), may also have unequal energy levels and thus multiply the number of lines in PL spectra. To aid the interpretation of PL spectra, we performed variable-temperature measurements (Fig. 2 and 3 and S12–S17†) since hot bands decrease their intensity and eventually disappear upon cooling. Likewise, zero-phonon electronic transitions tend to exhibit pronounced narrowing at low temperatures, while the accompanying phonon sidebands usually remain broader.

At low temperatures, the ^4^F_3/2_ → ^4^I_9/2_ band of NdSc_2_N@C_80_ is reduced to seven narrow peaks, of which three are stand-alone and four form two doublets with the splitting of 70 and 50 cm^−1^ (Fig. 3a). We assign these peaks to five pure electronic ^4^F_3/2_(KD1) → ^4^I_9/2_(KD*n*) transitions. The splitting of KD3 and KD5 features into doublets is likely caused by a coexistence of two emitting sites,^53,111^ but the lack of analogous splitting for other KDs is not clear. Two broad peaks located between KD1 and KD2, at Δ*E* = 115 and 370 cm^−1^, can be tentatively assigned to vibronic transitions, but may also correspond to different conformers (*vide infra*). The low-*T* PL spectrum thus allows determination of experimental *Δ*_1,2_ and *Δ*_LF_ values in NdSc_2_N@C_80_ as 536 cm^−1^ and 1061/1110 cm^−1^. The room-temperature PL spectrum also features a peak at Δ*E* = −410 cm^−1^, which disappears upon cooling (Fig. 3a). This hot band is likely caused by the ^4^F_3/2_(KD2) → ^4^I_9/2_(KD1) transition and thus allows estimation of the LF splitting in the ^4^F_3/2_ multiplet.

The interpretation of the ^4^F_3/2_ → ^4^I_9/2_ PL bands of NdLu_2_N@C_80_ and NdY_2_N@C_80_ is more straightforward as they show neither pronounced vibronic features nor additional splitting (Fig. 3 and Table S2†). The five peaks in low-*T* spectra are thus readily assigned to five KDs of the ^4^I_9/2_ multiplet. The only peculiarity is the strongly enhanced linewidth of the KD2 peak, which is still well-defined for NdLu_2_N@C_80_, but is barely discernible for NdY_2_N@C_80_. Such a strong broadening indicates that this state experiences very fast relaxation even at 5 K. The LF splitting of ^4^I_9/2_ in the NdM_2_N@C_80_ series increases systematically with the size of the M ion from NdSc_2_N@C_80_ (*Δ*_LF_ = 1111 cm^−1^) to NdLu_2_N@C_80_ (1161 cm^−1^) and further to NdY_2_N@C_80_ (1188 cm^−1^). The LF splitting of the ^4^F_3/2_ multiplet is also larger for Lu and Y and amounts to 440 cm^−1^*versus* 410 cm^−1^ for Sc. At the same time, *Δ*_1,2_ decreases from 536 cm^−1^ (Sc) to 510 cm^−1^ (Lu) and 420 cm^−1^ (Y). The energies of other KDs also vary considerably in the NdM_2_N@C_80_ series. Besides, not only the energies, but also the intensities of transitions are quite different from Sc to Lu to Y, which indicates that the state composition is also considerably affected by the internal strain.

Thus, analysis of the ^4^F_3/2_ → ^4^I_9/2_ PL band demonstrates that enclosing Nd^3+^ in a molecular environment with a very short Nd–N bond and further increasing the internal strain by changing the size of the NdM_2_N cluster indeed result in a record-high LF splitting in NdM_2_N@C_80_ compounds exceeding 1100 cm^−1^. For comparison, a resolved structure of LF levels allowing estimation of *Δ*_LF_ for the Nd-^4^I_9/2_ multiplet was observed in the optical spectra of five Nd-SMMs, including NdTp_3_,^112,113^ acetato-diphenoxo bridged {ZnNd} complex,^30^ dinuclear {Nd_2_} complexes with 9-anthracenecarboxyl and 2,2′-bipyridine^31^ or phenanthroline^32^ ligands, and in the Nd complex with *N*-(diphenylphosphoryl)pyrazine-2-carboxamide.^33^ None of them had the *Δ*_LF_ exceeding 460 cm^−1^. Among the Nd-SMMs studied a*b initio*, *Δ*_LF_ values exceeding 500 cm^−1^ were reported only for three Nd-SMMs: metallocenium complex [Nd(Cp^ttt^)_2_]B(C_6_F_5_)_4_ (*Δ*_LF_ = 672 cm^−1^ and *Δ*_1,2_ = 130 cm^−1^),^100^ Nd complex with equatorial metallacrowns and axial *n*-Bu_3_PO ligands in hexagonal bipyramidal coordination (*Δ*_LF_ = 586 cm^−1^ and *Δ*_1,2_ = 104 cm^−1^),^114^ and (COT)Nd(Cp^ttt^) sandwich complex (*Δ*_LF_ = 545 cm^−1^ and *Δ*_1,2_ = 78 cm^−1^).^115^ Among non-SMMs, a very large LF splitting almost rivaling the results of this work was determined by Amberger *et al.* based on absorption and PL spectra and phenomenological crystal-field modelling in some tris(η^5^-cyclopentadienyl)Nd(iii) complexes, such as Nd(η^5^-C_5_Me_5_)_3_ (*Δ*_LF_ = 861 cm^−1^),^116^ Nd(η^5^-C_5_Me_4_H)_3_ (*Δ*_LF_ = 1000 cm^−1^),^117^ Nd(η^5^-C_5_H_4_^*t*^Bu)_3_ and Nd(η^5^-C_5_H_4_SiMe_3_)_3_ (in both, *Δ*_LF_ = 1030 cm^−1^).^118^ The SMM properties of these complexes were not studied, but the ground state with the dominating |±5/2〉 term (see the ESI for results of *ab initio* calculations†) suggests that the slow relaxation of magnetization can hardly be expected.

In addition to the ground-state LF structure, well-resolved PL spectra also enable analysis of the ^4^I_11/2_ multiplet. For NdSc_2_N@C_80_, the experimental KD2 is found at 491 cm^−1^, whereas the overall *Δ*_LF_(^4^I_11/2_) splitting is 880 cm^−1^, considerably smaller than *Δ*_LF_(^4^I_9/2_) of 1111 cm^−1^. Broad features in the gap between KD1 and KD2 at 50 cm^−1^ and 280 cm^−1^ can be preliminary assigned to vibronic transitions. In NdLu_2_N@C_80_ and NdY_2_N@C_80_, the ^4^F_3/2_ → ^4^I_11/2_ band is dominated by the ^4^F_3/2_(KD1) → ^4^I_11/2_(KD1) transition, whereas other features have much lower intensity (Fig. 2b and 3b). Similar to the ^4^F_3/2_ → ^4^I_9/2_ band, the ^4^I_11/2_(KD2) peaks appear strongly broadened, which makes their assignment less certain. The *Δ*_LF_(^4^I_11/2_) splitting of 920 cm^−1^ in NdLu_2_N@C_80_ and 900 cm^−1^ in NdY_2_N@C_80_ exceed that in NdSc_2_N@C_80_.

A complete set of ^4^F_3/2_ → ^4^I_13/2_ transitions can be identified only for NdSc_2_N@C_80_ (Fig. 2b and Table S2†). Seven KDs of the ^4^I_13/2_ multiplet span the energy range of 890 cm^−1^ and show similar motifs to the ^4^F_3/2_ → ^4^I_11/2_ band with a large gap between KD1 and KD2 of 530 cm^−1^ and a dense group of other KDs at higher energy. For NdLu_2_N@C_80_ and NdY_2_N@C_80_, the position of the KD1 peak cannot be determined because of its very low intensity (Fig. 2b), which prevents further analysis of the LF splitting in ^4^I_13/2_.

#### *Ab initio* calculations of LF splitting

**Fig. 4:**
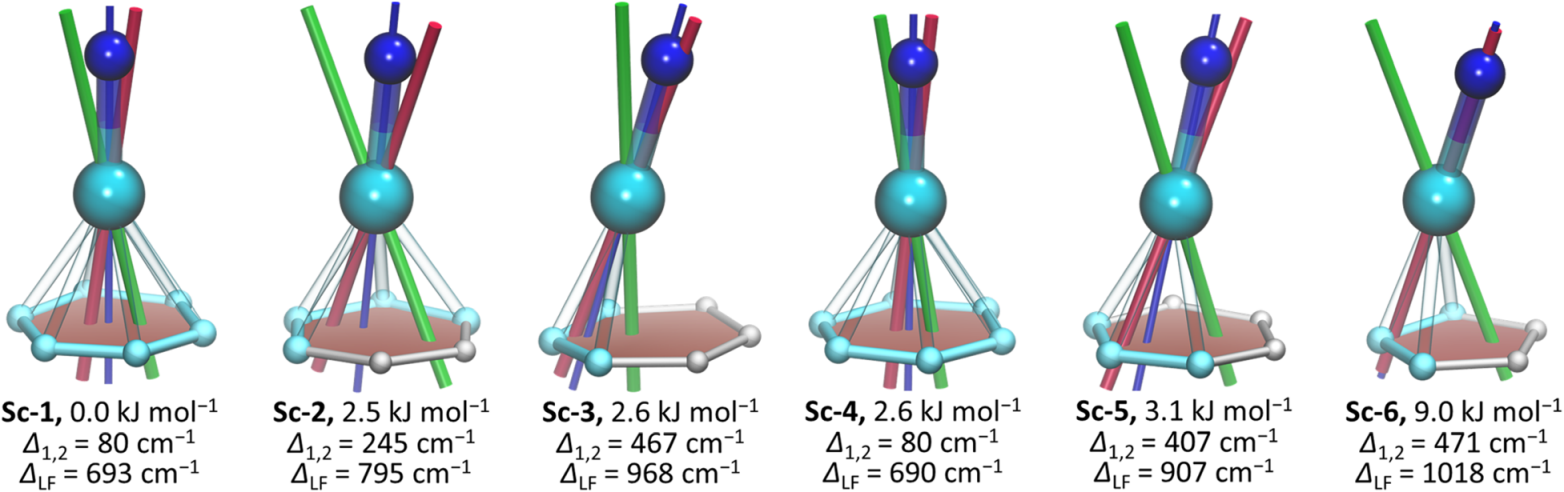
Coordination of Nd atoms to the closest carbon atoms in six conformers of NdSc_2_N@C_80_, their geometrical Nd–N axes (thin blue lines) and *g*_z_ axes of the two lowest-energy Kramers doublets from CASSCF calculations (KD1 – red lines and KD2 – green lines). Color code: Nd – cyan, N – blue, and C – light cyan (*d*_Nd–C_ ≤ 2.6 Å) or light gray (*d*_Nd–C_ > 2.6 Å). Nd–C distances shorter than 2.6 Å are shown as bonds. Also listed for each conformer are its relative energy from DFT calculations and *Δ*_1,2_ and *Δ*_LF_ splitting from CASSCF calculations.

To analyze the composition of LF-split states, we performed *ab initio* calculations at the CASSCF(3,7)/RASSI level. While the lowest-energy conformer of NdSc_2_N@C_80_ corresponds to the SC-XRD structure in the NdSc_2_N@C_80_·Ni(OEP) co-crystal (Fig. S7†), the energy span of only 9 kJ mol^−1^ predicted by DFT for other conformers is quite small, and other conformers can also be present in powder samples studied by PL (and later by magnetometry). Therefore, calculations were performed for all six DFT-computed conformers of NdSc_2_N@C_80_. Our earlier *ab initio* studies of LF in conformers of various Dy-SMMs showed that the influence of the lanthanide coordination to the fullerene cage is noticeable but not decisive.^51,119,120^ Unexpectedly, the situation found here for NdSc_2_N@C_80_ is quite dissimilar. The *Δ*_LF_ splitting of the ^4^I_9/2_ multiplet in different conformers varies from 690 to 1018 cm^−1^, and even larger relative variation is found for *Δ*_1,2_, which spans from 80 to 470 cm^−1^ (Fig. 4 and Table S3†).

The variation of the LF splitting can be correlated with the Nd-fullerene coordination motif (Fig. 4). The smallest *Δ*_1,2_ of 80 cm^−1^ is found for conformers Sc-1 and Sc-4, in which Nd is coordinated to the center of a fullerene hexagon in an η^6^-manner. Even a small shift of Nd from the hexagon center toward a C–C bond yields a 3-fold increase of *Δ*_1,2_ in Sc-2 (hapticity η^4^), while the more pronounced shift toward one of the carbon atoms increases *Δ*_1,2_ to 407 cm^−1^ in Sc-5 (η^3^) and further to 467 cm^−1^ in Sc-3 (η^3^). The largest *Δ*_1,2_ and *Δ*_LF_ values are achieved when Nd faces one carbon atom in Sc-6 (hapticity between η^1^ and η^3^). The deviation of the quantization axis of the ground-state Kramers doublet (KD1) from the Nd–N bond axis (Fig. 4) is another manifestation of the strong influence of the Nd-fullerene coordination. The two axes coincide only in Sc-6, while the angle between them in other conformers reaches 13°.

**Fig. 5:**
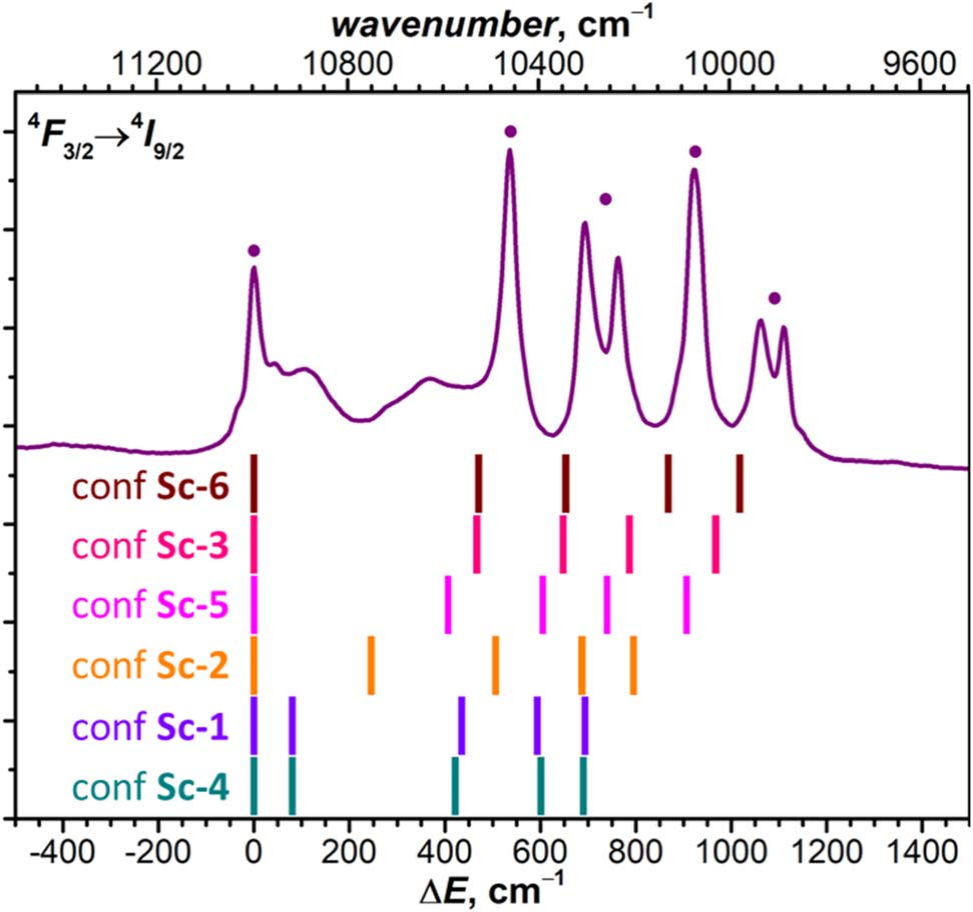
The fine structure of ^4^F_3/2_ → ^4^I_9/2_ photoluminescence bands of NdSc_2_N@C_80_ measured at 5 K compared to LF splitting of the ^4^I_9/2_ multiplet in six conformers of NdSc_2_N@C_80_ from CASSCF calculations (see Tables S3b–S3f† for numerical values).

The comparison of theoretical LF splitting to the fine structure of PL spectra shows that only conformers Sc-3 and Sc-6 agree reasonably well with experimental data (Fig. 5 and S18†), while predictions for other conformers, including the lowest-energy Sc-1, are far off. Thus, it appears that only the conformers, in which Nd is coordinated close to one C atom in the η^1^–η^3^ manner, contribute to the fine structure of PL spectra. The only features which might be attributed to other conformers of NdSc_2_N@C_80_ are broad peaks in the gap between 0 and 500 cm^−1^ (KD1 and KD2, Fig. 5), for which our preferred assignment is vibronic transitions. It is hard to determine at this moment whether other conformers have more efficient non-radiative decay channels and do not show up in the spectra, or the CASSCF predictions for these conformers are incorrect, or they are destabilized in powder samples. The technical aspects of CASSCF computations also indicate some untrivial behavior. For some conformers, the CASSCF wavefunction was found seamlessly, while for others, they were sensitive to initial guesses, and it took an effort to converge the wavefunction to a decent 4f-pure state.

Following the calculation results on NdSc_2_N@C_80_, for NdLu_2_N@C_80_ and NdY_2_N@C_80_ we performed CASSCF calculations for two representative conformers, conformer Lu(Y)-1 with η^6^-type coordination of Nd, and conformer Lu(Y)-3 with η^3^-pentagon-coordinated Nd (see Fig. S19†). The difference between LF splitting of conformers is also considerable but less pronounced than in NdSc_2_@C_80_ (Tables S7 and S8†), and the conformer Lu(Y)-3 provides better match to the fine structure in experimental PL spectra (Fig. 3). Remarkably, while the total LF splitting in NdM_2_N@C_80_ increases with the size of M, the *Δ*_1,2_ value exhibits the opposite trend and shows a gradual decrease, which is correctly captured by CASSCF calculations for Lu(Y)-3 (Fig. 3).

#### LF splitting, magnetic axiality, and covalency

The aims we followed in creating the strongly strained environment around the Nd–N bond was to achieve a large LF splitting and enhanced magnetic axiality. While the LF splitting in NdM_2_N@C_80_ is indeed unprecedentedly large, the problem of axiality appears to be more complicated. For the subsequent analysis, we use conformer Sc-3. Table 1 summarizes the energies of its Kramers doublets (KDs) in the ground-state Nd^3+-4^I_9/2_ multiplet, along with their composition in the |*m*_*J*_〉 basis and principal components of pseudospin g-tensors. Analogous data for other conformers can be found in Tables S3, S7, and S8 in the ESI.† Aside from the systematic underestimation of the LF splitting by 10–15%, the calculated spectrum agrees well with the experimental data for all spectrally characterized multiplets (Fig. 3 and S18†). Besides, the *ab initio Δ*_1,2_ value computed for Sc-3 in the ^4^F_3/2_ multiplet is 401 cm^−1^, also in good agreement with the experiment.

**Table 1:** Ligand-field splitting of the Nd^3+-4^I_9/2_ multiplet and pseudospin g-tensors of Kramers doublets (KDs) in NdSc_2_N@C_80_ (conformer Sc-3) computed at the CASSCF/RASSI level and with the point-charge model

KD	Exp. *E*^a^ cm^−1^	Calc. *E* cm^−1^	% Composition in the |*m*_*J*_〉 basis (*J* = 9/2) b	*g*_x_	*g*_y_	*g*_z_	*φ*° c
			CASSCF/RASSI				
KD1	0	0	85|±9/2〉 + 7|±5/2〉 + 6|±3/2〉	0.0371	0.0445	5.6890	—
KD2	536	467	48|±5/2〉 + 25|±7/2〉 + 19|±3/2〉 + 8|±1/2〉	0.5312	1.3555	3.1828	21
KD3	693/763	648	31|±3/2〉 + 29|±5/2〉 + 19|±7/2〉 + 14|±9/2〉	0.0369	1.0730	3.6643	25
KD4	923	786	48|±7/2〉 + 30|±3/2〉 + 14|±1/2〉 + 8|±5/2〉	2.0524	0.3009	3.7618	55
KD5	1061/1110	968	70|±1/2〉 + 14|±3/2〉 + 8|±5/2〉 + 8|±7/2〉	4.3734	2.4200	0.4013	7
			Point-charge model				
KD1	0	0	99.9|±9/2〉	0.0000	0.0000	6.5437	—
KD2	536	466	99.6|±7/2〉	0.0013	0.0014	5.0823	3
KD3	693/763	643	99.0|±5/2〉	0.0937	0.1002	3.6158	1
KD4	923	770	95.0|±3/2〉 + 4.4|±1/2〉	1.4408	1.4927	2.0604	7
KD5	1061/1110	867	94.8|±1/2〉 + 4.5|±3/2〉	5.0352	2.0948	0.6166	0

a Experimental values are from photoluminescence measurements at 5 K (see Fig. 3a).

b For each KD, 4 main terms exceeding 5% are shown in the descending order of their contribution.

c *φ* is the angle between the *g*_z_ direction of the given KD and that of the ground state KD1.

The strongly axial LF for 4f-prolate Kramers ions (Ce^3+^, Nd^3+^, and Dy^3+^) normally leads to the high purity of KDs in the |*m*_*J*_〉 representation, with the ground state featuring the largest *J*_z_ projection. It also implies that pseudospin g-tensors of KDs have small transversal *g*_x,y_ components, colinear principal *g*_z_ axes, and *g*_z_ values close to 2|*m*_*J*_|*g*_J_, where *g*_J_ is the Landé *g*-factor. The description we infer from *ab initio* calculations of Sc-3 is quite different (Table 1; see Table S3† for other conformers). KD1 indeed resembles the |±9/2〉 state with 85% contribution and the quantization axis aligned along the Nd–N bond with a deviation of 4.1°. The *g*_z_ and *g*_x,y_ values of KD1 are 5.689 and near 0.04, respectively, which can be compared to *g*_z_ = 6.545 and *g*_x,y_ = 0 expected for the pure |±9/2〉 doublet. However, higher-energy KDs have strongly mixed |*m*_*J*_〉 composition and enhanced *g*_x,y_ components, while their *g*_z_ axes are rotated away from that of KD1. In contrast, the *ab initio* description of the isostructural DySc_2_N@C_80_ is entirely consistent with expectations for a strongly axial ligand field.^45,120^ The ground state KD1 has 99.7% weight of the |±15/2〉 state and *g*_x,y_ is less than 10^−4^, while *g*_z_ = 19.84 (*versus* 20.00 expected for the pure *m*_*J*_ = ±15/2 state).^120^ The next three KDs of DySc_2_N@C_80_ also have high contribution of one of the |±*m*_*J*_〉 states (>80%), and the principal *z* axes of their pseudospin g-tensors are nearly collinear. The overall LF splitting in DySc_2_N@C_80_ is 1284 cm^−1^.

Thus, the molecular environment, in which the strong axiality is expected and indeed realized for Dy, produces quite different properties for Nd. We can recall that Nd^3+^ has considerably larger Steven's factor *γ*_j_ (−38 × 10^6^) than Dy^3+^ (1.03 × 10^6^), which increases the weight of *B*_*q*_^*k*^ terms with *k =* 6 in the ligand field. While for DyM_2_N@C_80_ molecules, *B*_*q*_^2^, *B*_*q*_^4^, and *B*_*q*_^6^ contribute 65–80%, 5–10%, and 10–25% to *ab initio* computed LF splitting, respectively, in NdM_2_N@C_80_ the balance changes to similar contributions of *B*_*q*_^2^ and *B*_*q*_^6^ near 40–45%, while *B*_*q*_^4^ terms cover 10–15%. The enhanced *B*_*q*_^6^ weight implies the higher sensitivity of the Nd^3+^ ligand field to the environment, yet the deviation from axiality in NdM_2_N@C_80_ is too strong and cannot be explained by the *γ*_j_ factor alone. To understand if low symmetry or unobvious peculiarities of the electrostatic distribution may play a role, we obtained the LF parameters of NdSc_2_N@C_80_ using a point-charge model with LoProp^121^ charges from CASSCF calculations scaled by a factor of 1.08 to reproduce the *ab initio Δ*_1,2_ value. The KD energies calculated with such a LF Hamiltonian are not far from *ab initio* LF energies (Table 1), but the states are described by almost pure |*m*_*J*_〉 functions, and KD1–KD3 have *g*_z_ values close to 2|*m*_*J*_|*g*_J_. Similar results with a slightly higher degree of *m*_*J*_-mixing were obtained when the model was adjusted by treating the Nd^3+^ ion quantum chemically at the CASSCF/RASSI level, while keeping point charges instead of all other atoms (Table S5†). From these models, we infer that the electrostatic environment around Nd^3+^ indeed favors LF axiality, while deviations from axiality in NdSc_2_N@C_80_ should be caused by non-electrostatic contributions, such as the covalency.^122–124^ Indeed, when CASSCF/RASSI computations were performed for the (NdSc_2_N)^6+^ cluster augmented with point charges mimicking the carbon atoms of the fullerene cage, a considerable state mixing was observed (Table S5c†). We thus conclude that the expansion of the valence space from Nd^3+^ through NdSc_2_N^6+^ to NdSc_2_N@C_80_ results in the systematic increase of the *m*_*J*_ mixing in Nd KDs and decrease of the axiality. Significant changes of the LF splitting induced by subtle variation of the local Nd environment in NdSc_2_N@C_80_ conformers also cannot be expected based on simple electrostatic considerations. Point-charge calculations for the conformer Sc-1 do not follow CASSCF results and predict similarly large KD2 energy as in Sc-3 (compare values in Table S5†). These facts highlight the importance of valence interactions and show that pure electrostatic models would be inappropriate for the description of Nd-EMF SMMs.

Comparison to other coordination environments, in which heavy lanthanide analogs exhibit very high magnetic axiality, reveals that the situation found here for NdSc_2_N@C_80_ is not unique for fullerenes but rather describes a common behaviour of Nd in molecular magnets. For instance, [Nd(H_2_O)_5_L_2_]^3+^ complexes with pentagonal bipyramidal coordination and axial phosphine oxide ligands,^125,126^ or Nd metallocenium complexes,^100^ all have the ground state KD with enhanced |±9/2〉 contribution (86–99%), but their higher-energy KDs are mixed considerably in the |*m*_*J*_〉 representation. Likewise, the *g*_x,y_ values of 0.01–0.02 found in KD1 of these complexes are small in comparison to *g*_z_ values near 6.3, but appear quite large when compared to transversal g-tensor components of 10^−4^ in KD1 of similar Dy complexes. In other Nd-SMMs, for which *ab initio* calculations were performed, the degree of axiality is considerably smaller.

#### Nd–N distance and LF splitting

The discussion above showed that by creating unusually short Nd–N bonds and a highly strained endohedral environment, we achieved large LF splitting but simultaneously increased the degree of covalency and decreased the magnetic axiality. As coordination of Nd to the fullerene π-system also played a considerable role, it is hard to disentangle it from the influence of Nd–N bonding. To study the isolated effect of the extreme shortening of the Nd–N bond, we used the NdN molecule as a toy system and computed LF splitting at different Nd–N distances with a point charge model and *ab initio* at the CASSCF(3,7)/RASSI level.

**Fig. 6:**
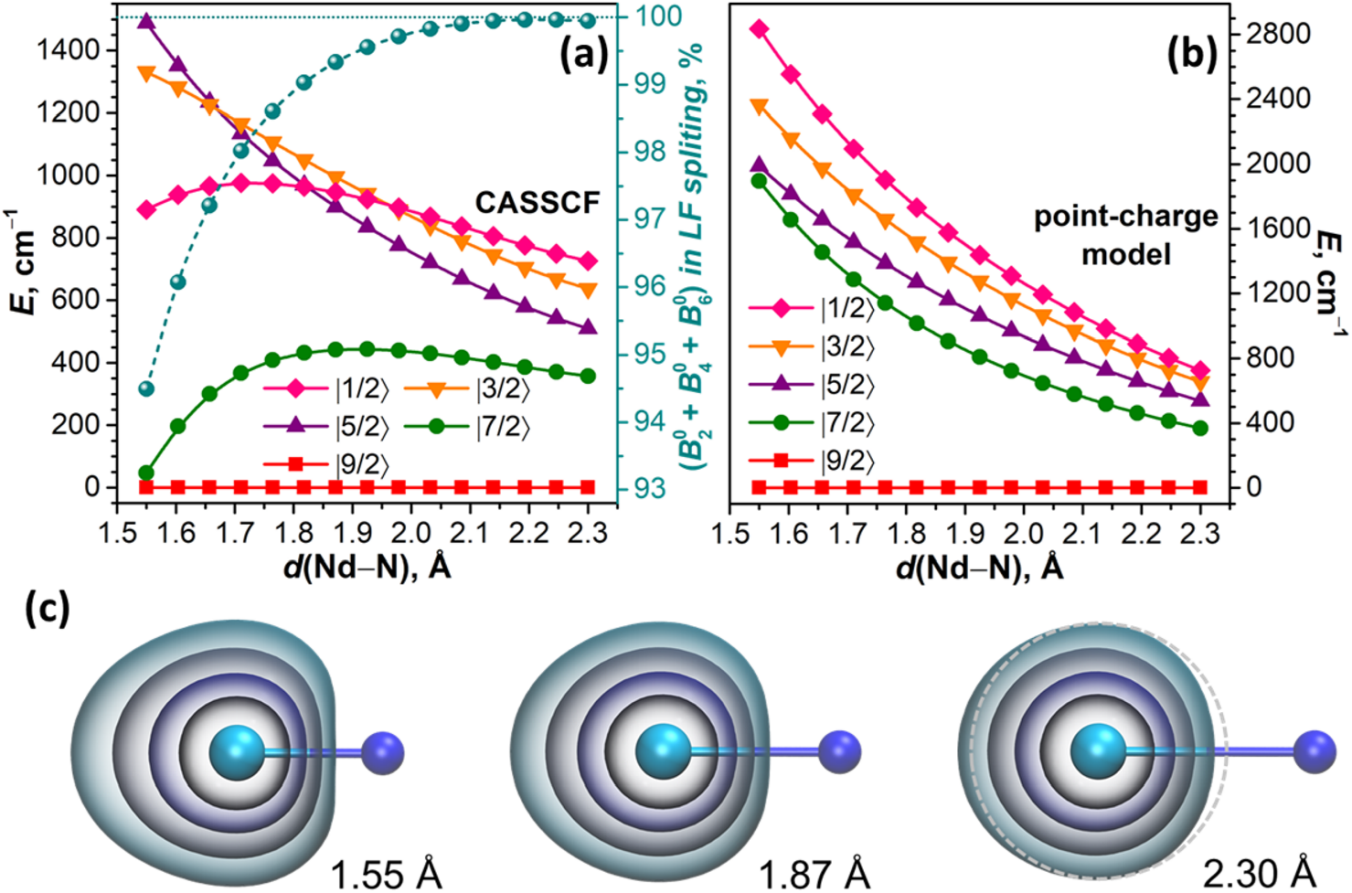
(a) LF splitting in the NdN molecule computed at the CASSCF/RASSI level as a function of the Nd–N distance and the sum of *B*_0_^2^, *B*_0_^4^, and *B*_0_^6^ contributions to the total LF splitting (cyan spheres with the dashed curve and the scale on the right side); (b) LF splitting in the NdN molecule computed with the point charge model and scaled LoProp atomic charge of N at the same Nd–N distances as in (a); (c) 4f-electron density in Nd–N built upon orbitals from CASSCF(3, 7) calculations, and concentric isosurfaces are plotted at the values of (from inner to outer) 0.25, 0.05, 0.01, and 0.002 a.u.; to guide the eye, the rightmost figure shows a circle centered on the Nd atom and plotted with the gray dashed line.

For the linear NdN molecule with the axial ligand field of *C*_∞v_ symmetry, the LF Hamiltonian should include only *B*_0_^2^, *B*_0_^4^, and *B*_0_^6^ terms, while LF states are described by pure |*m*_*J*_〉 functions. At a Nd–N distance of 2.3 Å, the *ab initio* computed LF splitting is 726 cm^−1^, and the state energies increase in the descending order of |*m*_*J*_| (Fig. 6a). The structure of states and their energies are reproduced closely by a point charge model using a LoProp charge of nitrogen (−1.513 *e*) scaled by a factor of 1.08 (Fig. 6b). The same factor was used then to scale the LoProp charges of nitrogen for point-charge computations at shorter Nd–N distances. According to the electrostatic model, a stepwise decrease of the Nd–N distance from 2.3 Å to 1.55 Å does not change the order of states and only gradually increases the LF splitting to 2836 cm^−1^, while the energies of all LF states increase monotonously (Fig. 6b). A very different Nd–N distance dependence emerges from CASSCF calculations. The energies of |±7/2〉 and |±1/2〉 doublets relative to |±9/2〉 pass through a maximum and then decrease, and |±3/2〉 also changes a curvature at short Nd–N, and only |±5/2〉 grows continuously in the whole range. The order of |*m*_*J*_〉 states thus changes several times, and the overall LF splitting at 1.55 Å is almost twice smaller than predicted by the point-charge model (Fig. 6a).

From the contributions of *ab initio* computed *B*_*q*_^*k*^ terms to LF splitting provided by the Single_aniso code,^122,127^ we infer that the weight of the *B*_0_^2^ term, which amounts to 87% at 2.3 Å, decreases to 49% at 1.55 Å, and the weight of *B*_0_^6^ increases from 7% at 2.3 Å to 38% at 1.55 Å, while the sign of *B*_0_^6^ changes from negative to positive below 2.1 Å (in the point-charge model, *B*_0_^6^ is negative through the whole range). Furthermore, the LF Hamiltonian, comprising only *B*_0_^2^, *B*_0_^4^, and *B*_0_^6^, cannot reproduce the LF splitting at short Nd–N distances, and requires addition of *B*_0_^8^. The weight of the latter increases from negligible 0.05% at 2.3 Å to significant 5.5% at 1.55 Å (Fig. 6a). As *B*_*q*_^*k*^ terms with *k* > 6 should vanish for pure f orbitals, their non-zero contribution serves as a diagnostic criterion for the breakdown of the assumption of the isolated spherically shaped 4f shell.^122^ Indeed, the shape of 4f-electron density distribution changes from nearly spherical at 2.3 Å to a considerably elongated one at short Nd–N distances (Fig. 6c). This naturally leads to the notion of 4f AOs mixing with other orbitals and the overall increase of the covalency as the Nd–N distance shrinks. Of course, the distance of 1.55 Å is unrealistically short, but it serves here only for a clear-cut demonstration, whereas the effect is already present at more realistic distances (Fig. 6c). Importantly, *ab initio* calculations of NdM_2_N@C_80_ similarly showed that the LF Hamiltonian including *B*_*q*_^*k*^ terms with *k* ≤ 6 recovers only ∼95% of the *ab initio* LF splitting, while covering the remaining 5% requires *B*_*q*_^8^ terms.

#### Nephelauxetic effect

The enhanced covalency of the Nd bonding with surrounding atoms in NdM_2_N@C_80_ has an experimental manifestation in the aforementioned red shift of ^4^F_3/2_ → ^4^I_*J*_ PL bands when compared to other Nd compounds. From the KD1 line of the ^4^F_3/2_ → ^4^I_9/2_ band in the low-temperature PL spectrum, the energy of the emitting state, KD1 of ^4^F_3/2_, is determined to be 10 900–11 000 cm^−1^, which is lower than the usual energy of the ^4^F_3/2_ multiplet by around 1100 cm^−1^. Such a covalency-induced reduction of the excitation energies in coordination compounds of transition metals and lanthanides is known as the nephelauxetic effect.^128^

Numerical representation of the nephelauxetic effect is often given by nephelauxetic parameters *β*_F_ = *F*^2^(compound)/*F*^2^(free ion), where *F*^2^ is the second-order Slater integral describing interelectron repulsion, or *β*′ = *ζ*_4f_/*ζ*_4f_(free ion), where *ζ*_4f_ is the Lande spin–orbit coupling constant. The degree of covalency, defined as an admixture of ligand orbitals to f-like orbitals, is sometimes estimated as *b*^0.5^ = [(1 − *β*)/2]^0.5^, although it appears somewhat ambiguous because *β*_F_ and *β*′ can be quite different, *F*^2^ being more sensitive to the bonding nature.^129,130^ For instance, the energy differences between ^4^I_*J*_ levels are mainly determined by *ζ*_4f_, and since the gaps between ^4^I_*J*_ levels in the NdM_2_N@C_80_ series are similar to other Nd compounds, we can conclude that *ζ*_4f_ is not strongly affected. The energy differences between multiplets with different *L*, on the other hand, are more sensitive to inter-electron repulsion parameters, and the reduced ^4^F_3/2_ energy in NdM_2_N@C_80_ indicates that *F*^2^ is significantly modified.

Experimentally, *ζ*_4f_, *F*^2^ and other *F*^*k*^ integrals can be determined by fitting the Hamiltonian, comprising free-ion and crystal-field terms, to spectroscopic data. As such a Hamiltonian includes a large number of parameters, extended data on many multiplets in a broad energy range are required for a reliable fitting. In the absence of such data, which is the case for NdM_2_N@C_80_ and the majority of Nd-SMMs, a simplified spectroscopic nephelauxetic parameter *β*_spec_ based on the transition energy between selected multiplets can be used: *β*_spec_ = Δ*E*(compound)/Δ*E*(standard). For the following discussion, we define *β*_spec_ as the energy difference between barycenters of ^4^I_9/2_ and ^4^F_3/2_ multiplets divided by the free ion value^75^ Δ*E*(^4^F_3/2_–^4^I_9/2_) = 11 699 cm^−1^. When the ^4^F_3/2_ → ^4^I_9/2_ PL band is not resolved into LF components, as is the case for many Nd-SMMs, PL band energy is simply used instead of barycenters, which can introduce an uncertainty to *β*_spec_ of ∼0.01.

**Fig. 7:**
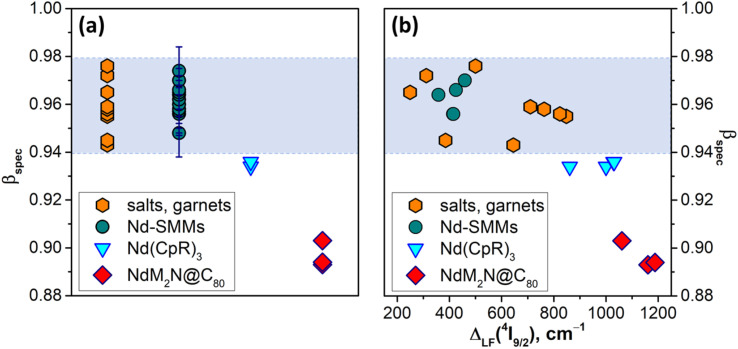
(a) Distribution of the spectroscopic nephelauxetic parameter *β*_spec_ in four groups of Nd compounds, including NdM_2_N@C_80_ molecules from this work, spectroscopically characterized Nd-SMMs, selected Nd-tris(η^5^-cyclopentadienyl) complexes Nd(CpR)_3_, and Nd^3+^ salts and garnets; (b) correlation between *β*_spec_ and *Δ*_LF_(^4^I_9/2_) for the same compounds. The range of typical *β*_spec_ values in Nd compounds is shaded. For many Nd-SMMs, *β*_spec_ is estimated from the unresolved ^4^F_3/2_ → ^4^I_9/2_ PL band instead of multiplet barycenters, which introduces an uncertainty of ∼0.01 plotted in (a). As *Δ*_LF_(^4^I_9/2_) is determined spectroscopically only for a small fraction of Nd-SMMs, the number of Nd-SMM datapoints in panel (b) is much smaller than in panel (a).

Fig. 7a plots *β*_spec_ values in NdM_2_N@C_80_, all 17 PL-characterized Nd-SMMs reported before this work,^30–33,36,81–85,112,131,132^ selected tris(η^5^-cyclopentadienyl)Nd complexes with large ligand field splitting,^116–118^ and several inorganic salts and garnets thoroughly characterized by optical spectroscopy^133–139^ (see Table S9† for the compound titles, numerical values, and literature references for each entry). The latter group is the most studied experimentally, and for this analysis we selected the set of compounds encompassing the broadest range of *β*_spec_ parameters, from the most ionic ones like Nd:LaF_3_ (ref. 139) with *β*_spec_ = 0.976 to the most covalent Nd:Y_2_O_3_ (ref. 133) and Nd_2_S_3_ (ref. 134) with *β*_spec_ of 0.943–0.945. A similar span of *β*_spec_ from 0.948 to 0.982 is found for Nd-SMMs. It appears safe to state that the *β*_spec_ range of 0.94–0.98 is where the vast majority of Nd compounds will be found. Amberger *et al.* demonstrated that Nd(CpR)_3_ complexes (CpR = C_5_Me_5_, C_5_Me_4_H, C_5_H_4_^*t*^Bu, C_5_H_4_SiMe_3_, *etc.*) with enhanced metal–ligand interactions have large LF splitting and reduced nephelauxetic parameters *β*_F_ (see ref. 116 for comparison of LF strength and *β*_F_ in a series of Nd complexes).^116–118^ Likewise, their *β*_spec_ values of 0.934–0.936 fall below the normal *β*_spec_ range and were presumably the lowest values among Nd-compounds before our study. Analyzed in this broad context, the *β*_spec_ parameters found in this work for the NdM_2_N@C_80_ series, 0.893 for NdLu_2_N@C_80_, 0.894 for NdY_2_N@C_80_, and 0.903 for NdSc_2_N@C_80_, are unprecedently small. This very strong nephelauxetic effect evidences one more time that by increasing the internal strain in metallofullerenes we achieved a much higher degree of covalency than is usually observed in Nd compounds. It is also instructive to find that there is seemingly no correlation between the nephelauxetic effect and ligand field splitting for the compounds in the normal *β*_spec_ range (Fig. 7b). However, as we leave this range, the decrease of *β*_spec_ starts to correlate with the increase of *Δ*_LF_. This is not surprising given that the strengthening of the ligand field is achieved by enhancing the interactions between the lanthanide ion and the ligands, but it will require more compounds with such unusual properties to establish firm correlations.

The strong nephelauxetic effect and ligand field splitting may also be among the reasons for the short luminescence lifetimes of NdM_2_N@C_80_. The low energy of the NIR-emitting state in lanthanide compounds is responsible for the efficient nonradiative vibrational relaxation. For Nd^3+^, the non-radiative relaxation rate is determined by the gap between ^4^F_3/2_ and ^4^I_15/2_, which typically is around 5400 cm^−1^. The ^4^I_15/2_ multiplet of NdM_2_N@C_80_ is not directly accessible in our experiments, but based on the experimental and computational data on other ^4^I_*J*_ multiplets (Table S4†), we can assume that the energy of ^4^I_15/2_ is also close to standard values for Nd^3+^ and that the LF splitting of the ^4^I_15/2_ multiplet amounts to *Δ*_LF_ ≈ 1300 cm^−1^ (Table S4†). The lower than usual energy of the ^4^F_3/2_ state and the strong LF splitting of the ^4^I_15/2_ multiplet reduce the gap between the KD1 of ^4^F_3/2_ and KD8 of ^4^I_15/2_ to ≈3600 cm^−1^. This low gap opens the possibility for two-phonon relaxation and thus, in accordance with the energy gap law, is a plausible reason for the anomalously short Nd^3+^ luminescence lifetimes in NdM_2_N@C_80_ molecules.

### Magnetic properties of NdSc_2_N@C_80_

While the energies of LF states are accessible *via* PL spectroscopy, experimental verification of the state compositions is not straightforwardly available from this technique. Complimentary information on the ground state may be given by magnetometry. The large *Δ*_1,2_ value in the ^4^I_9/2_ multiplet suggests that the low-temperature magnetic properties of NdM_2_N@C_80_ will be dominated by the ground state doublet.

#### SQUID magnetometry

**Fig. 8:**
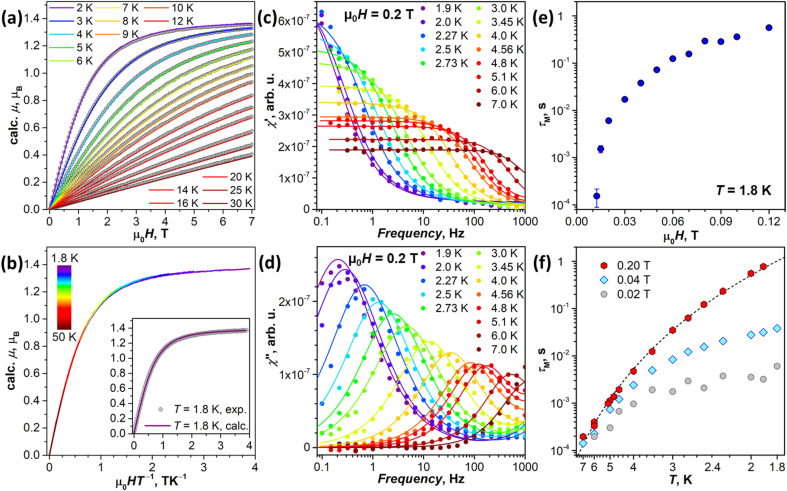
(a) Experimental magnetization curves of NdSc_2_N@C_80_ (gray dots) compared to calculations (colored lines) for pseudospin *s*˜ = 1/2 with the *ab initio* computed g-tensor corresponding to the ground-state Kramers doublet (KD1, Table 1). (b) Magnetization curves of NdSc_2_N@C_80_ measured between 1.8 K and 50 K and presented as a function of the HT^−1^ product; the inset shows the comparison of experimental and calculated data for *T* = 1.8 K. (c) and (d) AC magnetometry measurements of in-phase *χ*′ (c) and out-of-phase *χ*′′ (d) magnetic susceptibility of NdSc_2_N@C_80_ in the constant field of 0.2 T at *T* = 1.9–7 K; dots are experimental data, and lines are fits with the generalized Debye model, from which relaxation times are extracted. (e) Magnetic field dependence of magnetization relaxation time at *T* = 1.8 K. (f) Temperature dependencies of magnetization relaxation times measured in the field of 0.02 T, 0.04 T, and 0.2 T; dashed line is a fit of 0.2 T data with the function *τ*_M_^−1^ = CT^*n*^ (*C* = 0.0205 and *n* = 6.59).

The experimental magnetic properties of NdSc_2_N@C_80_ powder were first studied by SQUID magnetometry (Fig. 8a and b). The magnetization curves of the sample measured below 30 K are compared to simulations for the pseudospin with the *ab initio* computed g-tensor for KD1 in Sc-3 in Fig. 8a. A very good match between the shapes of the experimental and calculated curves points to the reliability of the theoretical predictions for the ground state KD. Furthermore, the *M versus* HT^−1^ plots superimpose at least up to 50 K (Fig. 8b). It shows that only the ground state KD contributes to the magnetization at these temperatures, in line with the large predicted energy gap between the first and second KDs (Table 1).

#### XMCD

**Fig. 9:**
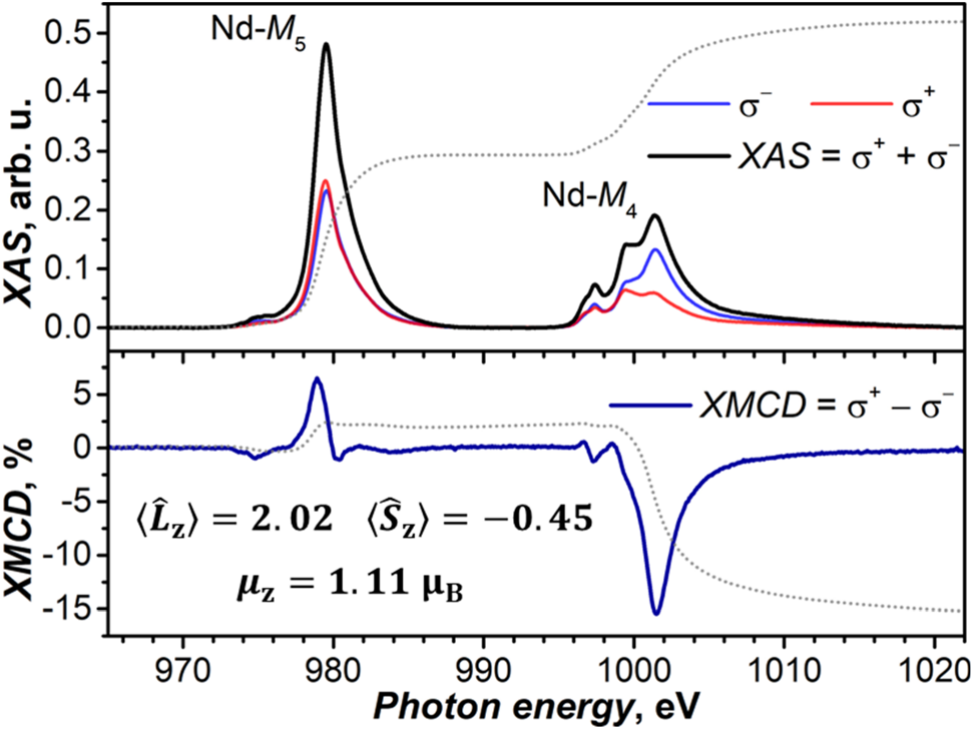
XAS (upper panel) and XMCD (lower panel) spectra of NdSc_2_N@C_80_ at the Nd-*M*_4,5_ edges (3d → 4f), *T* ≈ 5.5 K, and *μ*_0_*H* = 6 T. Left-hand and right-hand polarized XAS spectra are denoted as σ^+^ and σ^−^, and non-polarized XAS spectrum is their sum, while XMCD (in %) is their difference normalized to the maximum of XAS. Dotted lines are integrals of XAS and XMCD used in the sum rule analysis, which provided 〈*L̂*_z_〉, 〈*Ŝ*_z_〉, and *μ*_z_ values (see the ESI for details†). Measurements were performed at the BOREAS beamline (synchrotron ALBA).^140^

The determination of the absolute value of the magnetic moment of NdSc_2_N@C_80_ by SQUID measurements is not sufficiently reliable because of the limited sample amount. Complimentary information on the ground state magnetic properties of NdSc_2_N@C_80_ was thus obtained from X-ray magnetic circular dichroism (XMCD) measurements performed on the sample, which was drop-casted onto a HOPG substrate. In the absence of a magnetic field, the X-ray absorption spectrum of NdSc_2_N@C_80_ has no dichroism and shows the typical absorption features of Nd(iii) at the Nd-M_4,5_ edges (3d → 4f excitations) (Fig. 9 and S20†). When a magnetic field is applied, circular polarized XAS develops a dichroism proportional to the sample magnetization in the direction of the beam (which was kept parallel to the field in our measurements) (Fig. 9). The maximum of the XMCD signal at the Nd-M_5_ edge at a photon energy of 1001.5 eV was used to follow the magnetization of NdSc_2_N@C_80_ during magnetic field ramps between −6 T and +6 T. The magnetization curves measured this way reproduced the results of the SQUID measurements at 5.5 K closely (see Fig. S21 in the ESI†). By applying sum rules^141,142^ and tabulated correction factors,^143^ the XAS and XMCD spectra recorded in the field of 6 T were used to calculate the expectation values of angular and spin momentum operators, 〈*L̂*_z_〉 = 2.02 μ_B_ and 〈*Ŝ*_z_〉 = −0.45 μ_B_, and the magnetic moment along the beam direction, *μ*_z_ = 1.11 μ_B_ (see the ESI for the calculation details†). The uncertainty in the determined value of *μ*_z_ is mainly caused by ambiguities in integration limits for the Nd-M_4_ edge and is estimated to be less than ±0.1 μ_B_. The ratio 〈*Ŝ*_z_〉/〈*L̂*_z_〉 = −0.22 is close to the *ab initio* prediction of −0.24 for the ground state doublet (note that a theoretical expectation for the f^3^ system is −0.25). For comparison, in the assumption of a disordered powder, the *ab initio* predicted ground-state g-tensor of NdSc_2_N@C_80_ gives a saturated magnetic moment of 1.38 μ_B_. At *T* = 5.5 K and under an applied magnetic field of 6 T, the theoretical moment is reduced to 1.14 μ_B_, which is very close to the value we determined from XMCD measurements. At the same time, the ground state with the pure *m*_*J*_ = ±9/2 composition would have a saturated magnetic moment of 1.65 μ_B_, while the value at 5.5 K and 6 T would be 1.43 μ_B_. Thus, XMCD measurements confirm the *ab initio* prediction of the considerable deviation of the NdSc_2_N@C_80_ ground state KD from the pure *m*_*J*_ = ±9/2 state.

#### ESR spectroscopy

Precise information on the ground-state g-tensor can in principle be obtained by ESR spectroscopy. We therefore performed X-band ESR measurements of frozen NdSc_2_N@C_80_ solution in glassy *d*_8_-toluene. However, the sample did not show any discernible ESR signal at 10 K or higher temperature. Note that with *m*_*J*_ = ±9/2 being the main components of KD1, the ground-state wavefunction requires an admixture of *m*_*J*_ = ±7/2 terms to fulfill the ESR selection rule, Δ*m*_*J*_ = ±1. As can be seen in Table 1, the contribution of *m*_*J*_ = ±7/2 terms to KD1 is very small (less than 1%), suggesting that the absence of the ESR signal can be caused by negligible transition matrix elements.

#### Dynamic magnetic properties

NdSc_2_N@C_80_ did not show hysteresis in DC magnetic measurements, and the sample was further studied by AC magnetometry (Fig. 8c–f and S22†). The relaxation of magnetization was too fast in zero magnetic field, and no peaks of the out-of-phase χ′′ signal were recorded. The slow relaxation at 1.8 K could be detected only in the external fields exceeding 0.015 T (Fig. S22†). The relaxation time *τ*_M_ gradually increased with the field and approached tenths of a second near 0.1 T, with a sign of levelling-off at higher fields (Fig. 8e and Table S10†). The fast relaxation in zero field is caused by the quantum tunneling of magnetization (QTM) as the ground state g-tensor has rather large transversal components *g*_x,y_ (Table 1). When the constant magnetic field is applied, the QTM is gradually suppressed, resulting in the increase of the relaxation time.

The temperature dependence of *τ*_M_ was measured in the fields of 0.02, 0.04, and 0.2 T (Table S11†). The QTM is fully suppressed at 0.2 T, and the temperature dependence of *τ*_M_ can be well described by the Raman relaxation mechanism, *τ*_M_^−1^ = CT^*n*^, with the fitted parameters *C* = 0.0205 ± 0.0026 s^−1^ K^−*n*^ and *n* = 6.59 ± 0.09 (Fig. 8f). The contribution of the QTM, inducing faster relaxation with flatter temperature dependence at lower temperatures, is clearly visible in smaller fields (Fig. 8f). Higher-energy LF states are thus not involved in the reorientation of NdSc_2_N@C_80_ magnetic moment as the system finds an underbarrier shortcut with the help of phonon excitations.

The relaxation behavior observed in this work for NdSc_2_N@C_80_ can be compared to that of non-fullerene Nd-SMMs. As discussed above, Nd-SMMs usually have considerable *g*_x,y_ components in the ground state, which lead to fast zero-field QTM. Only three Nd complexes, two of [Nd(H_2_O)_5_L_2_]^3+^ type with pentagonal bipyramidal coordination geometry (L = ^*t*^BuPO(NH^*i*^Pr)_2_ in ref. 125 and CyPh_2_PO in ref. 126), and the (COT)Nd(Cp^ttt^) sandwich diluted with the Y analog,^115^ exhibited slow relaxation of magnetization in zero magnetic field. In all other Nd-SMMs, the slow relaxation required the DC magnetic field to suppress the fast QTM. [Nd(H_2_O)_5_(CyPh_2_PO)_2_]^3+^ and (COT)Nd(Cp^ttt^) complexes are also the only Nd-SMMs, for which a narrow magnetic hysteresis could be recorded at 2 K.^115,126^ For other Nd-SMMs, the slow relaxation was attested only by AC measurements, implying relaxation times shorter than 1 s. The temperature dependence of relaxation times for some Nd-SMMs was described by the Raman mechanism with an exponent of *n* = 6–7,^100,114,126,144–146^ similar to the value of *n* = 6.59 determined in this work for NdSc_2_N@C_80_. When instead the Orbach mechanism was assumed to describe the relaxation of Nd-SMMs, the barriers obtained from the fits of the temperature dependence were usually several times smaller than the energies of excited LF states predicted *ab initio*.^113,114,125,126,144–146^ This inconsistency is a strong indication that the Orbach mechanism is rarely operative for Nd-SMMs, while relaxation of magnetization is more often governed by the Raman mechanism, which may also involve coupling to optical phonons.

## Conclusions

The synthesis of a NdM_2_N@C_80_ series with diamagnetic rare-earth metals of different sizes (M = Sc, Y, Lu) allowed us to analyze the influence of internal strain on the magnetic states and optical properties of Nd^3+^ in these metallofullerenes. We found that Nd^3+^ ions in nitride clusterfullerenes experience the strongest ligand field ever observed in molecular complexes of Nd, causing the LF splitting of the ground-state ^4^I_9/2_ multiplet reaching 1100–1200 cm^−1^. This strong ligand field is the result of the unusually short Nd–N bond lengths and the large negative charge of the nitride ion. At the same time, a confinement of the NdM_2_N cluster of a variable size inside the fullerene cage results in the increase of the strain from Sc to Lu to Y, which leads to the change of the geometrical shape of the cluster and substantial variation of the LF splitting pattern of the Nd^3+^ ion. The latter could be directly addressed by photoluminescence spectroscopy, which showed finely structured narrow emission lines caused by f–f transitions in Nd^3+^. When compared to their energies in Nd^3+^ compounds, the PL bands of NdM_2_N@C_80_ are considerably red-shifted, which points to the very strong nephelauxetic effect and enhanced contribution of covalency in Nd bonding.

The enhanced covalency is the price for the large LF splitting and, as shown by *ab initio* modelling, is the reason for the reduced magnetic axiality in NdM_2_N@C_80_. Point-charge modelling demonstrates that pure electrostatic interactions in these molecules would produce highly axial LF states with sheer *m*_*J*_ wavefunction compositions. Experimental studies of magnetic properties revealed that NdSc_2_N@C_80_ exhibits slow relaxation of magnetization only in the presence of an external magnetic field, whereas its zero-field relaxation is dominated by the fast tunneling process. The large LF splitting also does not help to slow down the in-field relaxation of magnetization, which appears to be governed by the under-barrier Raman mechanism.

The results of this work demonstrate that pushing the LF strength to its extreme by enhancing interactions of Nd ions with surrounding atoms does not improve the magnetic axiality even in the molecular geometry, which favors such axiality for heavy lanthanides. The reason is that the larger ionic radius makes Nd more susceptible to enhanced covalency. Theoretically, the single-ion magnetic anisotropy of Nd^3+^ could be made highly axial by placing point charges at short distances, but in reality, the strong LF would require an interaction with strong ligands placed at close distances, which inevitably leads to the enhanced covalency and all its negative effects on the magnetic axiality discussed in this work. This dichotomy between the strong ligand field and reduced magnetic axiality appears to be a fundamental limitation for creating high-performance SMMs based on light lanthanides. Nevertheless, the large LF splitting appears very instrumental in separating f–f transitions and enabling direct optical access to individual spin states of Nd^3+^, which can be useful for application in magneto-photonic quantum technologies. Another possible consequence of enhanced covalency worth mentioning is that the 4f-shell can become more susceptible to manipulations by other methods like scanning tunneling microscopy, which is usually considered not amenable to probe 4f electrons in molecules due to their core-like behavior. Thus, while Nd-EMFs may not perform great as single molecular magnets, they show good prospects for optically and electronically addressable quantum bits. Importantly, their LF splitting and spin-state wavefunction can be further tuned by varying the composition of the endohedral cluster or exohedral chemical modification of metallofullerenes.

## Data availability

The data supporting the findings of this study are available from the corresponding authors upon reasonable request.

## Author contributions

WY synthesized fullerenes with help and supervision from FL; MR and FZ performed PL measurements; VD performed DFT calculations under the supervision of SMA; GV and MB performed SQUID measurements; SS performed vibrational spectroscopic studies; MR, GV, MB, LS, CG, MV and AAP performed the XMCD study; SMA performed *ab initio* calculations; FL grew single-crystals and performed the SC-XRD study; AAP conceived and supervised the study; FL, SMA, and AAP wrote the manuscript with contributions from all co-authors.

## Conflicts of interest

There are no conflicts to declare.

## Supplementary Material

SC-015-D3SC05146C-s001

SC-015-D3SC05146C-s002
