# Supplementary material for: Covalency versus magnetic axiality in Nd molecular magnets: Nd-photoluminescence, strong ligand-field, and unprecedented nephelauxetic effect in fullerenes NdM2N@C80 (M = Sc, Lu, Y)
Source: Chem Sci. 2023 Dec 21;15(6):2141–57. doi: 10.1039/d3sc05146c (PMC10848757; doi:10.1039/d3sc05146c)
Supplement: SC-015-D3SC05146C-s001 [file SC-015-D3SC05146C-s001.pdf]

Electronic Supplementary Material (ESI) for Chemical Science.
This journal is © The Royal Society of Chemistry 2023

Covalency *versus* magnetic axiality in Nd molecular magnets: Nd-photoluminescence, strong ligand-field, and unprecedented nephelauxetic effect in fullerenes $NdM_2N@C_{80}$ (M = Sc, Lu, Y)

Wei Yang,[a] Marco Rosenkranz,[a] Georgios Velkos,[a] Frank Ziegs,[a] Vasili Dubrovin,[a] Sandra Schiemenz,[a] Lukas Spree,[a,b] Matheus Felipe de Souza Barbosa,[a] Charles Guillemard,[c] Manuel Valvidares,[c] Bernd Büchner,[a] Fupin Liu,*[a] Stanislav M. Avdoshenko,*[a] Alexey A. Popov*[a]

Supporting Information

Experimental details S2

Synthesis S4

Spectroscopic properties S7

Single-crystal XRD analysis S9

DFT calculations S11

Photoluminescence decay S14

Photoluminescence spectra S15

Photoluminescence -derived LF splitting S21

CASSCF and point charge calculations of $NdSc_2N@C_{80}$ S22

CASSCF calculations of $Nd(C_5Me_5)_3$ and $Nd(C_5Me_4H)_3$ S27

CASSCF calculations of $NdLu_2N@C_{80}$ and $NdY_2N@C_{80}$ S28

Spectroscopic nephelauxetic parameters in Nd-compound S31

XMCD measurements S32

Magnetization relaxation times S34

References S36

Experimental details

HPLC: HPLC analysis and separation were performed for toluene solutions of fullerene and with toluene as an eluent, employing semipreparative COSMOSIL Buckyprep and Buckyprep-D chromatographic columns (Nacalai Tesque) and Agilent 1260 Infinity II LC System. Recycling HPLC separation was performed using Sunflow 100 system (SunChrome).

Mass spectrometry: Laser desorption/ionization time-of-flight (LDI-TOF) mass-spectra were measured with a Bruker autoflex mass-spectrometer.

UV-Vis spectrometry: UV-vis-NIR absorption spectra were measured in toluene solution at room temperature with Shimadzu 3100 spectrophotometer.

Vibrational spectroscopy. Raman measurements were performed with T64000 spectrometer (Horiba). The sample was drop-casted from toluene solution onto KBr substrate and cooled down to 78 K. The spectra were measured with laser excitations at 532 nm and 660 nm (Torus lasers by Laser Quantum). For the measurement with 785 nm excitation (BrixX diode laser by Omicron Laserage), the sample was drop-casted on gold SERS substrate (Metrohm DropSens DRP-C220BT).

X-ray diffraction. Single crystal X-ray diffraction data collection was carried out at 100 K at the BESSY storage ring (BL14.2, Berlin-Adlershof, Germany).[1] XDSAPP2.0 suite was employed for data processing.[2, 3] The structure was solved by direct methods and refined by SHELXL-2018.[4] Hydrogen atoms were added geometrically and refined with a riding model.

DFT computations. DFT optimization of $NdM_2N@C_{80}$ molecules was performed at the PBE/PAW level using the VASP code and recommended pseudopotentials with f-shell in-core treatment.[5-9]

CASSCF calculations. *Ab initio* energies and wave functions of Nd^{3+} LF multiplets in $NdM_2N@C_{80}$ molecules have been calculated at the CASSCF/SO-RASSI level of theory using the quantum chemistry package OpenMOLCAS[10] and SINGLE_ANISO module.[11] The basis sets were ANO-RCC-VTZP or Nd and ANO-RCC-VDZP for other elements. Molecular structures used in CASSCF calculation were optimized with DFT.

Magnetic measurements. DC and AC magnetic measurements of powder samples were performed using a Quantum Design VSM MPMS3 magnetometer. Magnetic simulations were performed using PHI code.[12]

XMCD measurements. X-ray absorption measurements with circular polarized light were performed at beamline BOREAS[13] (synchrotron ALBA, Spain) using the total electron yield detection mode. Magnetic field was always oriented parallel to the X-ray beam. The sample was drop-casted onto HOPG substrate, which was glued to the sample-holder with a silver paste. To consider the variation of the X-ray beam intensity, the current from the sample was referred to the current from the gold grid. The measured spectrum was normalized to the intensity of the pre-edge signal, and then the broad background was subtracted. As a result of this procedure, the XAS signal intensity reported in the paper corresponds to the normalized increase of the XAS due to the specific absorption at the Nd-$M_{4,5}$ edge over the background signal. Non-polarized XAS intensity is defined as XAS = ($\sigma^+ + \sigma^-$), XMCD intensity is ($\sigma^- - \sigma^+$). When XMCD intensity is plotted in %, the XMCD signal is divided by the XAS intensity at its maximum. In the measurement of magnetization curves, σ^+ or σ^- intensity during magnetic field sweep was measured at fixed energies corresponding to the maximum of XMCD signal.

Photoluminescence spectroscopy. CW NIR-PL spectra were measured with home-made microscope using excitation at 488 nm from Omicron PhoxX diode laser and detected with Andor iDus 1.7 µm InGaAs camera attached to Kymera 328i spectrograph (Andor); temperature was controlled with Janis ST-500 microscopy cryostat. Good signal-to-noise ratio in PL spectra was obtained for acquisition times of 2–3 minutes. The

spectra did not show any changes during repeated measurements, indicating high photo-stability of the fullerene samples.

The PL decay measurements were performed with TCSPC technique based on ID900 Time Controller (ID Quantique). The 488 nm Omicron PhoxX diode laser was digitally modulated with the pulse width of 1 ns, and the ID230 NIR single-photon counter (ID Quantique) was used for broad-band time-resolved detection in the NIR range.

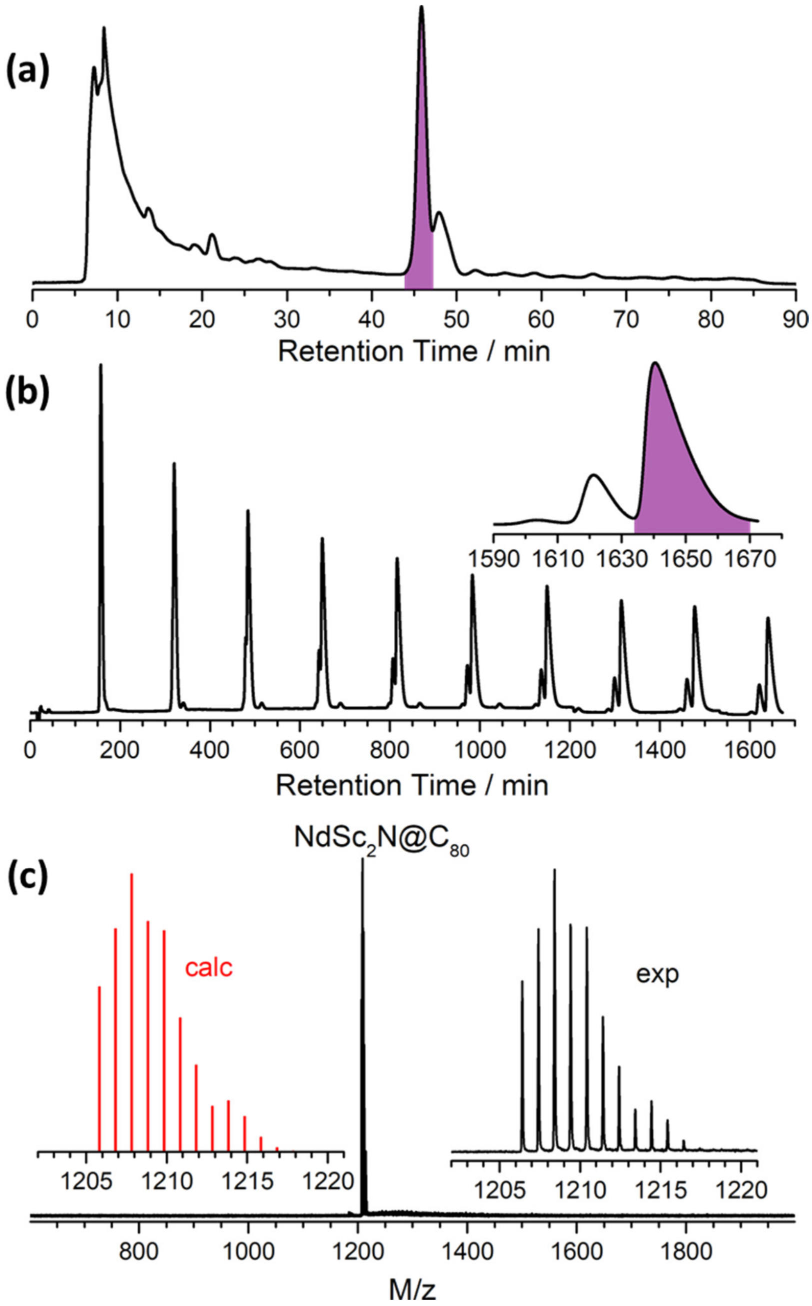

Figure S1. (a) HPLC chromatogram of crude CS_2 fullerene extract from Nd-Sc nitride clusterfullerene synthesis, $NdSc_2N@C_{80}$ is found in the highlighted fraction (2×Buckyprep columns, 5 mL/min). (b) Recycling HPLC chromatogram of the highlighted fraction collected in the first step, the inset shows the 10th cycle, the peak of $NdSc_2N@C_{80}$ is highlighted in purple (Buckyprep column, 1 mL/min). (c) Positive-ion LDI mass spectrum of isolated $NdSc_2N@C_{80}$; the insets show theoretical and experimental isotope distributions

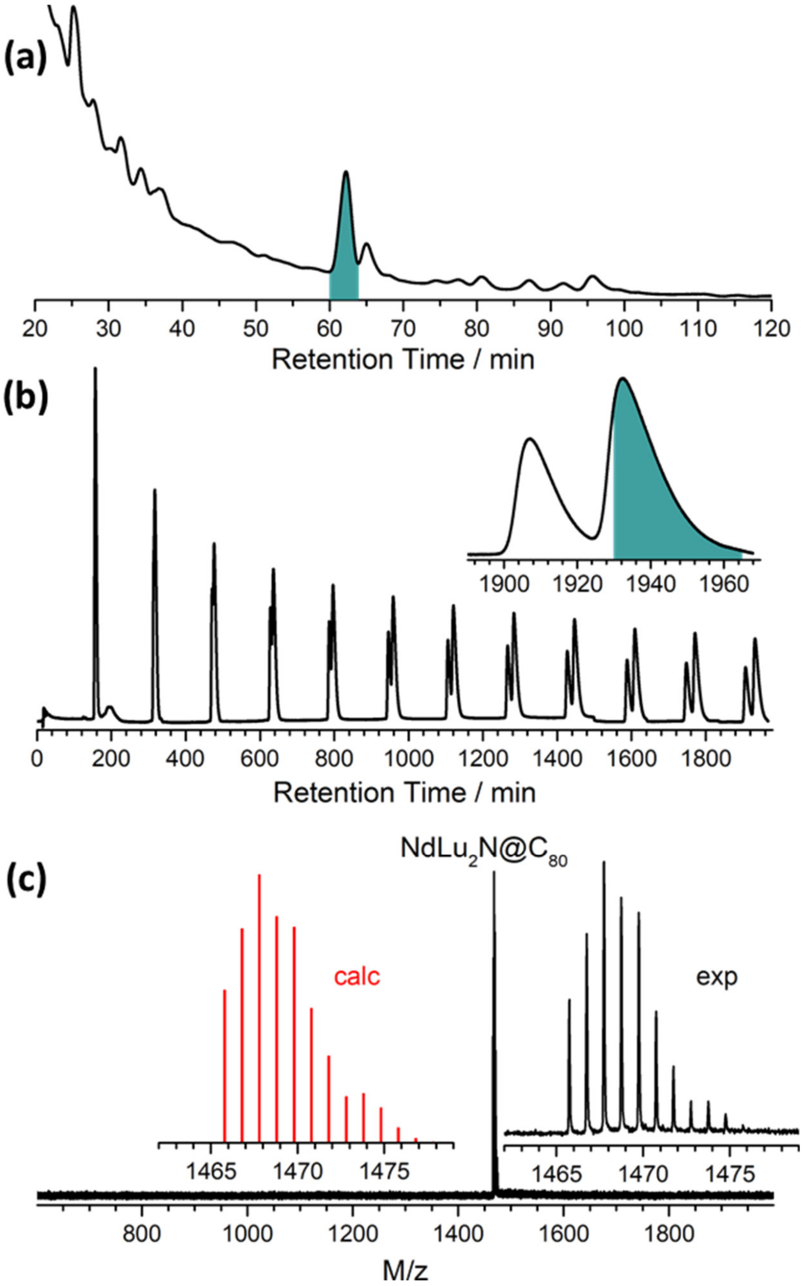

Figure S2. (a) HPLC chromatogram of crude CS_2 fullerene extract from Nd-Lu nitride clusterfullerene synthesis, $NdLu_2N@C_{80}$ is found in the highlighted fraction (2×Buckyprep columns, 4 mL/min). (b) Recycling HPLC chromatogram of the highlighted fraction collected in the first step, the inset shows the 12th cycle, the peak of $NdLu_2N@C_{80}$ is highlighted in dark cyan (Buckyprep column, 1 mL/min). (c) Positive-ion LDI mass spectrum of isolated $NdLu_2N@C_{80}$; the insets show theoretical and experimental isotope distributions

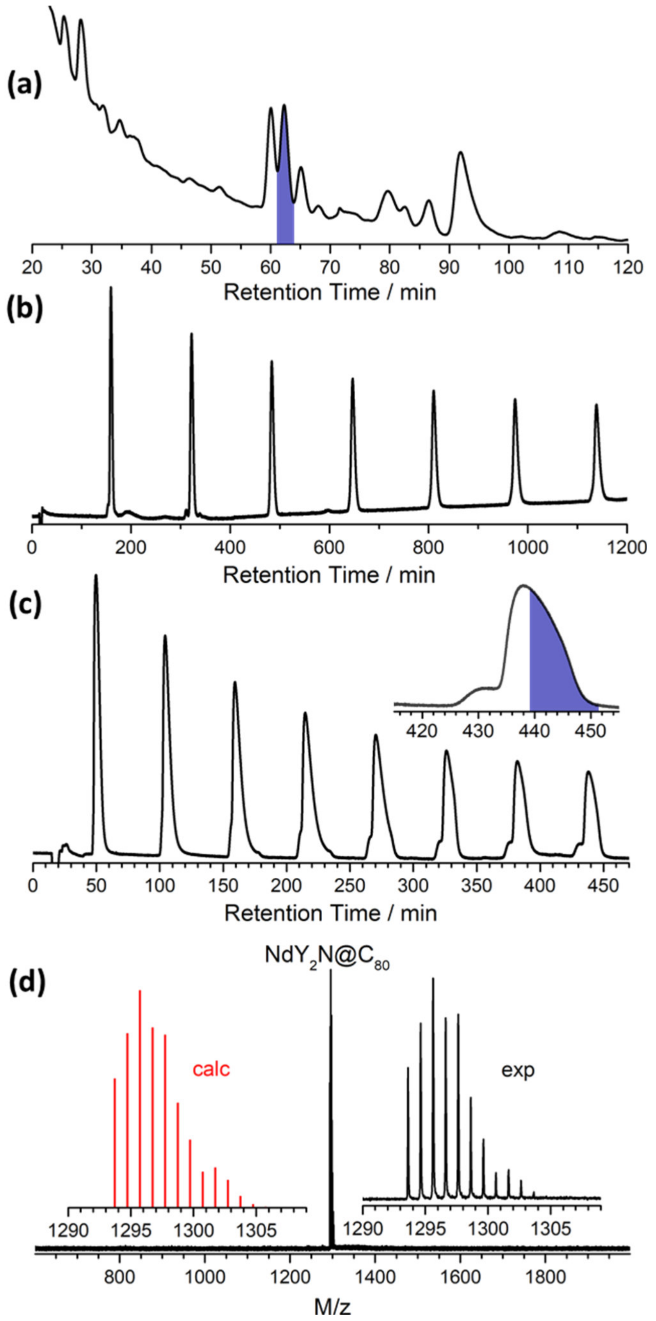

Figure S3. (a) HPLC chromatogram of crude CS_2 fullerene extract from Nd-Y nitride clusterfullerene synthesis, $NdY_2N@C_{80}$ is found in the highlighted fraction (2×Buckyprep columns, 4 mL/min). (b) Recycling HPLC chromatogram (Buckyprep column, 1 mL/min) of the highlighted fraction collected in the first step, only small traces of $Y_3N@C_{86}$ could be removed with this column, while the main fraction is a mixture of $NdY_2N@C_{80}$ and $Y_3N@C_{80}$. (c) Recycling HPLC chromatogram with Buckyprep-D column (1 mL/min), the inset shows 8th cycle, the part of peak with pure $NdY_2N@C_{80}$ is highlighted in blue. (d) Positive-ion LDI mass spectrum of isolated $NdY_2N@C_{80}$; the insets show theoretical and experimental isotope distributions.

UV-Vis absorption spectra

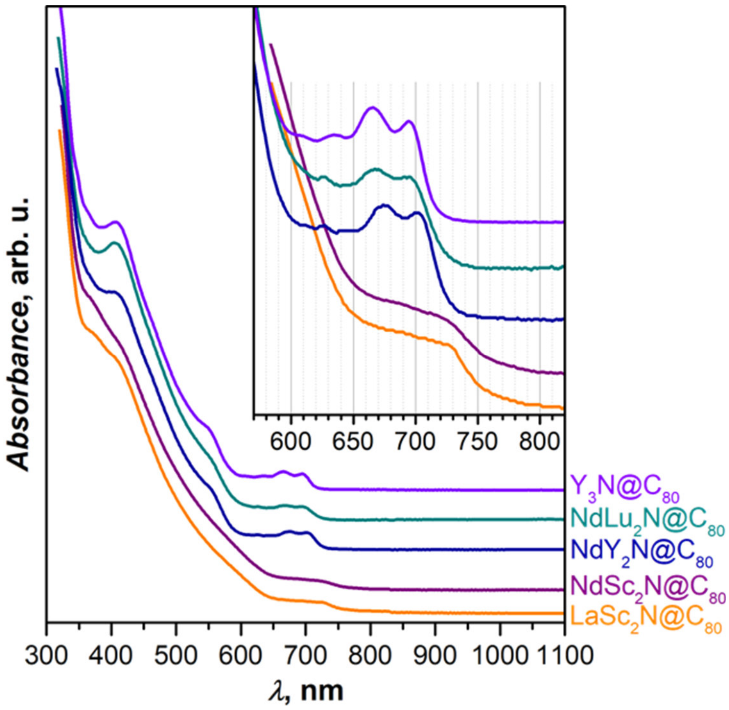

Figure S4. UV-Vis absorption spectra (room temperature, toluene solution) of isolated $NdM_2N@C_{80}$ (M = Sc, Lu, Y) compared to the spectra of $LaSc_2N@I_h$-C_{80} and $Y_3N@I_h$-C_{80}. The inset shows magnification of the low-energy part of the spectra.

When $M_3N@I_h$-C_{80} contains no Sc, the low-energy part of the absorption spectrum has several well-defined absorption features, with the lowest one near 700 nm. $Y_3N@I_h$-C_{80} can serve as a typical example, and the spectra of $NdLu_2N@C_{80}$ and $NdY_2N@C_{80}$ are very similar to that. The energy is only slightly affected by the size of the endohedral cluster (observe ≈5 nm shift between $NdY_2N@C_{80}$ and $Y_3N@C_{80}$). When Sc is present in the cluster, the bands usually become broader, and the lowest-energy absorption shows a red shift to 740 nm. Here $LaSc_2N@C_{80}$ is a typical example, to which the spectrum of $NdSc_2N@C_{80}$ appears very similar. None of $NdM_2N@C_{80}$ fullerenes shows additional absorption features which might be ascribed to f-f transitions of Nd^{3+}.

Vibrational spectra of $NdSc_2N@C_{80}$

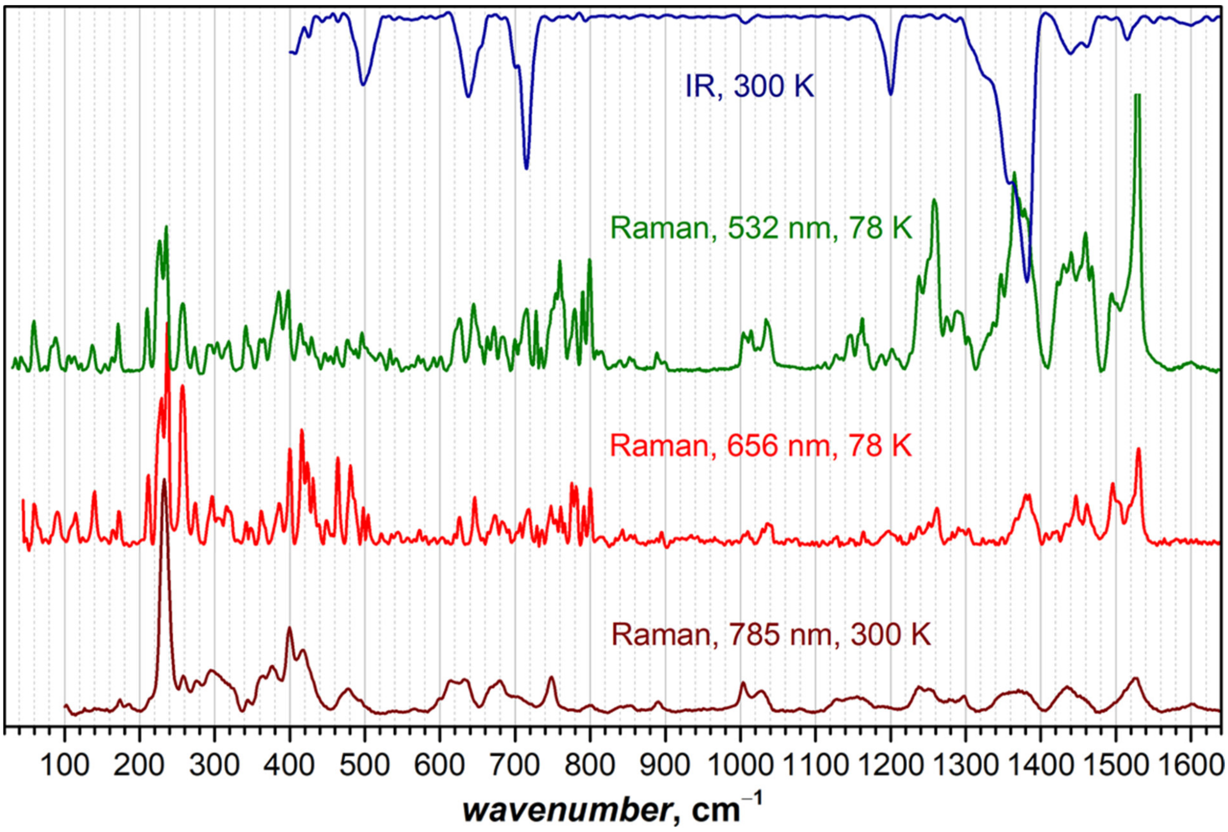

Figure S5. FTIR (dark blue trace) and Raman (red and green traces) spectra of $NdSc_2N@C_{80}$ drop-casted on KBr substrate. Raman spectra were excited at 532 and 660 nm and measured at 78 K. Brown trace is the Raman spectrum of $NdSc_2N@C_{80}$ drop-casted on gold SERS substrate and measured at room temperature with the excitation at 785 nm.

Single-crystal X-ray diffraction

Molecular structure of $NdSc_2N@I_h-C_{80}$ was established by single-crystal X-ray diffraction using a co-crystal with nickel(II) octaethylporphyrin (NiOEP) grown by layering solutions of fullerene in benzene and NiOEP in benzene and allowing slow diffusion thereof for one month. Measurements were performed at 100 K with synchrotron irradiation at the BESSY storage ring (BL14.2, Berlin-Adlershof, Germany).[1] XDSAPP2.0 suite was employed for data processing.[2, 3] The structure was solved by direct methods and refined by SHELXL-2018.[4]

Table S1. Crystal data.

**Crystal**	**$NdSc_2N@C_{80} \cdot NiOEP \cdot 2C_6H_6$**
**Formula**	$C_{128}H_{56}N_5NdNiSc_2$
**Formula weight**	1956.64
**Color, habit**	Black, block
**Crystal system**	monoclinic
**Space group**	*C*2/*c*
***a*, Å**	25.300(5)
***b*, Å**	14.980(3)
***c*, Å**	39.470(8)
***α*, deg**	90
***β*, deg**	95.44(3)
***γ*, deg**	90
**Volume, Å^3**	14892(5)
Z	8
***T*, K**	100
**Radiation (λ, Å)**	Synchrotron Radiation (0.82656)
**Unique data (R_{int})**	19096 (0.0409)
**Parameters**	1242
**Restraints**	0
**Observed data ($I > 2\sigma(I)$)**	18607
**R_1^a (observed data)**	0.0457
**wR_2^b (all data)**	0.1360
**CCDC No.**	2194854

^{a}For data with $I > 2\sigma(I)$, $R_1 = \frac{\sum||F_o|-|F_c||}{\sum|F_o|}$. ^{b}For all data, $wR_2 = \sqrt{\frac{\sum[w(F_o^2-F_c^2)^2]}{\sum[w(F_o^2)^2]}}$.

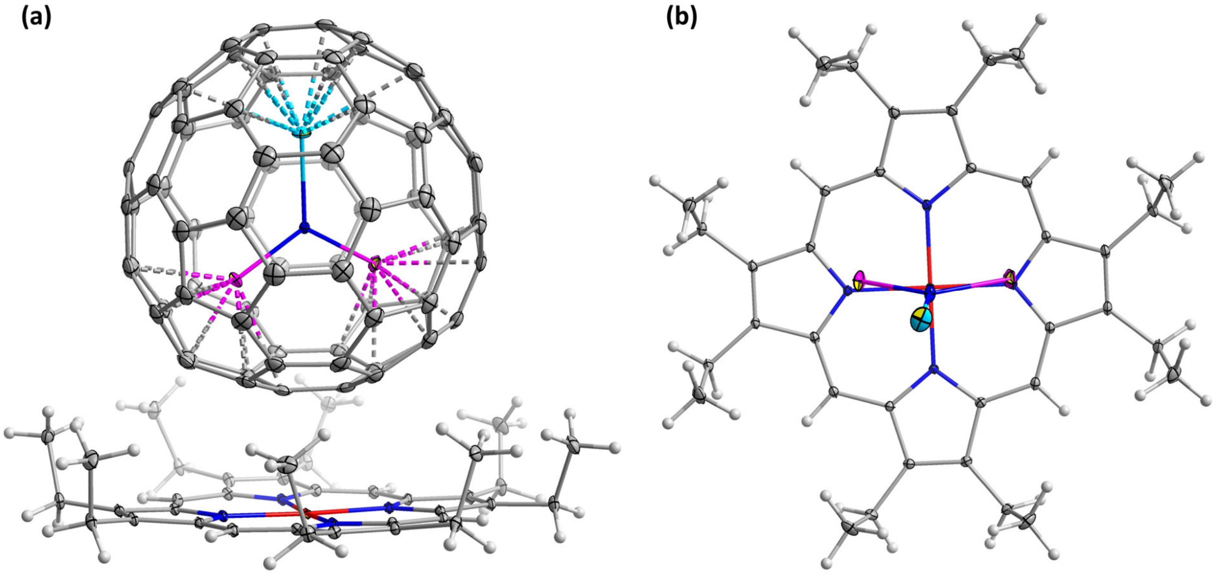

Figure S6. (a) Single-crystal X-ray structures of $NdSc_2N@C_{80}\cdot NiOEP\cdot 2C_6H_6$, the solvent molecules are omitted for clarity. (b) View on the same unit along Nd–N bond; to highlight the relative position of the encapsulated $NdSc_2N$ to the NiOEP, the fullerene cage C_{80} and the solvent molecules are omitted. The displacement parameters are shown at the 30% probability. Color code: grey for carbon, blue for nitrogen, white for hydrogen, red for nickel, pink for scandium, and cyan for neodymium.

DFT calculations of molecular structures

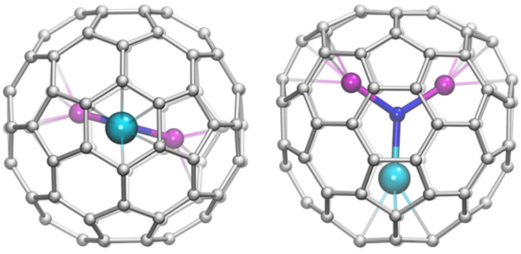

SC-XRD, Nd–N: 2.170 Å

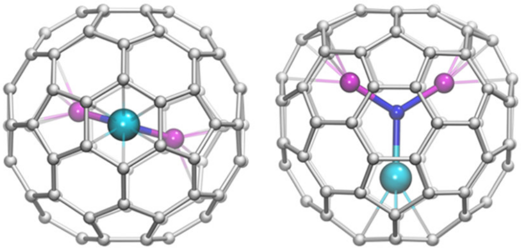

Conf Sc-1, Δ*E* = 0.0 kJ mol^{-1}, Nd–N: 2.224 Å

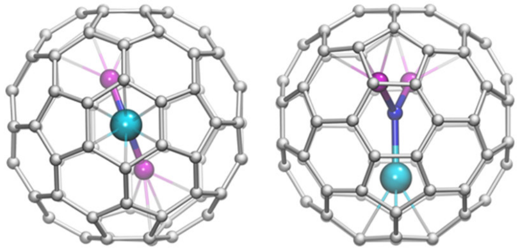

Conf Sc-4, Δ*E* = 2.6 kJ mol^{-1}, Nd–N: 2.222 Å

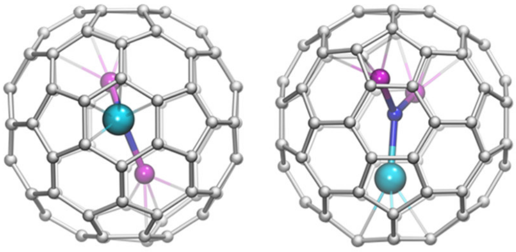

Conf Sc-2, Δ*E* = 2.5 kJ mol^{-1}, Nd–N: 2.225 Å

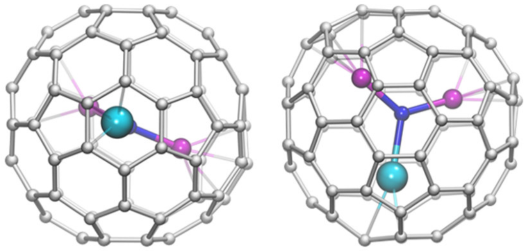

Conf Sc-5, Δ*E* = 3.1 kJ mol^{-1}, Nd–N: 2.227 Å

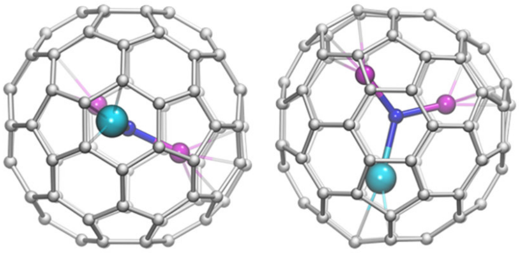

Conf Sc-3, Δ*E* = 2.6 kJ mol^{-1}, Nd–N: 2.221 Å

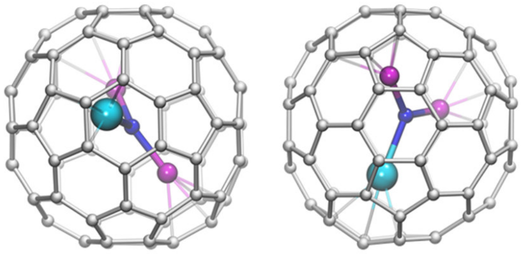

Conf Sc-6, Δ*E* = 9.0 kJ mol^{-1}, Nd–N: 2.232 Å

Figure S7. Six unique conformers of $NdSc_2N@C_{80}$ (conf **Sc-1** – conf **Sc-6**) found in DFT survey of 120 initial configurations, with their relative energies and DFT-optimized Nd–N bond lengths. Each structure is shown in two orientations. For comparison, SC-XRD structure is also shown on top. Orientation of the $NdSc_2N$ cluster in the most stable conf **Sc-1** is very similar to the experimental structure. Color code: Nd – cyan, Sc – magenta, N – blue, C – light gray. Sc–C distances shorter than 2.4 Å for and Nd–C distances shorter than 2.6 Å are shown as bonds.

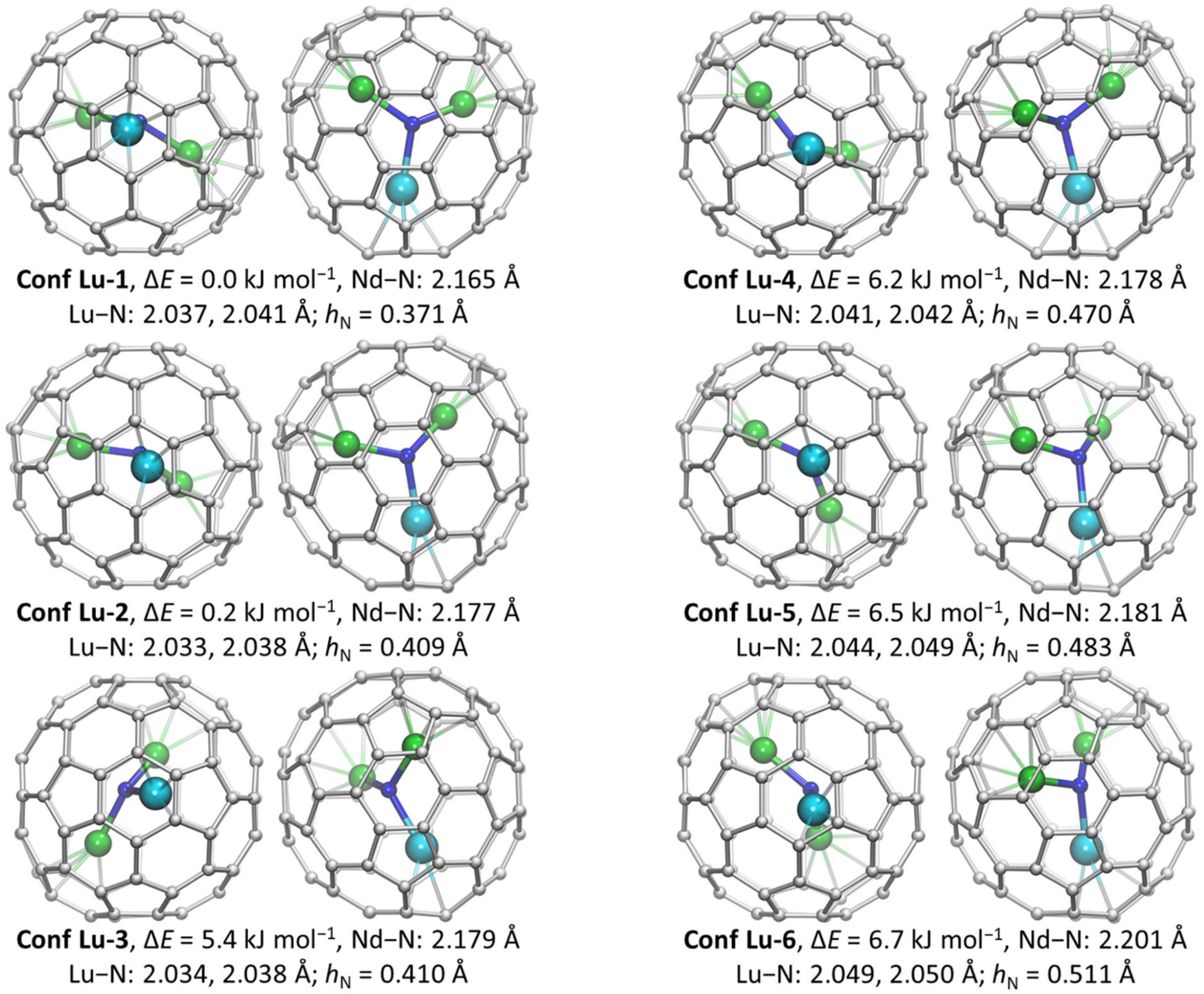

Figure S8. Six lowest-energy conformers of $NdLu_2N@C_{80}$ (conf **Lu-1** – conf **Lu-6**) found in DFT survey with their relative energies and selected structural parameters (h_N is the height of the pyramid formed by elevation of nitrogen above the plane of three metals). Color code: Nd – cyan, Lu – green, N – blue, C – light gray. Lu–C distances shorter than 2.5 Å for and Nd–C distances shorter than 2.6 Å are shown as bonds.

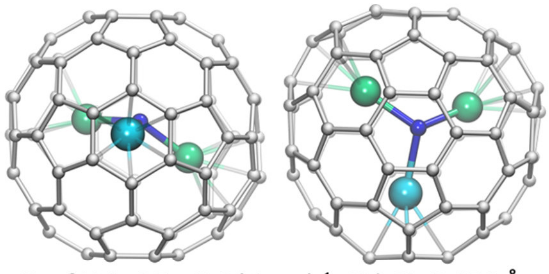

Conf Y-1, ΔE = 0.0 kJ mol^{-1}, Nd–N: 2.170 Å
Y–N: 2.060, 2.062 Å; h_N = 0.523 Å

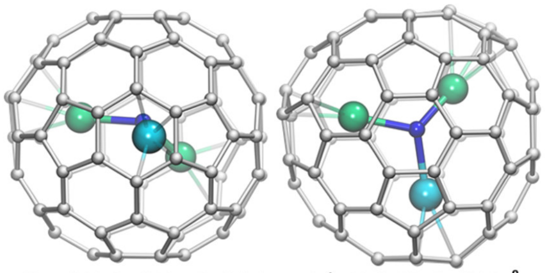

Conf Y-2, ΔE = 3.8 kJ mol^{-1}, Nd–N: 2.174 Å
Y–N: 2.061, 2.066 Å; h_N = 0.538 Å

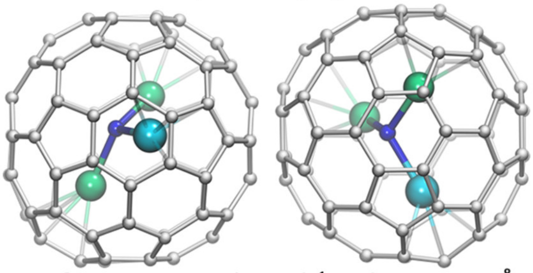

Conf Y-3, ΔE = 6.4 kJ mol^{-1}, Nd–N: 2.176 Å
Y–N: 2.064, 2.068 Å; h_N = 0.552 Å

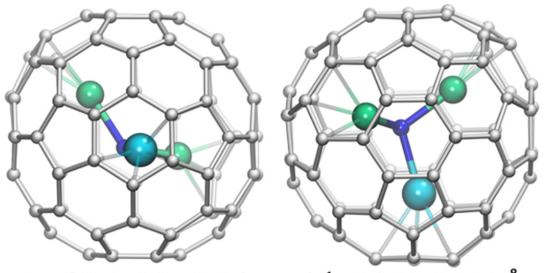

Conf Y-4, ΔE = 5.9 kJ mol^{-1}, Nd–N: 2.188 Å
Y–N: 2.069, 2.072 Å; h_N = 0.604 Å

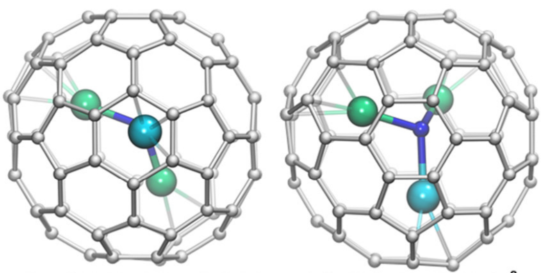

Conf Y-5, ΔE = 6.9 kJ mol^{-1}, Nd–N: 2.183 Å
Y–N: 2.075, 2.077 Å; h_N = 0.624 Å

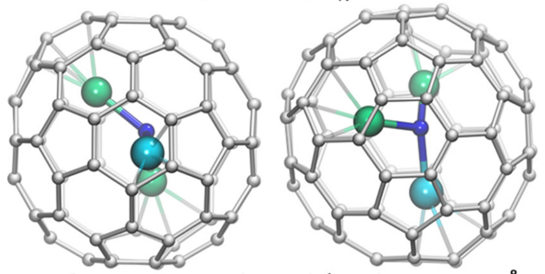

Conf Y-6, ΔE = 6.7 kJ mol^{-1}, Nd–N: 2.178 Å
Y–N: 2.070, 2.072 Å; h_N = 0.580 Å

Figure S9. Six lowest-energy conformers of $NdY_2N@C_{80}$ (**c**onf **Y-1** – conf **Y-6**) found in DFT survey with their relative energies and selected structural parameters (h_N is the height of the pyramid formed by elevation of nitrogen above the plane of three metals). Color code: Nd – cyan, Y – green, N – blue, C – light gray. Y–C distances shorter than 2.5 Å for and Nd–C distances shorter than 2.6 Å are shown as bonds.

Photoluminescence decay

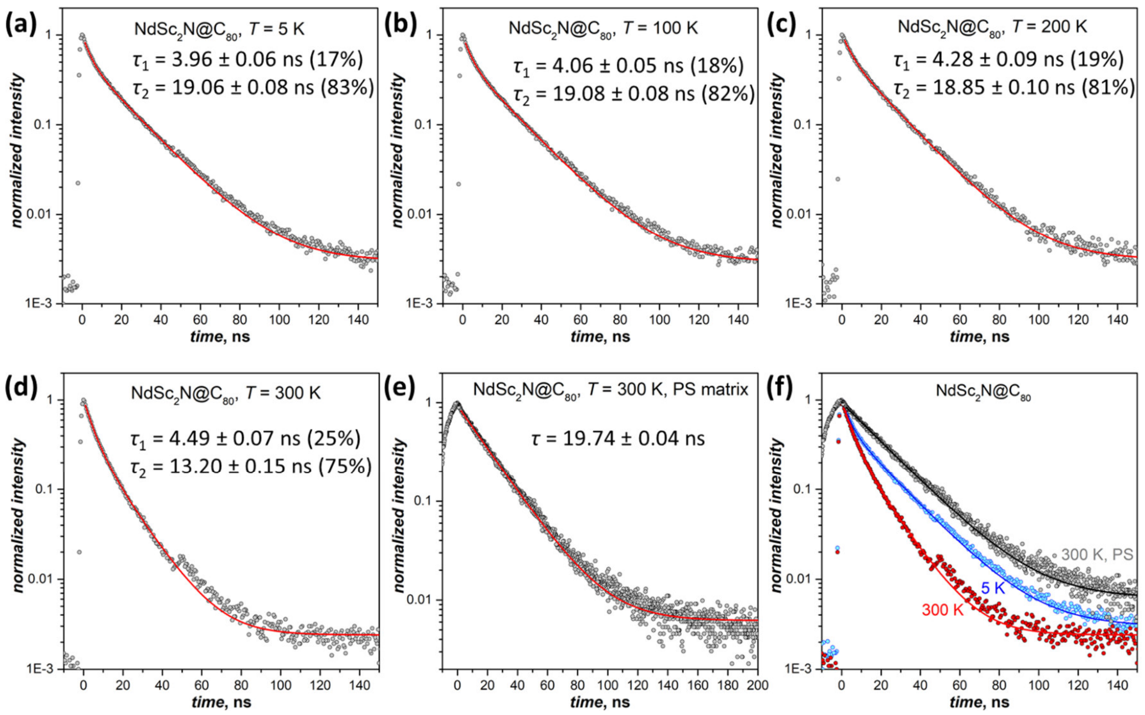

Figure S10. Luminescence decay curves of $NdSc_2N@C_{80}$, laser excitation at 488 nm. (a)-(d) polycrystalline sample, 5–300 K, fit with bi-exponential decay; (e) polystyrene (PS) matrix, 300 K, fit with mono-exponential decay; (f) comparison of polycrystalline (5 K, blue; 300 K, red) and polystyrene (300 K, gray).

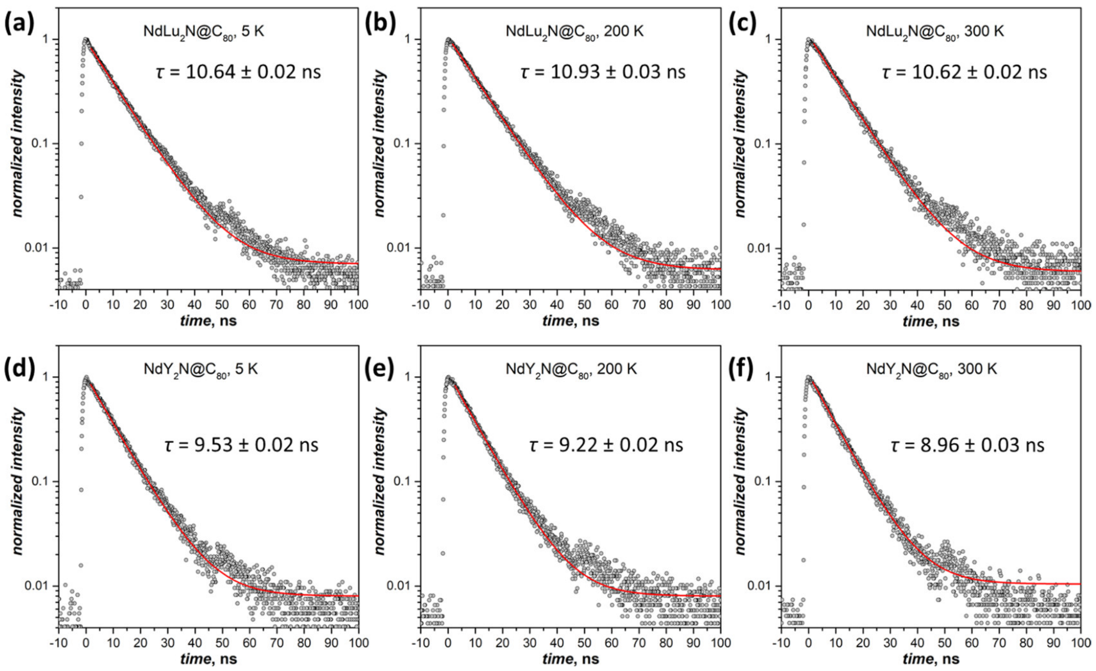

Figure S11. Luminescence decay curves of polycrystalline $NdLu_2N@C_{80}$ (a-c) and $NdY_2N@C_{80}$ (d-e), laser excitation at 488 nm; T = 5 K, 200 K, and 300 K.

Photoluminescence spectra

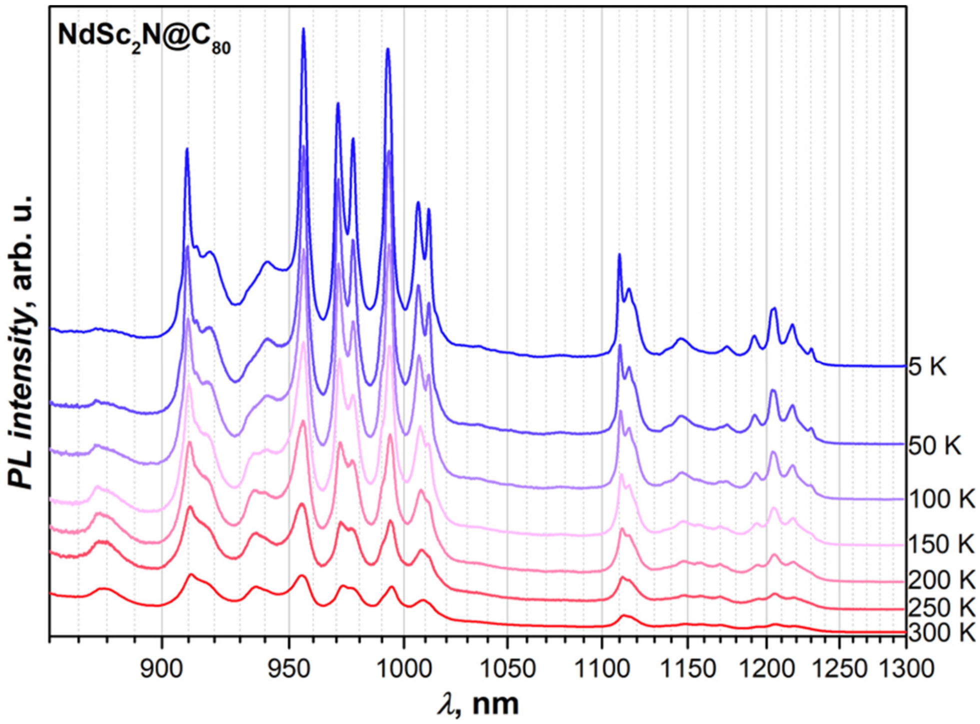

Figure S12. Photoluminescence spectra of polycrystalline $NdSc_2N@C_{80}$ in the range of 860–1300 nm ($^4F_{3/2}\rightarrow{}^4I_{9/2}$ and $^4F_{3/2}\rightarrow{}^4I_{11/2}$ bands) measured at different temperatures, excitation with 488 nm laser line.

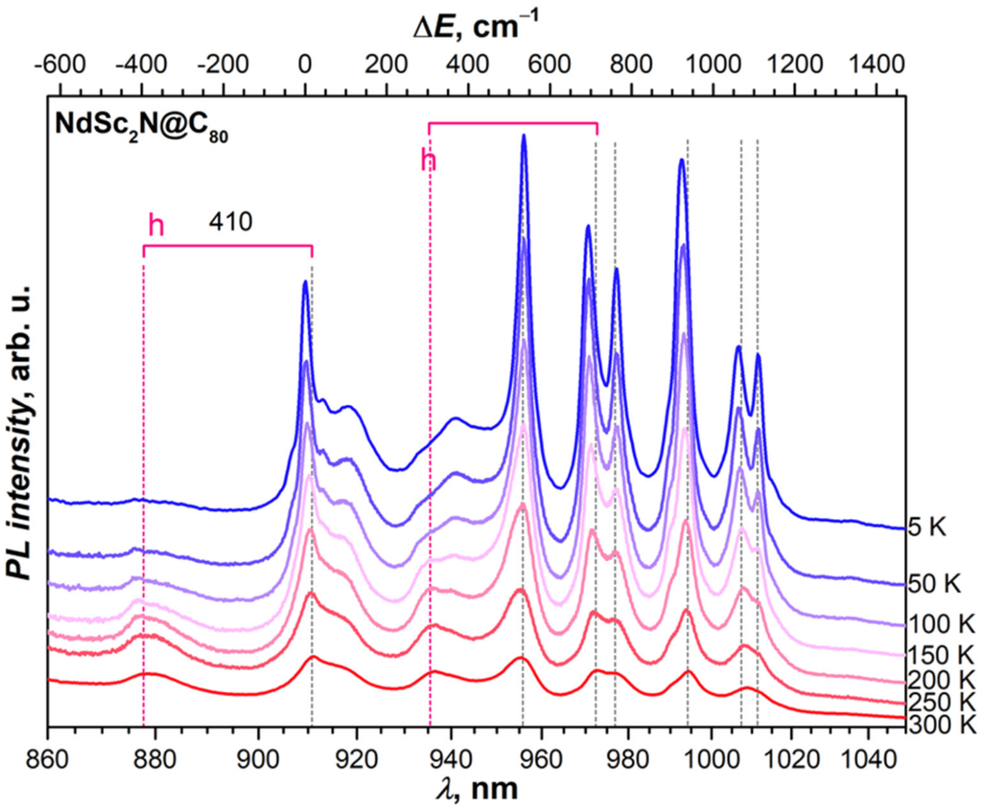

Figure S13. VT-PL spectra of polycrystalline $NdSc_2N@C_{80}$ in the range of the $^4F_{3/2}{\rightarrow}^4I_{9/2}$ band, excitation with 488 nm laser line. The features disappearing upon cooling are assigned to hot bands and marked with **h**, the ligand field splitting in the $^4F_{3/2}$ excited state is estimated as 385 cm^{-1}.

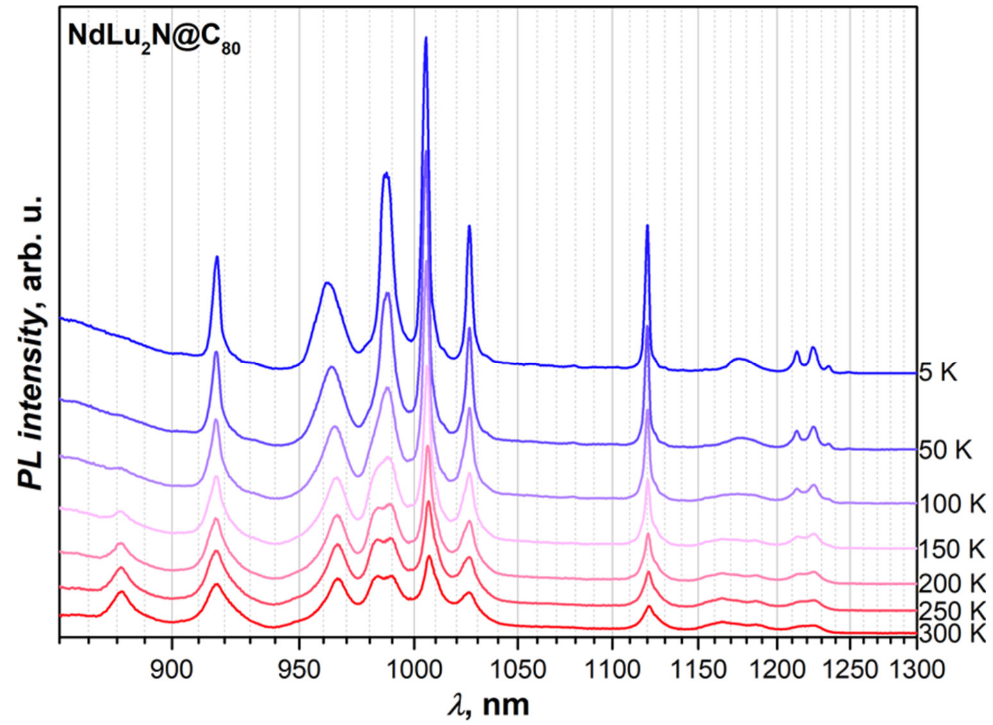

Figure S14. Photoluminescence spectra of polycrystalline $NdLu_2N@C_{80}$ in the range of 860–1300 nm ($^4F_{3/2}{\rightarrow}^4I_{9/2}$ and $^4F_{3/2}{\rightarrow}^4I_{11/2}$ bands) measured at different temperatures, excitation with 488 nm laser line.

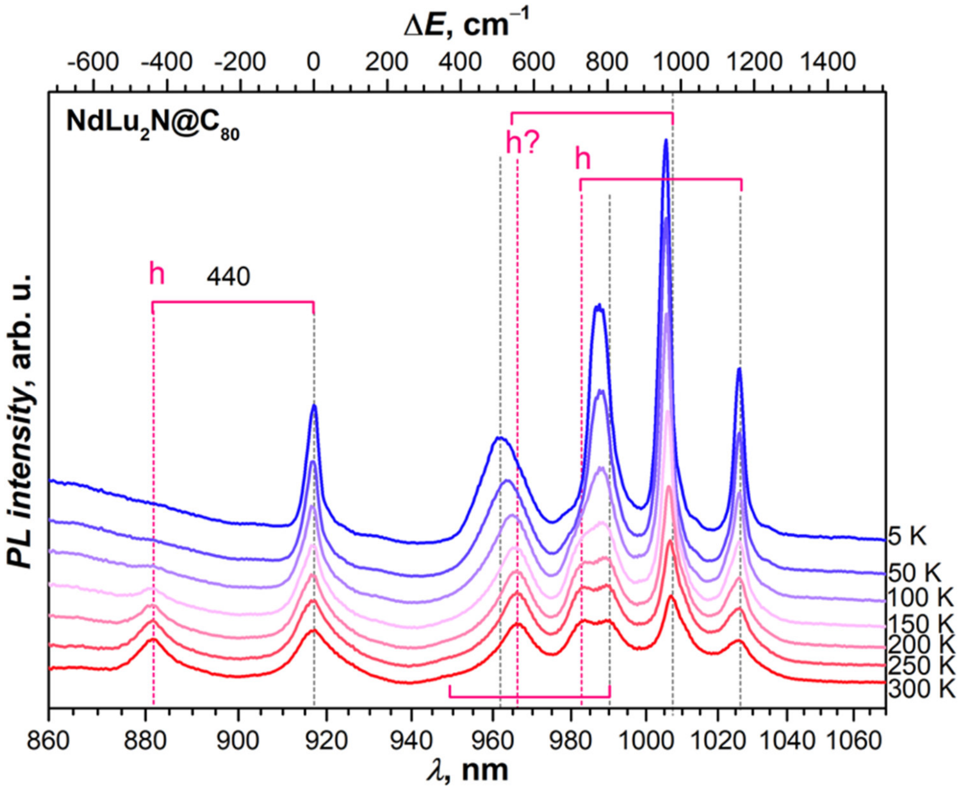

Figure S15. VT-PL spectra of polycrystalline $NdLu_2N@C_{80}$ in the range of the $^4F_{3/2}\rightarrow{}^4I_{9/2}$ band, excitation with 488 nm laser line. The features disappearing upon cooling are assigned to hot bands and marked with **h**, the ligand field splitting in the $^4F_{3/2}$ excited state is estimated as 440 cm^{-1}. Assignment of the peak at 960-965 nm is rather ambiguous. The temperature shift of this peak is considerably stronger than for other peaks, and we suggest that the two features occur at close energies: the hot band visible at room temperature, and the broadened KD transition, which becomes distinguishable at lower temperature, when intensity of the hot band gradually decreases. The apparent shift during cooling is the result of the hot band disappearance.

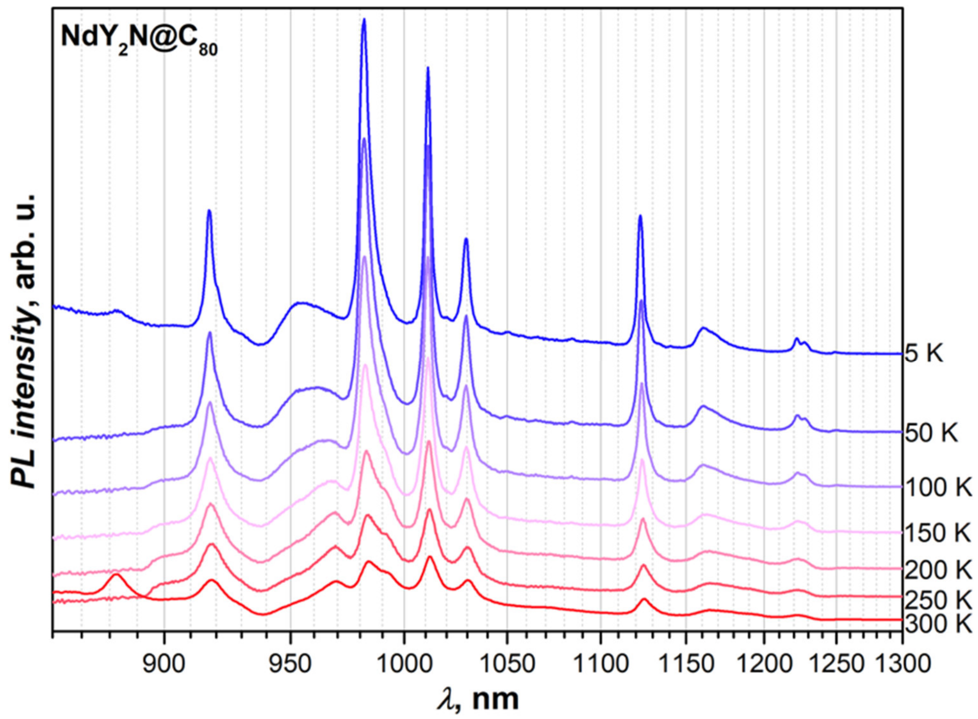

Figure S16. Photoluminescence spectra of polycrystalline $NdY_2N@C_{80}$ in the range of 860–1300 nm ($^4F_{3/2}\rightarrow{}^4I_{9/2}$ and $^4F_{3/2}\rightarrow{}^4I_{11/2}$ bands) measured at different temperatures, excitation with 488 nm laser line. In the spectra measured between 50 K and 250 K, the bandpass filter with a threshold of 890 nm cuts the short-wavelengths part, thus the hot band at 880 nm is not seen.

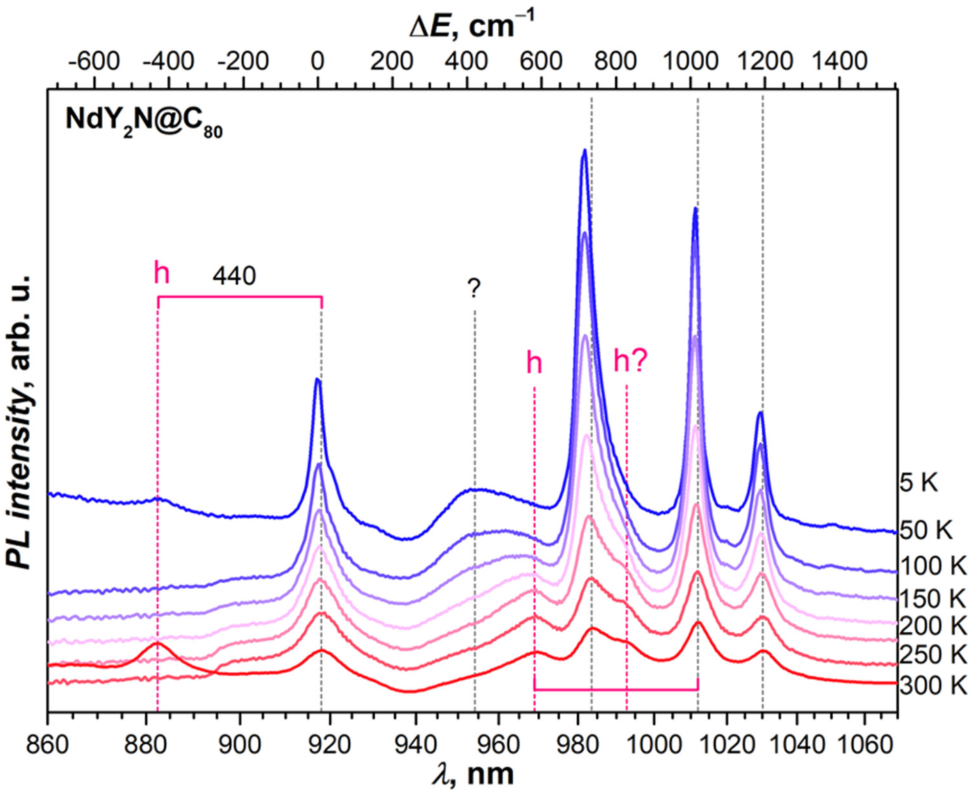

Figure S17. VT-PL spectra of polycrystalline $NdY_2N@C_{80}$ in the range of the $^4F_{3/2}\rightarrow{}^4I_{9/2}$ band, excitation with 488 nm laser line. The features disappearing upon cooling are assigned to hot bands and marked with **h**, the ligand field splitting in the $^4F_{3/2}$ excited state is estimated as 440 cm^{-1}. In the spectra measured between 50 K and 250 K, the bandpass filter with a threshold of 890 nm cuts the short-wavelengths part, thus the hot band at 880 nm is not seen. Assignment of the broad asymmetric peak at 955 nm ($\Delta E \approx 400$ cm^{-1}), which becomes visible only at low temperature, is not very clear. We tentatively assign it to the transition ending at KD2 of the ground state multiplet, but it remains ambiguous because of its very large width different from features of all other pure transitions of this type. Another feature with questionable assignment is at 992 nm (labelled “h?”). Its temperature evolution corresponds to that of hot bands, but the energy does not match.

Table S2. LF splitting in ^4I_J and $^4F_{3/2}$ multiplets of $NdSc_2N@C_{80}$, $NdLu_2N@C_{80}$, and $NdY_2N@C_{80}$, determined from low-temperature PL measurements.

multiplet	KD	free ion	$NdSc_2N@C_{80}$ *E*	ΔE	$NdLu_2N@C_{80}$ *E*	ΔE		$NdY_2N@C_{80}$ *E*	ΔE	
$^4I_{9/2}$	KD1	0	0	0	0	0		0	0	
	KD2			536		510	v br		420	v br
	KD3			693/763		775			711	
	KD4			923		958			1005	
	KD5			1061/1110	1161	1161		1188	1188	
$^4I_{11/2}$	KD1	1897	1987	0	1976	0		1995	0	
	KD2			492		430	v br		295	v br
	KD3			618		687			725	
	KD4			701/712		761			760	
	KD5			793		832			815	sh, vw
	KD6		2869	880	2899	923	vw	2895	900	vw
$^4I_{13/2}$	KD1	3907	3890	0						
	KD2			535						
	KD3			632	4318					
	KD4			682	4435			4299		
	KD5			788	4572			4420		
	KD6			810	4659			4607		
	KD7		4781	890	4695			4668		
$^4F_{3/2}$	KD1	11699	10996	0	10906	0		10901	0	
	KD2		11406	410	11346	440		11341	440	

The free-ion energies of Nd^{3+} multiplets are from Ref. [14]

For the $^4I_{13/2}$ multiplet in $NdLu_2N@C_{80}$ and $NdY_2N@C_{80}$, we cannot identify KD1 peaks and thus ΔE values are not available, and the table lists absolute values of observed features. Their assignment to particular KDs is ambiguous.

Additional designations: "v br" – very broad, "vw" – very weak, "sh" – shoulder

Ab initio calculations of ligand-field splitting, pseudospin g-tensors, and state compositions

Table S3a. Ligand-field splitting of Nd^{3+}-$^4I_{9/2}$ multiplet in $NdSc_2N@C_{80}$ (**Conf Sc-1**) computed at the CASSCF/RASSI level.

KD	exp. *E* cm^{-1}	calc. *E* cm^{-1}	% composition in |*J*, *m$_J$*⟩ basis (*J* = 9/2)	g_x	g_y	g_z	ϕ°
1	0	0	95|±9/2⟩	0.1383	0.2390	6.1923	0
2	536	80	79|±7/2⟩ + 19|±5/2⟩	0.4280	0.7394	4.5918	21
3	693/763	435	82|±1/2⟩ + 12|±3/2⟩	2.3892	4.0148	0.9780	10
4	923	594	45|±3/2⟩ + 37|±5/2⟩ + 11|±7/2⟩	3.6651	2.8343	0.0690	13
5	1061/1110	693	42|±5/2⟩ + 40|±3/2⟩ + 9|±1/2⟩ + 8|±7/2⟩	3.7346	2.4854	1.0045	4

total contribution of B_q^2, B_q^4, and B_q^6 terms to LF splitting: 94.2%; angle between g_z axis of KD1 and Nd–N bond is 7.6°

Table S3b. Ligand-field splitting of Nd^{3+}-$^4I_{9/2}$ multiplet in $NdSc_2N@C_{80}$ (**Conf Sc-2**) computed at the CASSCF/RASSI level.

KD	exp. *E* cm^{-1}	calc. *E* cm^{-1}	% composition in |*J*, *m$_J$*⟩ basis (*J* = 9/2)	g_x	g_y	g_z	ϕ°
1	0	0	89|±9/2⟩ + 6|±3/2⟩	0.1089	0.1440	5.8548	0
2	536	245	46|±5/2⟩ + 37|±7/2⟩ + 8|±3/2⟩ + 5|±9/2⟩	0.4295	0.6190	5.2451	42
3	693/763	506	42|±3/2⟩ + 25|±1/2⟩ + 18|±5/2⟩ + 11|±7/2⟩	0.5248	1.4336	4.7754	65
4	923	687	37|±7/2⟩ + 28|±3/2⟩ + 20|±5/2⟩ + 15|±1/2⟩	0.2309	1.9113	4.3511	55
5	1061/1110	795	55|±1/2⟩ + 17|±3/2⟩ + 14|±7/2⟩ + 13|±5/2⟩	3.6984	2.9214	0.7692	4

total contribution of B_q^2, B_q^4, and B_q^6 terms to LF splitting: 94.7%; angle between g_z axis of KD1 and Nd–N bond is 12.8°

Table S3c. Ligand-field splitting of Nd^{3+}-$^4I_{9/2}$ multiplet in $NdSc_2N@C_{80}$ (**Conf Sc-3**) computed at the CASSCF/RASSI level.

KD	exp. *E* cm^{-1}	calc. *E* cm^{-1}	% composition in |*J*, *m$_J$*⟩ basis (*J* = 9/2)	g_x	g_y	g_z	ϕ°
1	0	0	85|±9/2⟩ + 7|±5/2⟩ + 6|±3/2⟩	0.0371	0.0445	5.6890	0
2	536	467	48|±5/2⟩ + 25|±7/2⟩ + 19|±3/2⟩ + 8|±1/2⟩	0.5312	1.3555	3.1828	21
3	693/763	648	31|±3/2⟩ + 29|±5/2⟩ + 19|±7/2⟩ + 14|±9/2⟩	0.0369	1.0730	3.6643	25
4	923	786	48|±7/2⟩ + 30|±3/2⟩ + 14|±1/2⟩ + 8|±5/2⟩	2.0524	0.3009	3.7618	55
5	1061/1110	968	70|±1/2⟩ + 14|±3/2⟩ + 8|±5/2⟩ + 8|±7/2⟩	4.3734	2.4200	0.4013	7

total contribution of B_q^2, B_q^4, and B_q^6 terms to LF splitting: 95.7%; angle between g_z axis of KD1 and Nd–N bond is 5.1°

Table S3d. Ligand-field splitting of Nd^{3+}-$^{4}I_{9/2}$ multiplet in $NdSc_2N@C_{80}$ (**conf Sc-4**) computed at the CASSCF/RASSI level.

KD	exp. *E* cm^{-1}	calc. *E* cm^{-1}	% composition in |*J*, *m_J*⟩ basis (*J* = 9/2)	g_x	g_y	g_z	ϕ°
1	0	0	95|±9/2⟩	0.0689	0.1472	6.1974	0
2	536	80	89|±7/2⟩ + 10|±5/2⟩	0.5051	0.6936	4.5465	15
3	693/763	421	80|±1/2⟩ + 13|±3/2⟩	4.9986	1.4867	0.7820	6
4	923	601	43|±3/2⟩ + 40|±5/2⟩ + 13|±1/2⟩	2.2240	4.3204	0.1389	10
5	1061/1110	690	46|±5/2⟩ + 42|±3/2⟩	3.4567	2.9098	1.0030	3

total contribution of B_q^2, B_q^4, and B_q^6 terms to LF splitting: 95.0%; angle between g_z axis of KD1 and Nd–N bond is 5.4°

Table S3e. Ligand-field splitting of Nd^{3+}-$^{4}I_{9/2}$ multiplet in $NdSc_2N@C_{80}$ (**conf Sc-5**) computed at the CASSCF/RASSI level.

KD	exp. *E* cm^{-1}	calc. *E* cm^{-1}	% composition in |*J*, *m_J*⟩ basis (*J* = 9/2)	g_x	g_y	g_z	ϕ°
1	0	0	85|±9/2⟩ + 9|±5/2⟩ + 4|±3/2⟩	0.0598	0.0680	5.7433	0
2	536	407	39|±5/2⟩ + 33|±7/2⟩ + 22|±3/2⟩ + 4|±1/2⟩	0.3974	1.0496	4.4761	48
3	693/763	605	37|±5/2⟩ + 32|±3/2⟩ + 12|±9/2⟩ + 11|±1/2⟩	0.0528	0.5621	4.3728	64
4	923	740	50|±7/2⟩ + 25|±3/2⟩ + 17|±1/2⟩ + 7|±5/2⟩	3.6471	0.5442	2.6493	45
5	1061/1110	907	65|±1/2⟩ + 18|±3/2⟩ + 9|±5/2⟩ + 8|±7/2⟩	4.4537	2.3083	0.3923	5

total contribution of B_q^2, B_q^4, and B_q^6 terms to LF splitting: 95.9%; angle between g_z axis of KD1 and Nd–N bond is 9.0°

Table S3f. Ligand-field splitting of Nd^{3+}-$^{4}I_{9/2}$ multiplet in $NdSc_2N@C_{80}$ (**conf Sc-6**) computed at the CASSCF/RASSI level.

KD	exp. *E* cm^{-1}	calc. *E* cm^{-1}	% composition in |*J*, *m_J*⟩ basis (*J* = 9/2)	g_x	g_y	g_z	ϕ°
1	0	0	84|±9/2⟩ + 10|±3/2⟩ + 4|±5/2⟩	0.0284	0.0322	5.6347	0
2	536	471	57|±5/2⟩ + 21|±7/2⟩ + 13|±3/2⟩ + 6|±3/2⟩	0.8164	1.2241	3.5071	38
3	693/763	653	44|±3/2⟩ + 25|±7/2⟩ + 14|±5/2⟩ + 11|±9/2⟩	0.9084	1.1718	3.3145	29
4	923	868	42|±7/2⟩ + 26|±3/2⟩ + 17|±5/2⟩ + 14|±1/2⟩	0.6707	1.9142	3.4280	40
5	1061/1110	1018	66|±1/2⟩ + 15|±3/2⟩ + 10|±7/2⟩ + 8|±5/2⟩	3.9823	2.6212	0.5579	13

total contribution of B_q^2, B_q^4, and B_q^6 terms to LF splitting: 94.9%; angle between g_z axis of KD1 and Nd–N bond is 0.6°

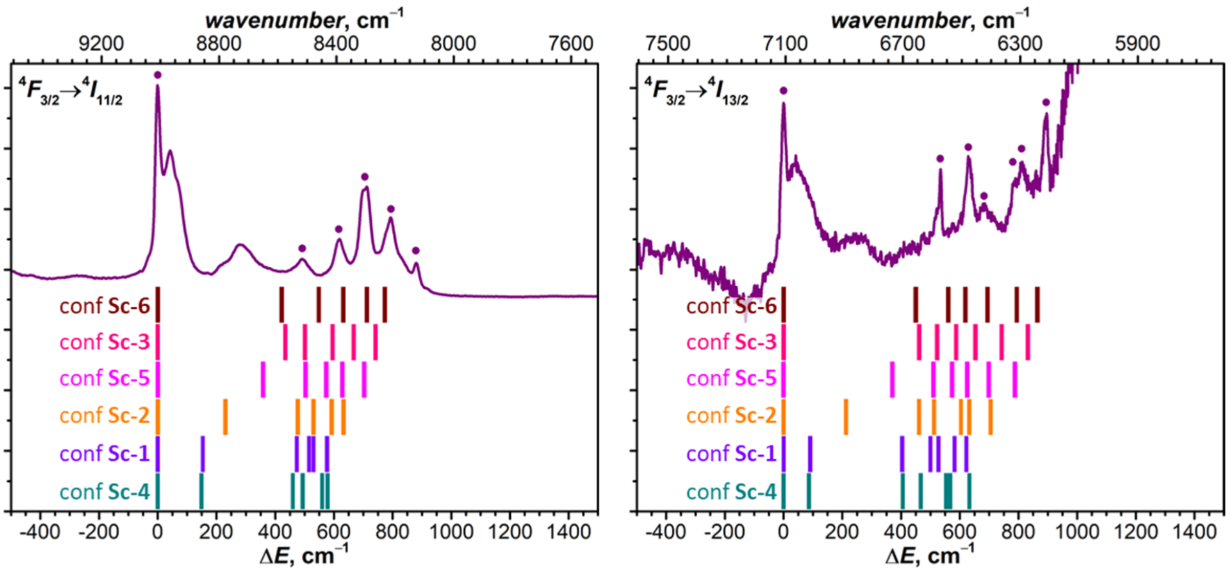

Figure S18. The fine structure of $^4F_{3/2}\rightarrow{}^4I_{11/2}$ (left) and $^4F_{3/2}\rightarrow{}^4I_{13/2}$ (right) photoluminescence bands of $NdSc_2N@C_{80}$ measured at 5 K compared to LF splitting of $^4I_{11/2}$ and $^4I_{13/2}$ multiplets in six conformers of $NdSc_2N@C_{80}$ from CASSCF calculations (see Fig. S7 for molecular structures and Tables S3a-S3f for energies of LF states). While conformers **Sc-3** and **Sc-6** give reasonable agreement to the experimental spectra of LF states (except for some systematic underestimation of the splitting by ca 10%), the lowest-energy conformer **Sc-1** is far off. Surprisingly, it appears that only the conformers, in which Nd is coordinated close to one C atom in η^1-η^3 manner, contribute to the fine structure of PL spectra. At the same time, the broader features at lower energies can be caused by other conformers.

Table S4. Experimental (from PL measurements) and calculated (CASSCF) LF splitting in ^4I_J and $^4F_{3/2}$ multiplets of $NdSc_2N@C_{80}$.

multiplet	KD	free ion	exp. PL (5 K) *E*	ΔE	Conf 3 *E*	ΔE	Conf 5 *E*	ΔE	Conf 6 *E*	ΔE
$^4I_{9/2}$	KD1	0	0	0	0	0	0	0	0	0
	KD2			536		467		407		471
	KD3			693/763		648		605		653
	KD4			923		786		740		868
	KD5			1061/1110		968		907		1018
$^4I_{11/2}$	KD1	1897	1987	0	1845	0	1826	0	1854	0
	KD2			492		434		359		422
	KD3			618		501		503		549
	KD4			701/712		595		574		632
	KD5			793		667		629		712
	KD6			880		741		703		773
$^4I_{13/2}$	KD1	3907	3890	0	3828	0	3813	0	3837	0
	KD2			535		461		370		450
	KD3			632		523		510		561
	KD4			682		587		574		619
	KD5			788		653		626		695
	KD6			810		742		699		794
	KD7			890		832		788		865
$^4I_{15/2}$	KD1	5989			5984	0	5982	0	5982	0
	KD2					545		494		566
	KD3					662		578		692
	KD4					779		723		789
	KD5					805		762		864
	KD6					949		910		1025
	KD7					1053		992		1129
	KD8					1200		1141		1252
$^4F_{3/2}$	KD1	11699	10996	0	15879	0	15841	0	15887	0
	KD2			410		401		393		421

Free-ion energies of Nd^{3+} multiplets are from Ref. [14]

$NdSc_2N@C_{80}$, conformer Sc-3: CASSCF versus point charge model

Table S5a. Ligand-field splitting and pseudospin g-tensors of Kramers doublets of Nd^{3+}-$^4I_{9/2}$ multiplet in $NdSc_2N@C_{80}$ (conf **Sc-3**) computed with point charge model (PCM)*

KD	*E*, cm^{-1} CASSCF	*E* ^{a}cm^{-1} PCM	% composition in |9/2, m_J⟩ basis	g_x	g_y	g_z
1	0	0	99.9|±9/2⟩	0.0000	0.0000	6.5437
2	467	466	99.6|±7/2⟩	0.0013	0.0014	5.0823
3	648	643	99.0|±5/2⟩	0.0937	0.1002	3.6158
4	786	770	95.0|±3/2⟩ + 4.4|±1/2⟩	1.4408	1.4927	2.0604
5	968	867	94.8|±1/2⟩ + 4.5|±3/2⟩	5.0352	2.0948	0.6166

*PCM used LoProp charges from CASSCF calculations scaled by a factor of 1.08 to reproduce the $\Delta_{1,2}$ value

Table S5b. Ligand-field splitting and pseudospin g-tensors of Kramers doublets of Nd^{3+}-$^4I_{9/2}$ multiplet computed at the CASSCF/RASSI level for Nd^{3+} ion surrounded by point charges in positions of all other atoms in $NdSc_2N@C_{80}$ (conf **Sc-3**), the charges are the same as used for calculations in Table S5a.

KD	*E* cm^{-1} CASSCF	*E* cm^{-1} CASSCF (Nd)+PCM	% composition in |9/2, m_J⟩ basis	g_x	g_y	g_z
1	0	0	99.9|±9/2⟩	0.0001	0.0001	6.3448
2	467	466	99.3|±7/2⟩	0.0030	0.0031	4.9384
3	648	626	98.2|±5/2⟩	0.2012	0.2244	3.4771
4	786	724	91.3|±3/2⟩+ 7.7|±1/2⟩	1.7958	1.8647	2.0487
5	968	806	91.1|±1/2⟩ + 7.9|±3/2⟩	5.4664	1.5738	0.4829

Table S5c. Ligand-field splitting and pseudospin g-tensors of Kramers doublets of Nd^{3+}-$^4I_{9/2}$ multiplet computed at the CASSCF/RASSI level for $(NdSc_2N)^{6+}$ cluster surrounded by point charges at positions of carbon atoms in $NdSc_2N@C_{80}$ (conf **Sc-3**).

KD	*E* cm^{-1} CASSCF	*E* cm^{-1} CASSCF ($NdSc_2N^{6+}$)+PCM	% composition in |9/2, m_J⟩ basis	g_x	g_y	g_z
1	0	0	100|±9/2⟩	0.0004	0.0004	6.3330
2	467	417	62|±7/2⟩ + 25|±3/2⟩ + 11|±5/2⟩	0.6263	1.9495	3.9303
3	648	430	81|±5/2⟩ + 8|±7/2⟩ + 5|±1/2⟩ + 5|±3/2⟩	1.0218	1.3629	3.1031
4	786	505	64|±3/2⟩ + 29|±7/2⟩ + 6|±1/2⟩	1.2357	2.0019	2.8027
5	968	580	87|±1/2⟩ + 7|±3/2⟩ + 6|±5/2⟩	5.3387	1.8260	0.6077

Table S5d. Ligand-field splitting and pseudospin g-tensors of Kramers doublets of Nd^{3+}-$^4I_{9/2}$ multiplet in $NdSc_2N@C_{80}$ (conf **Sc-1**) computed with point charge model (PCM) and LoProp charges scaled by 1.08.

KD	*E*, cm^{-1} CASSCF	*E* ^{a}cm^{-1} PCM	% composition in |9/2, m_J⟩ basis	g_x	g_y	g_z
1	0	0	100|±9/2⟩	0.0002	0.0002	6.5446
2	80	454	99.8|±7/2⟩	0.0142	0.0149	5.0859
3	435	630	99.5|±5/2⟩	0.0655	0.0937	3.6244
4	594	757	97.1|±3/2⟩ + 2.6|±1/2⟩	1.1010	1.1942	2.1074
5	693	847	96.9|±1/2⟩ + 2.7|±3/2⟩	4.7423	2.4437	0.6607

CASSCF calculations of $Nd(C_5Me_5)_3$ and $Nd(C_5Me_4H)_3$

Table S6a. Ligand-field splitting and pseudospin g-tensors of Kramers doublets of Nd^{3+}-$^4I_{9/2}$ multiplet computed at the CASSCF/RASSI level for $Nd(C_5Me_5)_3$.

KD	*E* exp PL	*E* CASSCF	% composition in |9/2, m_J⟩ basis	g_x	g_y	g_z
1	0	0	76.4|±5/2⟩ + 23.6|±7/2⟩	2.1691	2.1330	1.4712
2	36	29	90.2|±3/2⟩ + 9.8|±9/2⟩	0.0134	0.0241	1.0526
3	202	157	100.0|±1/2⟩	3.6536	3.6421	0.5476
4	699	441	90.2|±9/2⟩ + 9.8|±3/2⟩	0.0002	0.0009	5.5844
5	861	531	76.4|±7/2⟩ + 23.6|±5/2⟩	2.3336	2.3358	3.1396

Experimental data are determined by luminescence at room temperature in Ref [15]

Table S6b. Ligand-field splitting and pseudospin g-tensors of Kramers doublets of Nd^{3+}-$^4I_{9/2}$ multiplet computed at the CASSCF/RASSI level for $Nd(C_5Me_4H)_3$.

KD	E exp abs	*E* CASSCF	% composition in |9/2, m_J⟩ basis	g_x	g_y	g_z
1	0	0	74.1|±5/2⟩ + 25.9|±7/2⟩	2.2126	2.1912	1.2232
2	70	40	89.1|±3/2⟩ + 10.9|±9/2⟩	0.0085	0.0140	0.8978
3	265	181	100.0|±1/2⟩	3.6384	3.6313	0.5129
4	800	517	89.2|±9/2⟩ + 10.8|±3/2⟩	0.0002	0.0042	5.4672
5	1000	629	74.1|±7/2⟩ + 25.9|±5/2⟩	2.4205	2.4282	2.8993

Experimental data are determined by various optical spectroscopic techniques in Ref [16]

Our *ab initio* calculations for $Nd(\eta^5\text{-}C_5Me_5)_3$ and $Nd(\eta^5\text{-}C_5Me_4H)_3$ gave the Δ_{LF} values of 532 cm^{-1} and 629 cm^{-1}, respectively (see Table S6), meaning that either CASSCF/RASSI underestimates the LF splitting by as much as 60%, or some of the experimental lines may require re-assignment.

CASSCF calculations for $NdLu_2N@C_{80}$ and $NdY_2N@C_{80}$

Based on the results of CASSCF calculations for conformers of $NdSc_2N@C_{80}$, we chosen two representative conformers of $NdM_2N@C_{80}$ (M = Lu, Y) for *ab initio* calculations, **Lu(Y)-1** and **Lu(Y)-3**. These conformers are different in the way how Nd is coordinated to the fullerene: in **Lu(Y)-1**, coordination is to hexagon nearly of η^6 type, while **Lu(Y)-3** features less central position of Nd above pentagon with η^3 hapticity (Fig. S19). The difference of LF splitting between these conformers appeared to be less pronounced than in $NdSc_2N@C_{80}$ conformers, presumably because even in the case of η^6 coordination, Nd–N axis and g_z axis of KD1 are in facto oriented not to the center of the hexagon but closer to one of carbon atoms.

In CASSCF calculations of $NdLu_2N@C_{80}$, the use of full-electron basis for Lu appeared problematic because of the poor convergence. We therefore performed calculations with DFT-optimized structures of $NdLu_2N@C_{80}$ and using Sc or Y instead of Lu in CASSCF calculations. For consistency, analogous calculations were also performed for $NdY_2N@C_{80}$. Comparison of the results shows that the replacement of Lu with Sc or Y has little effect on the wavefunction composition and only slightly affects pseudospin g-tensors, but has more pronounced influence on the LF splitting. For instance, for conformers **Lu-3** and **Y-3**, the energy of KD2 appeared not sensitive to the use of Sc or Y, while the energies of higher KDs tend to be higher for Y than for Sc by 20–30 cm^{-1}. Considering that ionic radius of Lu is between that of Sc and Y, we used mean values when plotting calculated energies for $NdLu_2N@C_{80}$ in Fig. 3.

It is remarkable that in agreement with experimental spectra, the energy of KD2 in $NdY_2N@C_{80}$ in conformer **Y-3** is ca 50 cm^{-1} smaller than in the analogous conformer $NdLu_2N@C_{80}$, while other KDs have similar energies. Calculated results for conformer **Lu(Y)-1** give higher energy of KD2 for Y than for Lu. We therefore preferred to use conformer **Lu(Y)-3** for comparison with experimental data in Fig. 3, as this conformer displays closer resemblance to experimental spectra.

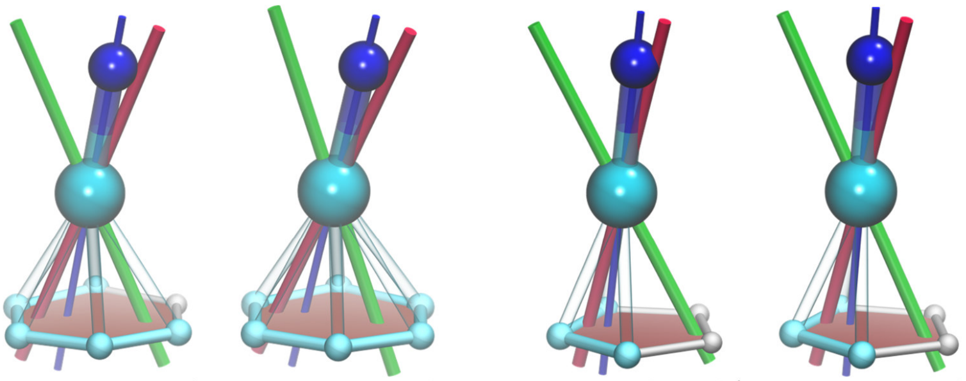

Figure S19. Coordination of Nd atom to the closest carbon atoms in two representative conformers of $NdLu_2N@C_{80}$ and $NdLu_2N@C_{80}$, their geometrical Nd–N axes (thin blue lines) and g_z axes of the two lowest-energy Kramers doublets from CASSCF calculations (KD1 – red lines, KD2 – green lines). Color code: Nd – cyan, N – blue, C – light cyan ($d_{Nd–C} \leq 2.6$ Å) or light gray ($d_{Nd–C} > 2.6$ Å). Nd–C distances shorter than 2.6 Å are shown as bonds. Also listed for each conformer are its relative energy from DFT calculations and $\Delta_{1,2}$ and Δ_{LF} splitting from CASSCF calculations.

Table S7a. Ligand-field splitting of Nd^{3+}-$^4I_{9/2}$ multiplet in $NdLu_2N@C_{80}$ (conf **Lu-1,** Sc instead of Lu) computed at the CASSCF/RASSI level.

KD	exp. *E* cm^{-1}	calc. *E* cm^{-1}	% composition in $|J, m_J\rangle$ basis (J = 9/2)	g_x	g_y	g_z	ϕ°
1	0	0	$86|\pm9/2\rangle + 11|\pm5/2\rangle$	0.0934	0.1048	5.8192	0
2	510	351	$41|\pm7/2\rangle + 29|\pm5/2\rangle + 23|\pm3/2\rangle$	0.4743	0.7057	5.3798	
3	775	589	$36|\pm3/2\rangle + 35|\pm5/2\rangle + 20|\pm1/2\rangle + 6|\pm9/2\rangle$	0.7516	1.7961	4.2842	
4	958	779	$41|\pm7/2\rangle + 28|\pm1/2\rangle + 15|\pm5/2\rangle + 13|\pm3/2\rangle$	3.4005	2.9636	0.0423	
5	1161	909	$48|\pm1/2\rangle + 27|\pm3/2\rangle + 15|\pm7/2\rangle + 10|\pm5/2\rangle$	0.6876	2.3101	4.2050	

total contribution of B_q^2, B_q^4, and B_q^6 terms to LF splitting: 94.8 %; angle between g_z axis of KD1 and Nd–N bond is 11.6°

Table S7b. Ligand-field splitting of Nd^{3+}-$^4I_{9/2}$ multiplet in $NdLu_2N@C_{80}$ (conf **Lu-1,** Y instead of Lu) computed at the CASSCF/RASSI level.

KD	exp. *E* cm^{-1}	calc. *E* cm^{-1}	% composition in $|J, m_J\rangle$ basis (J = 9/2)	g_x	g_y	g_z	ϕ°
1	0	0	$86|\pm9/2\rangle + 11|\pm5/2\rangle$	0.0894	0.1021	5.8208	0
2	510	339	$42|\pm7/2\rangle + 29|\pm5/2\rangle + 21|\pm3/2\rangle + 7|\pm9/2\rangle$	0.4374	0.6147	5.4561	
3	775	597	$37|\pm3/2\rangle + 33|\pm5/2\rangle + 23|\pm1/2\rangle + 5|\pm9/2\rangle$	0.7717	2.0060	4.2083	
4	958	800	$40|\pm7/2\rangle + 28|\pm1/2\rangle + 17|\pm5/2\rangle + 13|\pm3/2\rangle$	3.5002	2.8433	0.1167	
5	1161	927	$46|\pm1/2\rangle + 29|\pm3/2\rangle + 15|\pm7/2\rangle + 10|\pm5/2\rangle$	0.7293	2.3728	4.1255	

total contribution of B_q^2, B_q^4, and B_q^6 terms to LF splitting: 94.5 %; angle between g_z axis of KD1 and Nd–N bond is 12.0°

Table S7c. Ligand-field splitting of Nd^{3+}-$^4I_{9/2}$ multiplet in $NdY_2N@C_{80}$ (conf **Y-1,** Sc instead of Y) computed at the CASSCF/RASSI level.

KD	exp. *E* cm^{-1}	calc. *E* cm^{-1}	% composition in $|J, m_J\rangle$ basis (J = 9/2)	g_x	g_y	g_z	ϕ°
1	0	0	$83|\pm9/2\rangle + 11|\pm5/2\rangle$	0.1072	0.1259	5.6874	0
2	420	372	$36|\pm7/2\rangle + 33|\pm5/2\rangle + 19|\pm3/2\rangle + 8|\pm9/2\rangle$	0.4722	0.8006	5.2289	
3	711	588	$37|\pm3/2\rangle + 31|\pm5/2\rangle + 17|\pm1/2\rangle + 8|\pm7/2\rangle$	0.8623	1.8237	4.0464	
4	1005	793	$39|\pm7/2\rangle + 28|\pm1/2\rangle + 15|\pm5/2\rangle + 15|\pm3/2\rangle$	3.3334	2.8496	0.3317	
5	1188	938	$49|\pm1/2\rangle + 25|\pm3/2\rangle + 17|\pm7/2\rangle + 10|\pm5/2\rangle$	0.8706	2.5878	3.8852	

total contribution of B_q^2, B_q^4, and B_q^6 terms to LF splitting: 94.6 %; angle between g_z axis of KD1 and Nd–N bond is 10.6°

Table S7d. Ligand-field splitting of Nd^{3+}-$^4I_{9/2}$ multiplet in $NdY_2N@C_{80}$ (conf **Y-1**) computed at the CASSCF/RASSI level.

KD	exp. *E* cm^{-1}	calc. *E* cm^{-1}	% composition in $|J, m_J\rangle$ basis (J = 9/2)	g_x	g_y	g_z	ϕ°
1	0	0	$83|\pm9/2\rangle + 10|\pm5/2\rangle$	0.1069	0.1251	5.6897	0
2	420	370	$38|\pm7/2\rangle + 33|\pm5/2\rangle + 17|\pm3/2\rangle + 9|\pm9/2\rangle$	0.4333	0.7055	5.3514	
3	711	597	$36|\pm3/2\rangle + 30|\pm5/2\rangle + 20|\pm1/2\rangle + 8|\pm7/2\rangle$	0.8595	2.0443	4.0268	
4	1005	815	$37|\pm7/2\rangle + 27|\pm1/2\rangle + 17|\pm5/2\rangle + 17|\pm3/2\rangle$	3.1997	2.9042	0.4781	
5	1188	956	$47|\pm1/2\rangle + 26|\pm3/2\rangle + 16|\pm7/2\rangle + 10|\pm5/2\rangle$	0.8611	2.6187	4.8452	

total contribution of B_q^2, B_q^4, and B_q^6 terms to LF splitting: 94.5 %; angle between g_z axis of KD1 and Nd–N bond is 10.8°

Table S8a. Ligand-field splitting of Nd^{3+}-$^4I_{9/2}$ multiplet in $NdLu_2N@C_{80}$ (conf **Lu-3,** Sc instead of Lu) computed at the CASSCF/RASSI level.

KD	exp. *E* cm^{-1}	calc. *E* cm^{-1}	% composition in $\|J, m_J\rangle$ basis (J = 9/2)	g_x	g_y	g_z	ϕ°
1	0	0	85|±9/2⟩ + 10|±5/2⟩	0.0036	0.0092	5.7520	0
2	510	410	48|±7/2⟩ + 24|±3/2⟩ + 23|±5/2⟩	0.2008	0.5116	4.8088	
3	775	652	35|±5/2⟩ + 28|±1/2⟩ + 25|±3/2⟩ + 9|±9/2⟩	0.0969	1.7825	3.9574	
4	958	882	35|±7/2⟩ + 28|±1/2⟩ + 24|±5/2⟩ + 11|±3/2⟩	0.4679	3.7226	2.4160	
5	1161	1001	40|±1/2⟩ + 39|±3/2⟩ + 13|±7/2⟩ + 9|±5/2⟩	4.0587	2.5187	0.7308	

total contribution of B_q^2, B_q^4, and B_q^6 terms to LF splitting: 93.6%; angle between g_z axis of KD1 and Nd–N bond is 7.6°

Table S8b. Ligand-field splitting of Nd^{3+}-$^4I_{9/2}$ multiplet in $NdLu_2N@C_{80}$ (conf **Lu-3,** Y instead of Lu) computed at the CASSCF/RASSI level.

KD	exp. *E* cm^{-1}	calc. *E* cm^{-1}	% composition in $\|J, m_J\rangle$ basis (J = 9/2)	g_x	g_y	g_z	ϕ°
1	0	0	84|±9/2⟩ + 10|±5/2⟩	0.0042	0.0081	5.7171	0
2	510	409	49|±7/2⟩ + 22|±5/2⟩ + 21|±3/2⟩	0.1517	0.4547	4.9043	
3	775	673	32|±5/2⟩ + 29|±1/2⟩ + 25|±3/2⟩ + 9|±9/2⟩	0.2695	2.0131	3.8157	
4	958	905	33|±7/2⟩ + 28|±1/2⟩ + 26|±5/2⟩ + 11|±3/2⟩	0.6272	3.6636	2.4075	
5	1161	1029	40|±3/2⟩ + 37|±1/2⟩ + 13|±7/2⟩ + 9|±5/2⟩	3.9209	2.6344	0.7860	

total contribution of B_q^2, B_q^4, and B_q^6 terms to LF splitting: 94.0 %; angle between g_z axis of KD1 and Nd–N bond is 7.7°

Table S8c. Ligand-field splitting of Nd^{3+}-$^4I_{9/2}$ multiplet in $NdY_2N@C_{80}$ (conf **Y-3,** Sc instead of Y) computed at the CASSCF/RASSI level.

KD	exp. *E* cm^{-1}	calc. *E* cm^{-1}	% composition in $\|J, m_J\rangle$ basis (J = 9/2)	g_x	g_y	g_z	ϕ°
1	0	0	84|±9/2⟩ + 11|±5/2⟩	0.0016	0.0190	5.7031	0
2	420	362	49|±7/2⟩ + 24|±3/2⟩ + 22|±5/2⟩	0.1614	0.4136	4.8780	
3	711	637	32|±1/2⟩ + 31|±5/2⟩ + 26|±3/2⟩ + 9|±9/2⟩	0.2653	2.1164	3.7799	
4	1005	875	34|±7/2⟩ + 30|±1/2⟩ + 28|±5/2⟩ + 7|±3/2⟩	0.7862	3.6437	2.2903	
5	1188	996	43|±3/2⟩ + 33|±1/2⟩ + 15|±7/2⟩ + 9|±5/2⟩	3.8398	2.5396	0.9997	

total contribution of B_q^2, B_q^4, and B_q^6 terms to LF splitting: 92.8%; angle between g_z axis of KD1 and Nd–N bond is 9.5°

Table S8d. Ligand-field splitting of Nd^{3+}-$^4I_{9/2}$ multiplet in $NdY_2N@C_{80}$ (conf **Y-3**) computed at the CASSCF/RASSI level.

KD	exp. *E* cm^{-1}	calc. *E* cm^{-1}	% composition in $\|J, m_J\rangle$ basis (J = 9/2)	g_x	g_y	g_z	ϕ°
1	0	0	82|±9/2⟩ + 12|±5/2⟩	0.0001	0.0193	5.6649	0
2	420	357	49|±7/2⟩ + 23|±3/2⟩ + 22|±5/2⟩ + 6|±9/2⟩	0.1232	0.3599	4.9815	
3	711	657	34|±1/2⟩ + 29|±5/2⟩ + 26|±3/2⟩ + 9|±9/2⟩	0.4064	2.3265	3.6440	
4	1005	901	33|±7/2⟩ + 31|±1/2⟩ + 29|±5/2⟩ + 7|±3/2⟩	0.9266	3.5722	2.2766	
5	1188	1028	44|±3/2⟩ + 31|±1/2⟩ + 16|±7/2⟩ + 9|±5/2⟩	3.7235	2.5832	1.0728	

total contribution of B_q^2, B_q^4, and B_q^6 terms to LF splitting: 92.6%; angle between g_z axis of KD1 and Nd–N bond is 9.8°

Table S9. Ligand-field splitting (Δ_{LF}) of Nd^{3+} $^4I_{9/2}$ and $^4F_{3/2}$ multiplets, their energy difference (ΔE_{aver}), and the ratio of the energy difference to that in the free ion ($\beta_{spec} = \Delta E_{aver}/\Delta E_{free\ ion}$).[a]

compound [b]	$\Delta_{LF}(^4I_{9/2})$	$\Delta_{LF}(^4F_{3/2})$	$\Delta E_{aver}(^4F_{3/2}–^4I_{9/2})^c$	β_{spec}	Ref.
**Nd^{3+} free ion**	–	–	11699	1.000	14
**Nd-EMFs:**					
$NdLu_2N@C_{80}$	1161	440	10445	0.893	t.w.
$NdY_2N@C_{80}$	1188	440	10456	0.894	t.w.
$NdSc_2N@C_{80}$	1061	410	10558	0.903	t.w.
**$Nd(CpX)_3$:**					
$Nd(\eta^5\text{-}C_5Me_4H)_3$	1000	710	10925	0.934	16
$Nd(\eta^5\text{-}C_5Me_5)_3$	861	635	10928	0.934	15
$Nd(\eta^5\text{-}C_5H_4{}^tBu)_3$	1029	684	10951	0.936	17
$Nd(\eta^5\text{-}C_5H_4SiMe_3)_3$	1031	696	10951	0.936	17
**Nd-SMMs:**					
$[Nd_2(2\text{-}FBz)_4(NO_3)_2(phen)_2]$	–	–	11086	0.948	18
$[Nd_2(\mu_2\text{–}9\text{-}AC)_4(9\text{-}AC)_2(bipy)_2]$	414	–	11183	0.956	19
$[Nd_2(\mu_2\text{–}9\text{-}AC)_4(9\text{-}AC)_2(phen)_2]$	415	–	11190	0.956	20
$[Nd(ntfa)_3(5,5'\text{-}Me_2bipy)]$	–	–	11198	0.957	21
$[Nd(TTA)_3(MeOH)_2]\cdot0.5Azo\text{-}py$	–	314	11207	0.958	22
$Nd(pzdo)[Co^{III}(CN)_6]^{3-}$	–	–	11211	0.958	23
$[Nd(ntfa)_3(phen)]$	–	–	11236	0.960	21
$[Nd(ntfa)_3(bipy)]$	–	–	11236	0.960	21
$[Nd(ntfa)_3(4,4'\text{-}Mt_2bipy)]$	–	–	11236	0.960	21
$\{[Nd_4(H_2O)_{17}(pzdo)_5][Mo^{IV}(CN)_8]_3\}\cdot9H_2O$	–	–	11236	0.960	24
Nd-arsenotungstate POM	–	–	11249	0.962	25
$Nd(Tp)_3$	358	110	11274	0.964	26
$\{[Nd_4(H_2O)_{17}(pzdo)_5][W^{IV}(CN)_8]_3\}\cdot9H_2O$	–	–	11287	0.965	24
$[Na_2NdL'_4(OTf)DMF]$	425	95	11304	0.966	27
$[Zn(\mu\text{-}L)(\mu\text{-}OAc)Nd(NO_3)_2]\cdot CH_3CN$	459	–	11343	0.970	28
$[Nd_2(\mu\text{-}2\text{-}FBz)_2(2\text{-}FBz)_4(terpy)_2]$	–	–	11390	0.974	29
NdMurexAnhy	–	–	11494	0.982	30
**Nd^{3+} in garnets and salts:**					
$Nd:Y_2O_3$	645	196	11028	0.943	31
Nd_2S_3	385	38	11053	0.945	32
$Nd:Y_3Al_2(AlO_4)_3$ [YAG]	848	92	11167	0.955	33
$Nd:Y_3Sc_2(AlO_4)_3$ [YSAG]	823	100	11189	0.956	34
$Nd:Gd_3Sc_2(GaO_4)_3$ [GSGG]	763	62	11203	0.958	34
$Nd:La_3Lu_2(GaO_4)_3$ [LLGG]	710	122	11221	0.959	34
$Nd:LaCl_3$	249	30	11293	0.965	35
$[Nd(H_2O)_9](C_2H_5SO_4)_3$	311	15	11367	0.972	36
$Nd:LaF_3$	500	42	11418	0.976	37

[a] Δ_{LF} and $\Delta E_{aver}(^4F_{3/2}–^4I_{9/2})$ values are determined by optical spectroscopy and given in cm^{-1};

[b] 2-FBz = 2-fluorobenzoate; phen = 1,10-phenanthroline; 9-AC = 9-anthracenecarboxylate; bipy = 2,2′-bipyridine; ntfa = 4,4,4-trifluoro-1-(naphthalen-2-yl)butane-1,3-dionate; 5,5′-Me_2bipy = 5,5′-dimethyl-2,2′-dipyridine; TTA = trifluoro-1-(2-thienyl)-1,3-butanedione; Azo-py = 4,4′-azopyridine; 4,4′-Mt_2bipy = 4,4′-dimethoxy-2,2′-dipyridine; pzdo = pyrazine-N,N′-dioxide; Nd-arsenotungstate POM = $K_{14}Na_6H_4[\{(As_2W_{19}O_{67}(H_2O))Nd(H_2O)_2\}_2(C_2O_4)]\cdot64H_2O$ polyoxometalate; Tp = hydrotris(1-pyrazolyl)borato; L = N,N′-dimethyl-N,N′-bis(2-hydroxy-3-formyl-5-bromo-benzyl) ethylenediamine; L′ = N-(diphenylphosphoryl)pyrazine-2-carboxamide; terpy = 2,2′:6′,2′′-terpyridine; Murex = murexide or 2,6-dioxo-5-[(2,4,6-trioxo-5-hexahydropyrimi-dinylidene)amino]-3H-pyrimidin-4-olate; Anhy = anhydrous form;

[c] $\Delta E_{aver}(^4F_{3/2}–^4I_{9/2})$ is calculated using barycenters when LF splitting is available for $^4I_{9/2}$ and $^4F_{3/2}$ multiplets; else, when the LF splitting is not resolved, $\Delta E_{aver}(^4F_{3/2}–^4I_{9/2})$ is calculated using position of the $^4F_{3/2}\rightarrow{}^4I_{9/2}$ PL band.

XMCD measurements and sum rule analysis

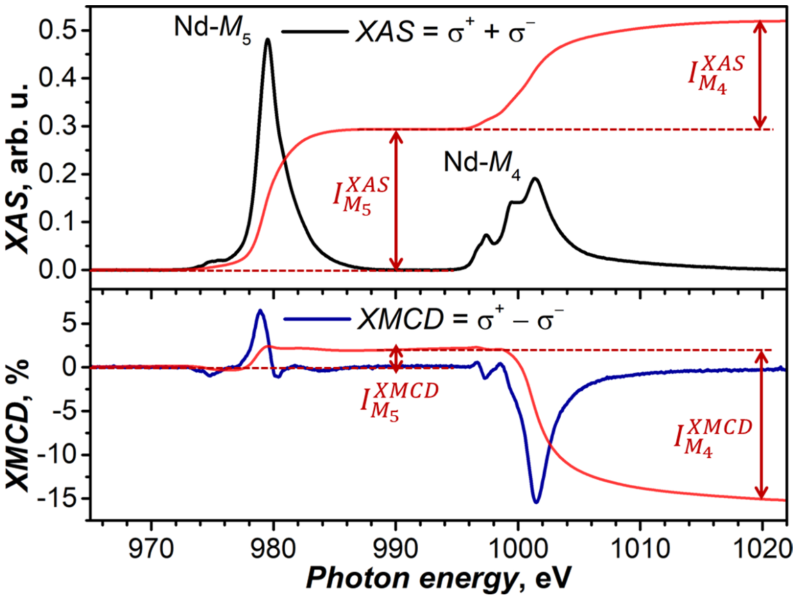

Figure S20. XAS (upper panel) and XMCD (lower panel) spectra of $NdSc_2N@C_{80}$ at the Nd-$M_{4,5}$ edges (3d→4f), T ≈ 5.5 K, μ_0H = 6 T. Left-hand and right-hand polarized XAS spectra are denoted as σ^+ and σ^-, non-polarized XAS spectrum is their sum, while XMCD (in %) is their difference normalized to the maximum of XAS. Red lines are integrated XAS and XMCD curves, also shown is the definition of integrals ($I_{M_{4,5}}^{XAS}$ and $I_{M_{4,5}}^{XMCD}$) used in the sum rule analysis, see Eq. S1

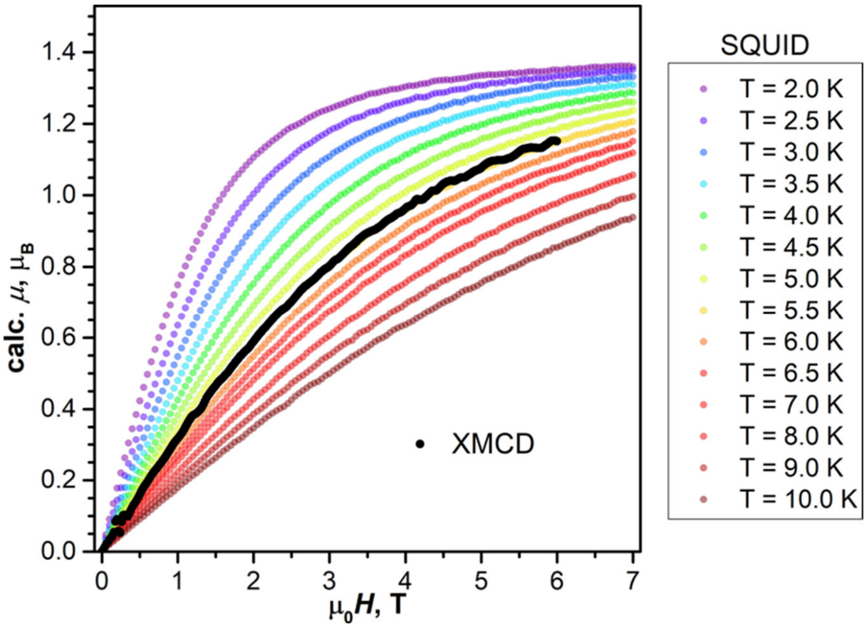

Figure S21. Magnetization curve of $NdSc_2N@C_{80}$ measured by XMCD (black dots, arbitrary units) overlaid over magnetization curves measured by SQUID magnetometry (colored dots). The best match between the shapes of XMCD and SQUID curves is obtained for T = 5.5 K. We thus assume that the sample temperature during XMCD measurements was T ≈ 5.5±0.5 K.

In the unpolarized X-ray absorption spectrum at the Nd-$M_{4,5}$ absorption edges (3d→4f excitations), $NdSc_2N@C_{80}$ shows typical XAS features of Nd(III) (Fig. S20). When a magnetic field is applied, circular polarized XAS develops a dichroism proportional to the sample magnetization in the direction of the beam (which was kept parallel to the field in our measurements) (Fig. S20). The maximum of the XMCD signal at 1001.5 eV was used to follow magnetization of $NdSc_2N@C_{80}$ during magnetic field ramps from +6 T to –6 T and from −6 T to +6 T with the sweep rate of 2 T/min. No magnetic hysteresis was observed. The obtained magnetization curve averaged over four segments ([–6→0], [0→6], [6→0], and [0→–6] T) is shown in Figure S21. The shape of the curve indicates that the sample magnetization was not saturated at 6 T. Precise temperature estimation on the sample was done by comparison of the magnetization curve measured by XMCD to the curves measured with SQUID magnetometry with the step of 0.5 K. The best match of the curves was obtained for 5.5 K. Thus, we assume that during XMCD measurements the sample had a temperature of $T \approx 5.5 \pm 0.5$ K.

Magnetic moment along direction of the beam, μ_z, can be calculated as $\mu_\mathrm{z} = \left(\langle \hat{L}_\mathrm{z} \rangle + 2\langle \hat{S}_\mathrm{z} \rangle\right)\mu_\mathrm{B}/\hbar$, where $\langle \hat{L}_\mathrm{z} \rangle$ and $\langle \hat{S}_\mathrm{z} \rangle$ are expectation values of angular and spin momentum operators. These values can be estimated from XAS and XMCD spectra using sum rules:[38, 39]

$$\langle \hat{L}_\mathrm{z} \rangle = 3(14 - n_\mathrm{4f}) \frac{I_{M_5}^{XMCD} + I_{M_4}^{XMCD}}{3/2(I_{M_5}^{XAS} + I_{M_4}^{XAS})} \qquad (\mathrm{Eq.\,S1a})$$

$$\langle \hat{S}_\mathrm{z} \rangle + 3\langle \hat{T}_\mathrm{z} \rangle = 3/2(14 - n_\mathrm{4f}) \frac{I_{M_5}^{XMCD} - 3/2 I_{M_4}^{XMCD}}{3/2(I_{M_5}^{XAS} + I_{M_4}^{XAS})} \frac{1}{C_\mathrm{corr}^\mathrm{Ln}} \qquad (\mathrm{Eq.\,S1b})$$

Where n_4f is the number of 4f electrons (3 for Nd), integrals such as $I_{M_5}^{XAS}$ are defined in Figure S20, $\langle \hat{T}_\mathrm{z} \rangle$ stands for the expectation values of the magnetic dipole operator $\hat{T}_\mathrm{z}$, and $C_\mathrm{corr}^\mathrm{Ln}$ is the correction factor from Ref. [40], which takes into account the deviation of the sum rules for spin momentum caused by partial mixing of $3d_{3/2}$ and $3d_{5/2}$ multiplets (for Nd^{3+}, the value is 2.076). $\langle \hat{S}_\mathrm{z} \rangle$ can be determined from Eq. S1b using the relation $\langle \hat{T}_\mathrm{z} \rangle / \langle \hat{S}_\mathrm{z} \rangle = 0.315$ for Nd^{3+} tabulated in Ref. [40].

Using Eq. S1a and S1b, we obtained expectation values of angular and spin momentum operators, $\langle \hat{L}_\mathrm{z} \rangle$ = 2.02 μ_B and $\langle \hat{S}_\mathrm{z} \rangle$ = –0.45 μ_B, and the magnetic moment along the beam direction, μ_z = 1.11 μ_B. The uncertainty in determined μ_z is mainly caused by ambiguities in integration limits for Nd-M_4 edge and is estimated to be less than ±0.1 μ_B. The obtained ratio $\langle \hat{S}_\mathrm{z} \rangle / \langle \hat{L}_\mathrm{z} \rangle$ = –0.22 is close to –0.25, a theoretical expectation for the f^3 system.

Magnetization relaxation times of $NdSc_2N@C_{80}$

In AC magnetometry studies, the in-phase and out-of-phase susceptibility, $\chi'(\nu)$ and $\chi''(\nu)$, were measured as a function of oscillation frequency ν and then fitted with the generalized Debye model:

$$\chi'(\nu) = \chi_S + (\chi_T - \chi_S)\frac{1 + (\nu\tau_\mathrm{M})^{1-\alpha}\sin\left(\frac{\pi\alpha}{2}\right)}{1 + 2(\nu\tau_\mathrm{M})^{1-\alpha}\sin\left(\frac{\pi\alpha}{2}\right) + (\nu\tau_\mathrm{M})^{2-2\alpha}}$$

$$\chi''(\nu) = (\chi_T - \chi_S)\frac{(\nu\tau_\mathrm{M})^{1-\alpha}\cos\left(\frac{\pi\alpha}{2}\right)}{1 + 2(\nu\tau_\mathrm{M})^{1-\alpha}\sin\left(\frac{\pi\alpha}{2}\right) + (\nu\tau_\mathrm{M})^{2-2\alpha}} + b\nu$$

The linear term ($b\nu$) is included to correct for a linear background that was found in $\chi''(\nu)$ measurements at high frequency (the background has noticeable contribution to χ'' curves only above > 100 Hz). Relaxation times τ_M and parameters α determined from the fits are listed in Tables S10-S11. Fitted curves along with the measured points are shown in Fig. S22 and Fig. 8 in the main text.

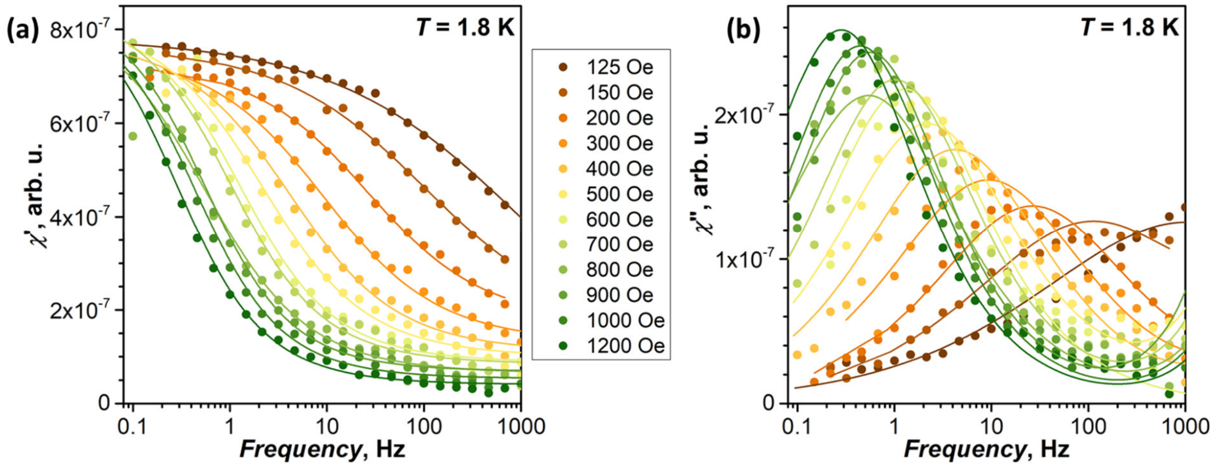

Figure S22. AC magnetometry measurements of in-phase χ' (a) and out-of-phase χ'' (b) magnetic susceptibility of $NdSc_2N@C_{80}$ in different magnetic fields at constant temperature of 1.8 K; dots are experimental data, lines are fits with generalized Debye model, from which relaxation times are extracted.

Table S10. Magnetization relaxation times of $NdSc_2N@C_{80}$ and α parameters determined by AC magnetometry at different fields

H, Oe	*T* = 1.8 K			
	**τ, s**	**±τ, s**	***α***	**±*α***
125	1.53E-4	6.4E-5	0.60	0.02
150	1.53E-3	2.6E-4	0.51	0.02
200	6.02E-3	3.1E-4	0.42	0.01
300	1.71E-2	1.4E-3	0.41	0.03
400	3.78E-2	2.7E-3	0.39	0.02
500	7.21E-2	6.3E-3	0.37	0.03
600	1.24E-1	8.2E-3	0.33	0.02
700	1.56E-1	1.1E-2	0.33	0.02
800	2.93E-1	1.4E-2	0.37	0.01
900	2.85E-1	1.6E-2	0.30	0.02
1000	3.59E-1	2.6E-2	0.30	0.02
1200	5.61E-1	4.3E-2	0.31	0.02

Table S11. Magnetization relaxation times of $NdSc_2N@C_{80}$ and α parameters determined by AC magnetometry at different temperatures

T, K	$\mu_0 H$ = 0.2 T				$\mu_0 H$ = 0.04 T				$\mu_0 H$ = 0.02 T			
	**τ, s**	**±τ, s**	***α***	**±*α***	**τ, s**	**±τ, s**	***α***	**±*α***	**τ, s**	**±τ, s**	***α***	**±*α***
1.80					3.8E-2	2.7E-3	0.39	0.02	6.0E-3	3.1E-4	0.42	0.01
1.90	7.7E-1	6.2E-2	0.25	0.02	3.1E-2	1.4E-3	0.37	0.01	3.2E-3	1.8E-4	0.45	0.01
2.00	5.6E-1	3.4E-2	0.24	0.02	2.8E-2	1.3E-3	0.34	0.02	3.5E-3	2.0E-4	0.42	0.01
2.27	2.3E-1	1.1E-2	0.21	0.02	2.1E-2	8.5E-4	0.32	0.02	3.8E-3	1.3E-4	0.37	0.01
2.50	1.2E-1	3.5E-3	0.20	0.01	1.5E-2	6.6E-4	0.32	0.02	2.1E-3	1.8E-4	0.41	0.02
2.73	6.3E-2	1.5E-3	0.19	0.01	1.2E-2	3.7E-4	0.25	0.01	2.9E-3	9.9E-5	0.33	0.01
3.00	3.5E-2	7.0E-4	0.21	0.01	8.3E-3	2.5E-4	0.24	0.01	1.8E-3	1.1E-4	0.35	0.01
3.45	1.2E-2	3.0E-4	0.18	0.01	4.9E-3	1.5E-4	0.19	0.01	1.9E-3	7.9E-5	0.22	0.01
4.00	4.7E-3	1.0E-4	0.15	0.01	2.4E-3	6.0E-5	0.14	0.01	9.6E-4	7.3E-5	0.19	0.02
4.56	1.9E-3	4.9E-5	0.13	0.01	1.2E-3	3.2E-5	0.12	0.01	6.7E-4	3.3E-5	0.18	0.02
4.80	1.4E-3	4.6E-5	0.12	0.01								
5.00	1.1E-3	4.0E-5	0.08	0.02	7.4E-4	4.2E-5	0.10	0.02	3.0E-4	2.3E-5	0.17	0.02
5.10	9.8E-4	3.4E-5	0.07	0.01								
6.00	4.0E-4	4.9E-5	0.01	0.04	2.3E-4	1.2E-5	0.05	0.02	2.0E-4	2.5E-5	0.03	0.04
6.00	3.3E-4	2.9E-5	0.05	0.02								
7.00	2.0E-4	3.0E-5	0.00	0.05	1.4E-4	2.6E-5	0.00	0.04				

Coexistence of tunneling and thermal relaxation processes results in rather large values of the α parameter, which tend to decrease with the increase of the external field because of the gradual suppression of the QTM mechanism.

References

1. Mueller, U.; Förster, R.; Hellmig, M.; Huschmann, F. U.; Kastner, A.; Malecki, P.; Pühringer, S.; Röwer, M.; Sparta, K.; Steffien, M.; Ühlein, M.; Wilk, P.; Weiss, M. S., The macromolecular crystallography beamlines at BESSY II of the Helmholtz-Zentrum Berlin: Current status and perspectives. *Eur. Phys. J. Plus* **2015,** *130* (7), 141.
2. Kabsch, W., XDS. *Acta Cryst. D* **2010,** *66* (2), 125-132.
3. Sparta, K. M.; Krug, M.; Heinemann, U.; Mueller, U.; Weiss, M. S., XDSAPP2.0. *J. Appl. Crystallogr.* **2016,** *49* (3), 1085-1092.
4. Sheldrick, G., Crystal structure refinement with SHELXL. *Acta Cryst. C* **2015,** *71* (1), 3-8.
5. Hafner, J., Ab-initio simulations of materials using VASP: Density-functional theory and beyond. *J. Comput. Chem.* **2008,** *29* (13), 2044-2078.
6. Kresse, G.; Hafner, J., Ab initio molecular dynamics for liquid metals. *Phys. Rev. B* **1993,** *47* (1), 558-561.
7. Kresse, G.; Joubert, D., From ultrasoft pseudopotentials to the projector augmented-wave method. *Phys. Rev. B* **1999,** *59* (3), 1758-1775.
8. Perdew, J. P.; Burke, K.; Ernzerhof, M., Generalized gradient approximation made simple. *Phys. Rev. Lett.* **1996,** *77* (18), 3865-3868.
9. Grimme, S., Density functional theory with London dispersion corrections. *WIREs Comput. Mol. Sci.* **2011,** *1* (2), 211-228.
10. Aquilante, F.; Autschbach, J.; Baiardi, A.; Battaglia, S.; Borin, V. A.; Chibotaru, L. F.; Conti, I.; Vico, L. D.; Delcey, M.; Galván, I. F.; Ferré, N.; Freitag, L.; Garavelli, M.; Gong, X.; Knecht, S.; Larsson, E. D.; Lindh, R.; Lundberg, M.; Malmqvist, P. Å.; Nenov, A.; Norell, J.; Odelius, M.; Olivucci, M.; Pedersen, T. B.; Pedraza-González, L.; Phung, Q. M.; Pierloot, K.; Reiher, M.; Schapiro, I.; Segarra-Martí, J.; Segatta, F.; Seijo, L.; Sen, S.; Sergentu, D.-C.; Stein, C. J.; Ungur, L.; Vacher, M.; Valentini, A.; Veryazov, V., Modern quantum chemistry with [Open]Molcas. *J. Chem. Phys.* **2020,** *152* (21), 214117.
11. Chibotaru, L. F.; Ungur, L., Ab initio calculation of anisotropic magnetic properties of complexes. I. Unique definition of pseudospin Hamiltonians and their derivation. *J. Chem. Phys.* **2012,** *137* (6), 064112.
12. Chilton, N. F.; Anderson, R. P.; Turner, L. D.; Soncini, A.; Murray, K. S., PHI: A powerful new program for the analysis of anisotropic monomeric and exchange-coupled polynuclear d- and f-block complexes. *J. Comput. Chem.* **2013,** *34* (13), 1164-1175.
13. Barla, A.; Nicolás, J.; Cocco, D.; Valvidares, S. M.; Herrero-Martín, J.; Gargiani, P.; Moldes, J.; Ruget, C.; Pellegrin, E.; Ferrer, S., Design and performance of BOREAS, the beamline for resonant X-ray absorption and scattering experiments at the ALBA synchrotron light source. *J. Synchrot. Radiat.* **2016,** *23* (6), 1507-1517.
14. Wyart, J.-F.; Meftah, A.; Bachelier, A.; Sinzelle, J.; Tchang-Brillet, W.-Ü. L.; Champion, N.; Spector, N.; Sugar, J., Energy levels of $4f^3$ in the Nd^{3+} free ion from emission spectra. *J. Phys. B: At. Mol. Opt. Phys* **2006,** *39* (5), L77-L82.
15. Amberger, H.-D.; Reddmann, H.; Mueller, T. J.; Evans, W. J., Electronic structures of organometallic complexes of f elements LXXV: Parametric analysis of the crystal field splitting pattern of $Nd(\eta^5\text{-}C_5Me_5)_3$. *J. Organomet. Chem.* **2011,** *696* (15), 2829-2836.
16. Amberger, H.-D.; Reddmann, H., Zur Elektronenstruktur metallorganischer Komplexe der f-Elemente. 65 Erstmalige Beobachtung des linearen Dichroismus bei einem homoleptischen metallorganischen π-Komplex der f-Elemente: Tris(η^5-tetramethylcyclopenta-dienyl)neodym(III). *Z. Anorg. Allg. Chem.* **2007,** *633* (3), 443-452.
17. Jank, S.; Reddmann, H.; Amberger, H.-D.; Apostolidis, C., Zur Elektronenstruktur metallorganischer Komplexe der f-Elemente LIX. Spektrochemische und nephelauxetische Effekte sowie experimentorientierte MO-Schemata (im f-Bereich) pseudo-trigonal-planarer (substituierter) Tris(η^5-

cyclopentadienyl)neodym(III)-Komplexe und eines THF-Monoaddukts. *J. Organomet. Chem.* **2004,** *689* (20), 3143-3157.
18. Casanovas, B.; Font-Bardía, M.; Speed, S.; El Fallah, M. S.; Vicente, R., Field-Induced SMM and Visible/NIR-Luminescence Behaviour of Dinuclear Ln^{III} Complexes with 2-Fluorobenzoate. *Eur. J. Inorg. Chem.* **2018,** *2018* (18), 1928-1937.
19. Casanovas, B.; Speed, S.; Maury, O.; El Fallah, M. S.; Font-Bardía, M.; Vicente, R., Dinuclear Ln^{III} Complexes with 9-Anthracenecarboxylate Showing Field-Induced SMM and Visible/NIR Luminescence. *Eur. J. Inorg. Chem.* **2018,** *2018* (34), 3859-3867.
20. Casanovas, B.; Speed, S.; Maury, O.; Font-Bardía, M.; Vicente, R., Homodinuclear lanthanide 9-anthracenecarboxylate complexes: Field induced SMM and NIR-luminescence. *Polyhedron* **2019,** *169*, 187-194.
21. Vicente, R.; Tubau, À.; Speed, S.; Mautner, F. A.; Bierbaumer, F.; Fischer, R. C.; Massoud, S. S., Slow magnetic relaxation and luminescence properties in neodymium(iii)-4,4,4-trifluoro-1-(2-naphthyl)butane-1,3-dionato complexes incorporating bipyridyl ligands. *New J. Chem.* **2021,** *45* (32), 14713-14723.
22. Kumar, K.; Abe, D.; Komori-Orisaku, K.; Stefańczyk, O.; Nakabayashi, K.; Shakirova, J. R.; Tunik, S. P.; Ohkoshi, S.-i., Neodymium β-diketonate showing slow magnetic relaxation and acting as a ratiometric thermometer based on near-infrared emission. *RSC Adv.* **2019,** *9* (41), 23444-23449.
23. Chorazy, S.; Charytanowicz, T.; Wang, J.; Ohkoshi, S.-i.; Sieklucka, B., Hybrid organic–inorganic connectivity of Nd^{III}(pyrazine-N,N′-dioxide)$[Co^{III}(CN)_6]^{3-}$ coordination chains for creating near-infrared emissive Nd(iii) showing field-induced slow magnetic relaxation. *Dalton Trans.* **2018,** *47* (24), 7870-7874.
24. Jankowski, R.; Zakrzewski, J. J.; Zychowicz, M.; Wang, J.; Oki, Y.; Ohkoshi, S.-i.; Chorazy, S.; Sieklucka, B., SHG-active NIR-emissive molecular nanomagnets generated in layered neodymium(iii)–octacyanidometallate(iv) frameworks. *J. Mater. Chem. C* **2021,** *9* (33), 10705-10717.
25. Chen, H.; Sun, L.; Zheng, K.; Zhang, J.; Ma, P.; Wang, J.; Niu, J., Oxalate-bridging Nd^{III}-based arsenotungstate with multifunctional NIR-luminescence and magnetic properties. *Dalton Trans.* **2022,** *51* (26), 10257-10265.
26. Reddmann, H.; Apostolidis, C.; Walter, O.; Amberger, H.-D., Zur Elektronenstruktur hochsymmetrischer Verbindungen der f-Elemente. 40. Parametrische Analyse des Kristallfeld-Aufspaltungsmusters von Tris(hydrotris(1-pyrazolyl)borato)neodym(III). *Z. Anorg. Allg. Chem.* **2006,** *632* (8-9), 1405-1408.
27. Pham, Y. H.; Trush, V. A.; Korabik, M.; Amirkhanov, V. M.; Gawryszewska, P., Nd^{3+} and Yb^{3+} complexes with N-(diphenylphosphoryl)pyrazine-2-carboxamide as UV-NIR radiation converters and single-ion magnets. *Dyes Pigment.* **2021,** *186*, 108986.
28. Oyarzabal, I.; Artetxe, B.; Rodríguez-Diéguez, A.; García, J. Á.; Seco, J. M.; Colacio, E., A family of acetato-diphenoxo triply bridged dimetallic $Zn^{II}Ln^{III}$ complexes: SMM behavior and luminescent properties. *Dalton Trans.* **2016,** *45* (23), 9712-9726.
29. Casanovas, B.; Speed, S.; Vicente, R.; Font-Bardía, M., Sensitization of visible and NIR emitting lanthanide(III) ions in a series of dinuclear complexes of formula $[Ln_2(\mu\text{-}2\text{-}FBz)_2(2\text{-}FBz)_4(terpy)_2]\cdot 2(2\text{-}HFBz)\cdot 2(H_2O)$. *Polyhedron* **2019,** *173*, 114113.
30. Huang, G.; Calvez, G.; Suffren, Y.; Daiguebonne, C.; Freslon, S.; Guillou, O.; Bernot, K., Closing the Circle of the Lanthanide-Murexide Series: Single-Molecule Magnet Behavior and Near-Infrared Emission of the Nd^{III} Derivative. *Magnetochemistry* **2018,** *4* (4), 44.
31. Chang, N. C., Energy Levels and Crystal-Field Splittings of Nd^{3+} in Yttrium Oxide. *J. Chem. Phys.* **1966,** *44* (10), 4044-4050.
32. Gruber, J. B.; Leavitt, R. P.; Morrison, C. A., Absorption spectra, energy levels, and crystal-field analysis of trivalent neodymium in the γ phase of neodymium sesquisulfide ($\gamma\text{-}Nd_2S_3$). *J. Chem. Phys.* **1983,** *79* (4), 1664-1668.

33. Koningstein, J. A.; Geusic, J. E., Energy Levels and Crystal-Field Calculations of Neodymium in Yttrium Aluminum Garnet. *Phys. Rev.* **1964,** *136* (3A), A711-A716.
34. Gruber, J. B.; Hills, M. E.; Allik, T. H.; Jayasankar, C. K.; Quagliano, J. R.; Richardson, F. S., Comparative analysis of Nd^{3+} ($4f^3$) energy levels in four garnet hosts. *Phys. Rev. B* **1990,** *41* (12), 7999-8012.
35. Crosswhite, H. M.; Crosswhite, H.; Kaseta, F. W.; Sarup, R., The spectrum of Nd^{3+}:$LaCl_3$ *J. Chem. Phys.* **1976,** *64* (5), 1981-1985.
36. Hammond, R. M.; Reid, M. F.; Richardson, F. S., Comparison of crystal field parameters for $Ln(C_2H_5SO_4)_3{\cdot}9H_2O$ and $Na_3(Ln(C_4H_4O_5)_3){\cdot}2NaClO_4{\cdot}6H_2O$ systems. *J. Less-Common Met.* **1989,** *148* (1), 311-319.
37. Carnall, W. T.; Crosswhite, H.; Crosswhite, H. M. *Energy level structure and transition probabilities in the spectra of the trivalent lanthanides in* LaF_3; United States, 1978; p DOI: 10.2172/6417825.
38. Thole, B. T.; Carra, P.; Sette, F.; van der Laan, G., X-ray circular dichroism as a probe of orbital magnetization. *Phys. Rev. Lett.* **1992,** *68* (12), 1943-1946.
39. Carra, P.; Thole, B. T.; Altarelli, M.; Wang, X., X-ray circular dichroism and local magnetic fields. *Phys. Rev. Lett.* **1993,** *70* (5), 694-697.
40. Teramura, Y.; Tanaka, A.; Thole, B. T.; Jo, T., Effect of Coulomb Interaction on the X-Ray Magnetic Circular Dichroism Spin Sum Rule in Rare Earths. *J. Phys. Soc. Jpn.* **1996,** *65* (9), 3056-3059.
